# Plant genome evolution in the genus *Eucalyptus* is driven by structural rearrangements that promote sequence divergence

**DOI:** 10.1101/gr.277999.123

**Published:** 2024-04

**Authors:** Scott Ferguson, Ashley Jones, Kevin Murray, Rose Andrew, Benjamin Schwessinger, Justin Borevitz

**Affiliations:** 1 Research School of Biology, Australian National University, Canberra, Australian Capital Territory, 2601, Australia;; 2 Weigel Department, Max Planck Institute for Biology Tübingen, 72076 Tübingen, Germany;; 3 Botany & N.C.W. Beadle Herbarium, School of Environmental and Rural Science, University of New England, Armidale, New South Wales 2351, Australia

## Abstract

Genomes have a highly organized architecture (nonrandom organization of functional and nonfunctional genetic elements within chromosomes) that is essential for many biological functions, particularly gene expression and reproduction. Despite the need to conserve genome architecture, a high level of structural variation has been observed within species. As species separate and diverge, genome architecture also diverges, becoming increasingly poorly conserved as divergence time increases. However, within plant genomes, the processes of genome architecture divergence are not well described. Here we use long-read sequencing and de novo assembly of 33 phylogenetically diverse, wild and naturally evolving *Eucalyptus* species, covering 1–50 million years of diverging genome evolution to measure genome architectural conservation and describe architectural divergence. The investigation of these genomes revealed that following lineage divergence, genome architecture is highly fragmented by rearrangements. As genomes continue to diverge, the accumulation of mutations and the subsequent divergence beyond recognition of rearrangements become the primary driver of genome divergence. The loss of syntenic regions also contribute to genome divergence but at a slower pace than that of rearrangements. We hypothesize that duplications and translocations are potentially the greatest contributors to *Eucalyptus* genome divergence.

Genomes from all kingdoms are highly organized but vary greatly in their structural architecture (Koonin 2009). Within eukaryotic genomes, genome architecture refers to the nonrandom organization of functional and nonfunctional genetic elements within chromosomes (genes, regulatory regions, small RNAs, transposons, pseudogenes, introns, centromeres, telomeres, etc.) and is critical for many biological functions, in particular reproduction and gene expression. However, the conservation and divergence of genome architecture or structure among a group of radiating plant species that share a common karyotype have not been well described.

For effective recombination during meiosis and the production of viable reproducing offspring, the genome architecture of both parental haplotypes must be highly similar. Changes to the genetic architecture can result in reproductive isolation/incompatibility or nonviable gametes (Hardigan et al. 2020; Simakov et al. 2020). Therefore, a common genome architecture within individuals of a breeding population tends to be highly conserved, except at some loci with high diversity (Jiao and Schneeberger 2020). Similarly, for expression of a gene to be correctly regulated, it must be placed on a chromosome alongside the required promoters, enhancers, and inhibitors. The 3D organization of the surrounding chromatin must permit physical access to allow transcription (Heng et al. 2004; Dixon et al. 2016; Oudelaar and Higgs 2021).

Despite this functional need for structural conservation, some structural differences are known to exist between genomes within species. The extent to which reproductively compatible genomes are structurally different is an open area of research; however, several studies have shown genomes with a surprising amount of structural differences to be reproductively compatible (Lin and Gokcumen 2019; Alonge et al. 2020; Jiao and Schneeberger 2020; Tang et al. 2022). Between diverged species, genomes share less of their architecture than genomes within species, but typically genome architecture is conserved in proportion to phylogenetic distance (Luo et al. 2020; Weissensteiner et al. 2020; Derežanin et al. 2022; Ruggieri et al. 2022) and becomes poorly conserved at larger evolutionary distances (Koonin 2009).

However, genomes have often, but not always, been viewed as containers to hold genes (Heng 2009; Marques et al. 2019). The legacy of a gene-centric genome has persisted owing to the modern synthesis (Crkvenjakov and Heng 2022) and the highly influential work of Dawkins (1976) and others. Guided by an evolutionary view dominated by genes and gene variants, many genomes from various species have been sequenced and, by identifying their genes and gene variants, have provided us with a better understanding of the processes of evolution, divergence, and speciation (Rellstab et al. 2015; Schumer et al. 2015; Meier et al. 2017). However, a heavily gene-centric view may also limit our understanding (Heng 2009). This heavily gene variant–based view of evolution was common until recent advances in long-read sequencing technologies enabled genome-wide investigations into genome architecture (Amarasinghe et al. 2020). Larger structural genome changes were thought to be rare, and as such, genomes have been treated as largely structurally static, with individuals typically conceptualized as differing mostly by single-nucleotide polymorphisms (SNPs) (Feulner and De-Kayne 2017). Pangenome studies by using a collection of genes or sequences in a population or species (Bayer et al. 2020; Lei et al. 2021) have revealed a significant amount of structural variation within genomes (Torkamaneh et al. 2021; Tang et al. 2022; Li et al. 2023).

Shared genome architecture is measured by synteny. Synteny is the conservation of both the order and sequence of homologous chromosomes between genomes (Passarge et al. 1999; Dawson et al. 2007; Heger and Ponting 2007). Synteny can refer both to individual genome regions or to the aggregate when comparing whole genomes. A genome pair with a large proportion of syntenic loci can be said to be more syntenic than a genome pair with a small proportion of syntenic loci. Synteny can become disrupted by the loss, gain, duplication, rearrangement, or divergence of existing sequences. Rearrangements can occur as inversions, translocations, and duplications, altering the order of sequences within chromosomes while maintaining gene content, and are often labeled as structural variants (SVs) (Rieseberg 2001). Species-specific sequences resulting from the insertion, deletion, or localized divergence of sequence appear as unaligned regions when genomes are analyzed. The true origin of unaligned regions is more difficult to infer than rearrangements or syntenic regions (Weisman et al. 2020).

Crucial to the study of plant genome evolution is study group choice. The ideal study group would be naturally evolving, would have low prezygotic reproductive barriers, would be highly specious, and would exist over a wide and variable evolutionary range. *Eucalyptus*—with more than 800 wild and undomesticated species that exist across a wide geographic and environmental range (Potts and Wiltshire 1997; Booth et al. 2015; Supple et al. 2018), retain a conserved karyotype (Grattapaglia et al. 2015; Butler et al. 2017), are pollinated by generalist pollinators (Pfeilsticker et al. 2023), are capable of wide-ranging dispersal of genetic material (Bezemer et al. 2016; Murray et al. 2019), and span 50 million years of divergent evolution (Thornhill et al. 2019)—make an ideal genus to study plant genome evolution.

Continuing our study into plant genome evolution (Ferguson et al. 2023), we generated long-read sequences and assembled the genomes of 30 undomesticated *Eucalyptus* genomes and outgroups from two closely related genera, *Angophora floribunda* and *Corymbia maculata*. We create, combined with three previously and identically assembled *Eucalyptus* genomes (Ferguson et al. 2023), a data set covering about 1 million to 50 million years of diverging genome evolution, including all eight *Eucalyptus* subgenera (Thornhill et al. 2019; Nicolle 2022). Identifying all syntenic and rearranged regions between all species pairs, we show the rapid pace at which ancestral genome architecture is lost. We further analyzed our results to determine if ancestral genome architecture was being lost to sequence rearrangement, divergence beyond recognition, or insertions and deletions. Additionally, by framing synteny, rearrangement, and unaligned loss or gain with phylogenetic distance, we sought to describe the overall pattern of genome evolution.

## Results

### Sequencing and assembly

To investigate genome architecture, we performed nanopore long-read native DNA sequencing and de novo genome assembly for 32 Eucalypt species (30 *Eucalyptus*, one *Angophora*, and one *Corymbia*) (Table 1). All read libraries were trimmed and filtered in preparation of assembly. Curated read libraries had an average haploid coverage of 42.8× (range: 24.7× to 78.0×). For details of read libraries and sequence length distributions, see Supplemental Tables S1 and S2 and Supplemental Figures S1 and S2. *Eucalyptus pauciflora* FAST5 files were obtained from Wang et al. (2020), processed, and assembled as per our data sets, using a randomly selected 60× coverage of reads.

**Table 1.** Summary of de novo genome assembly, quality assessment, and annotation of 35 Eucalypt genomes

Species	Scaffolded genome size (Mbp)	% of genome in scaffolds	Scaffold N50 (Mbp)	Contig N50 (Mbp)	Contig count	BUSCO complete	LAI	TE %
*A. floribunda*	388.21	99.73%	36.02	4.02	224	96.82%	14.5	34.55%
*C. maculata*	403.82	99.90%	40.55	4.69	173	97.25%	15.92	36.26%
*E. albens* ^a^	606.89	99.79%	56.93	2.55	674	96.47%	17.3	46.57%
*E. ANBG9806169*	507.93	99.65%	49.61	2.40	476	96.86%	22.16	44.00%
*E. brandiana*	507.08	99.82%	45.47	7.28	168	98.11%	23.85	44.21%
*E. caleyi*	589.32	99.53%	59.52	4.77	276	96.47%	18.24	46.00%
*E. camaldulensis*	558.45	99.87%	52.65	2.48	418	96.73%	16.99	45.31%
*E. cladocalyx*	544.08	99.68%	51.92	2.80	390	97.59%	18.53	45.85%
*E. cloeziana*	480.07	99.75%	44.75	1.74	625	97.12%	19.06	42.57%
*E. coolabah*	606.31	99.53%	53.56	1.29	935	95.44%	15.9	45.89%
*E. curtisii*	435.26	99.96%	40.29	2.96	288	97.29%	18.34	41.66%
*E. dawsonii*	706.90	99.35%	67.73	0.99	1342	97.51%	17.01	45.88%
*E. decipiens*	590.95	99.50%	60.20	1.99	552	96.99%	18.87	46.95%
*E. erythrocorys*	539.20	99.99%	50.47	4.02	250	97.55%	20.18	47.07%
*E. fibrosa*	589.91	99.85%	55.66	6.45	192	96.73%	17.49	45.10%
*E. globulus*	545.02	99.28%	51.39	0.64	1747	96.69%	17.46	44.29%
*E. grandis*	615.89	99.44%	58.49	0.61	1747	96.09%	17.11	46.53%
*E. guilfoylei*	472.36	99.97%	44.61	4.25	209	98.02%	16.39	41.22%
*E. lansdowneana*	633.52	99.92%	59.67	2.35	489	97.12%	19.46	46.10%
*E. leucophloia*	568.48	99.38%	54.41	2.66	382	96.99%	17.91	44.37%
*E. marginata*	512.89	98.83%	50.56	1.01	989	96.17%	19.58	43.43%
*E. melliodora* ^a^	639.15	99.30%	60.83	1.87	564	98.67%	18.32	47.20%
*E. melliodora × E. sideroxylon*	603.57	99.80%	57.05	6.22	281	97.72%	17.96	46.71%
*E. microcorys*	440.91	99.92%	41.20	4.00	233	97.21%	16.2	41.39%
*E. paniculata*	588.85	99.66%	55.38	3.70	330	97.12%	18.58	44.92%
*E. pauciflora*	494.03	99.88%	50.46	6.58	209	97.25%	20.29	43.10%
*E. polyanthemos*	603.28	99.56%	57.46	4.66	300	96.82%	17.52	45.55%
*E. pumila*	529.75	99.70%	48.19	2.49	473	97.38%	17.74	44.17%
*E. regnans*	494.97	99.84%	47.06	5.26	205	97.25%	20.18	43.06%
*E. shirleyi*	597.18	99.88%	56.34	6.91	181	97.29%	19.89	45.85%
*E. sideroxylon* ^a^	592.133	99.87%	62.13	5.22	297	96.65%	18.68	46.57%
*E. tenuipes*	397.78	99.99%	35.74	3.43	207	96.39%	15.07	37.82%
*E. victrix*	557.16	99.85%	53.19	11.10	120	96.65%	18.71	44.34%
*E. viminalis*	558.71	99.11%	52.93	0.65	1755	96.47%	16.5	44.57%
*E. virginea*	532.79	99.97%	56.15	2.39	376	97.08%	17.69	43.78%

Alphabetically ordered list of genomes assembled and associated statistics.

^a^Genomes for *E. albens*, *E. melliodora*, and *E. sideroxylon* have been previously reported, being assembled using the same pipeline (Ferguson et al. 2023).

After assembling our trimmed and filtered read libraries, we curated our genomes, removing contigs identified as contamination and assembly artifacts. Additionally, we filtered haplotigs from our primary assemblies to form pseudohaploid genomes (Supplemental Table S3). Our genomes, which have a known and conserved haploid karyotype of 11 chromosomes (Ribeiro et al. 2016), assembled into an average of 517 contigs (range: 120–1755) (Table 1). At the completion of our assembly pipeline, our genomes had an average contig N50 of 3.65 Mbp (range: 614.30 kbp to 11.10 Mbp). Scaffolding contigs against *Eucalyptus grandis* (Myburg et al. 2014) greatly increased our genome contiguity, placing on average 99.69% of our genomes into pseudochromosomes (range: 98.83%–99.99%). We have found syntenic scaffolding within *Eucalyptus* to be suitable in the absences of chromosome conformation data (Ferguson et al. 2023) as Eucalypts have a conserved karyotype (Healey et al. 2021; Low et al. 2022). Additionally, within other genera, closely related genomes have been found suitable for scaffolding (Burns et al. 2021). Additionally, RaGOO provides confidence scores for assigning contigs to a scaffold, ordering contigs within scaffolds, and orienting contigs within scaffolds. Confidence scores achieved by our genomes indicated scaffolding was satisfactory (Supplemental Fig. S3).

The completeness of our genomes was evaluated with benchmarking universal single-copy orthologs (BUSCO) (Manni et al. 2021) and the long terminal repeat (LTR) assembly index (LAI) (Ou et al. 2018). A more complete genome will contain a high proportion of single-copy BUSCO genes, and all our genomes were found to be highly BUSCO complete (average: 97.01%; range: 95.44%–98.11). LAI searches a genome for LTR sequences and reports on the proportion that are intact. The LAI scores achieved by our genomes indicate that they are highly complete (average: 18.17; range: 14.50 to 23.85). Quality scores for all our genomes indicate that our genomes are of high quality, contiguity, and completeness (Table 1; Supplemental Table S4; for statistics and sequence distribution plots describing our genomes during and at the completion of assembly, see Supplemental Tables S5, S6; Supplemental Figs. S4–S6).

### Genome annotation

As masking of repeats within genomes aids in gene annotation, we annotated our genomes for both transposable elements (TEs) and simple repeats. Repeat annotation was performed using de novo repeat libraries built for each genome. Repeat annotation resulted in the classification of an average of 43.78% (range: 34.55%–47.07%) of our genomes as TEs and an average of 1.25% (range: 1.14%–1.39%) as simple repeats (Table 1; Supplemental Table S7). After soft-masking all genomes, we trained species-specific gene HMM models and subsequently annotated all genomes for genes. Gene models were trained on all available gene transcripts for *Arabidopsis thaliana* (taxonomy ID: 3702) and Myrtaceae (taxonomy ID: 3931) found within the NCBI (Sayers et al. 2021). Annotation predicted an average of 53,390 (range: 41,623 to 77,764) gene candidates within our genomes (Supplemental Table S8). Although the number of annotated genes is consistent with plant gene number estimates (Sterck et al. 2007), there is a wide variation between genomes. It is important to note the genes annotated within these genomes will contain both false positives and false negatives and are gene candidates, which in addition to real gene number variations will contribute to the variation in the number of annotated genes.

### *Eucalyptus* pangenome

**Figure 1.**
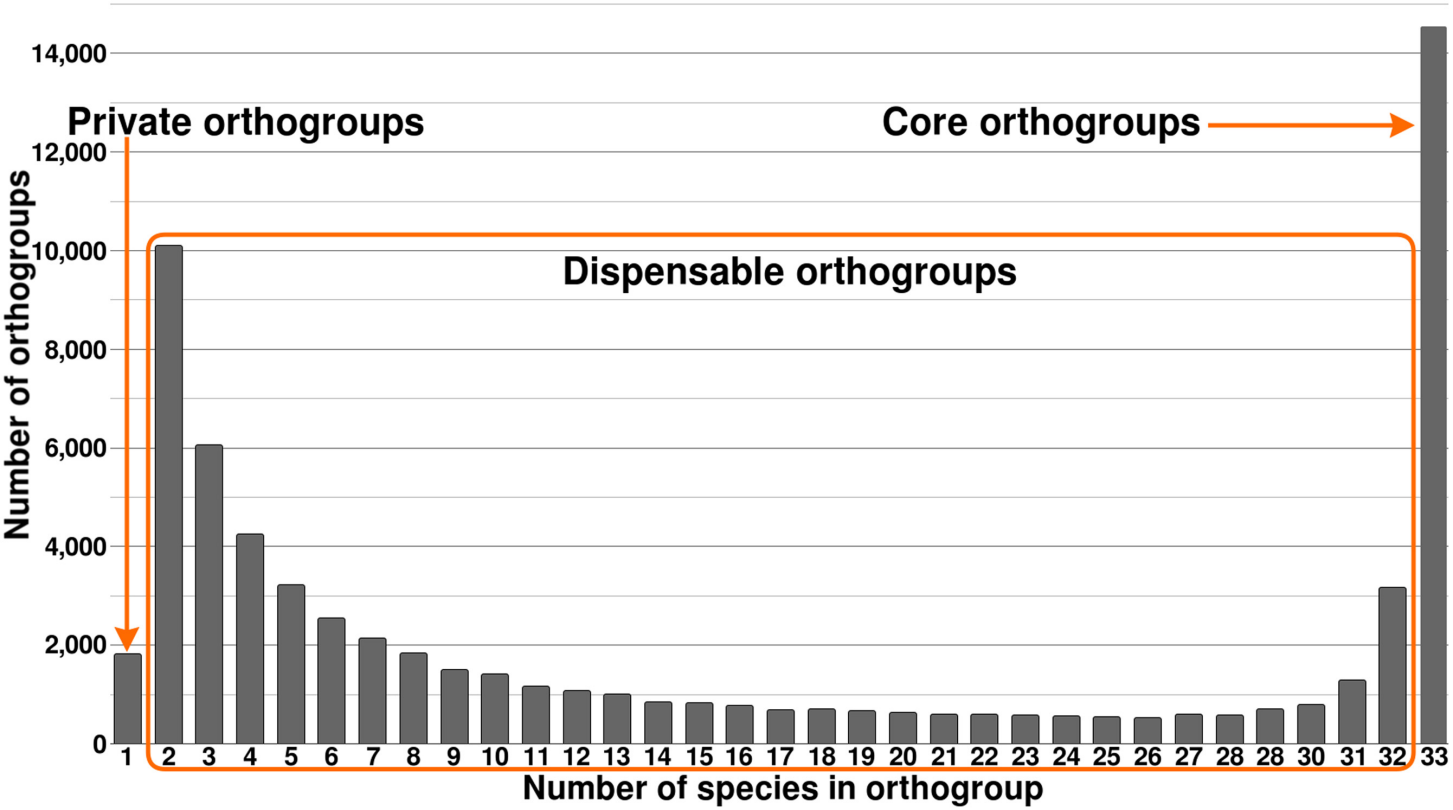
Pangenome of 33 species of *Eucalyptus.* Shows the number of orthogroups (OGs) shared by an increasing number of genomes. Private OGs are OGs that exist within a single genome, core OGs exist in all, and dispensable OGs are those that exist in two to 32 (n − 1) genomes.

Because of the shared evolutionary history of our genomes, many gene candidates will be homologs that have arisen prespeciation (orthologs) or postspeciation (paralogs) (Jensen 2001). To examine the evolutionary relationship between *Eucalyptus* gene candidates, we placed all highly similar primary (longest) gene transcripts into orthogroups (OGs). Of the 1,761,851 identified gene candidates across our 33 *Eucalyptus* genomes, 1,726,511 (97.99%) were placed into one of 68,248 OGs. The remaining 35,340 (2.01%) unique genes were not placed within an OG as their sequences were too dissimilar (>40% transcript identity and *e*-value < 0.001) to all other genes. On average, each genome had 98.03% (range: 94.62%–98.03%) of its gene candidates placed within an OG; 0.26% (4551) of all gene candidates were found to occur within a genome-specific OG. For detailed statistics on orthogrouping, see Supplemental Tables S8 and S9. Additionally, OGs were classified as core (present in all species), dispensable (present in at least two species), and private (present in a single species) (Fig. 1). A total of 21.33% (14,552) of the OGs were core, likely representing key *Eucalyptus* genes. Most OGs were dispensable, 76.00% (51,858), which may be a source of phenotypic and adaptive variation within the species. Only a very small number were private, 2.67% (1821), potentially representing highly species-specific genes and newly evolved genes.

### *Eucalyptus* phylogeny

**Figure 2.**
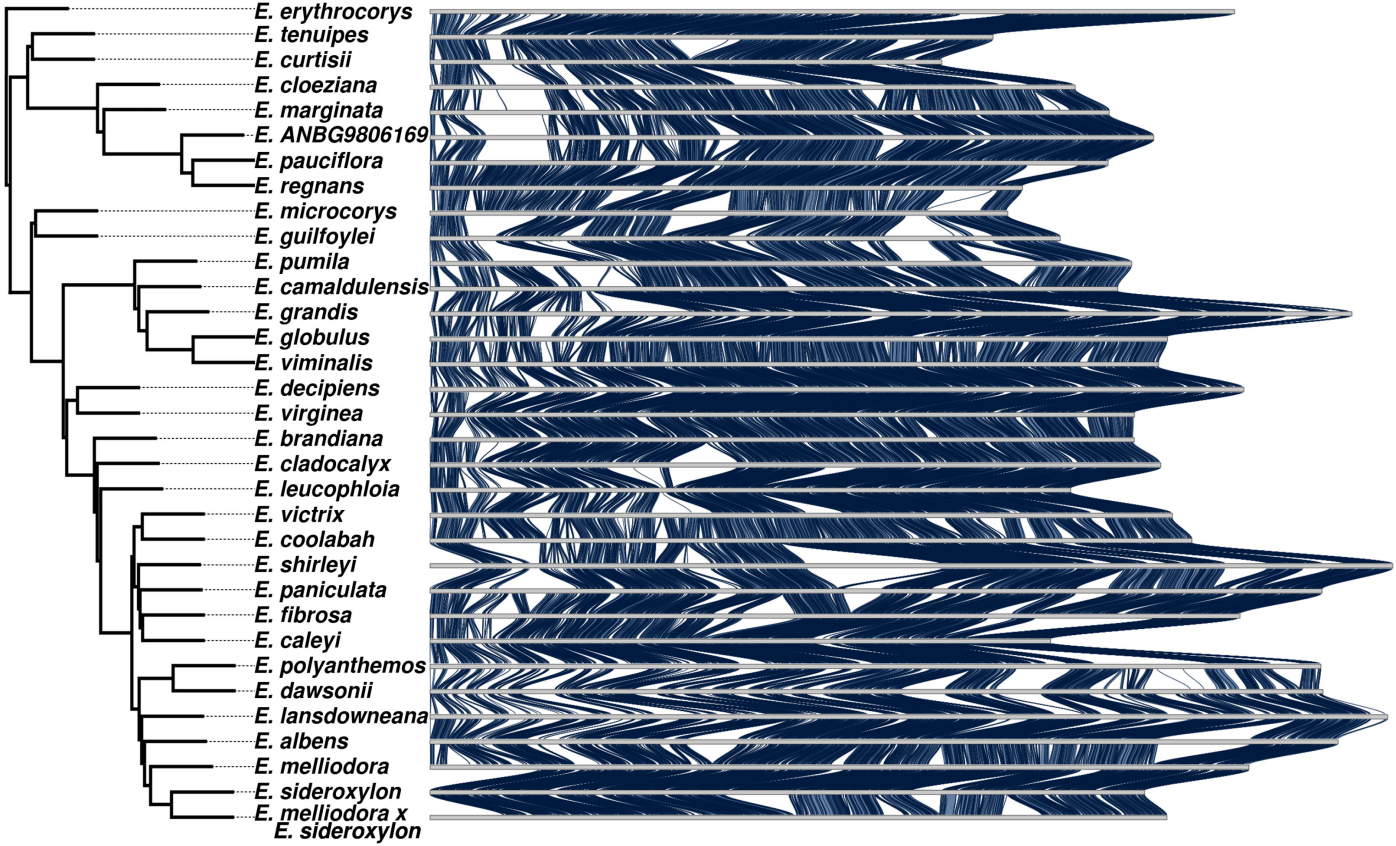
Synteny karyotype of Chromosome 1. Blue ribbons *between* karyotypes indicate the presence of syntenic sequences between species pairs. In all other regions, synteny has become lost. Synteny is lost to either rearrangements (inverted, translocated, or duplicated), sequence divergence, loss, or gain. Chromosomes are ordered by our phylogenetic tree.

To describe the evolutionary patterns between our genomes, we built a phylogenetic tree from single-copy BUSCO genes. We additionally included the *Corymbia calophylla* genome, which was identically assembled (Ahrens et al. 2021). From the initial BUSCO set of 2326 genes, we selected only genes present within 30 or more genomes, leaving 2106 BUSCO genes across our 36 genomes or 72,516 total genes. For each gene, we generated a multisequence alignment (MSA) with MAFFT, which we then trimmed and filtered, removing low-abundance regions and genes with overall poor alignments, leaving 1674 gene MSAs. Each MSA was used to construct a gene tree; subsequently, all gene trees were combined into a consensus species tree. The species tree was manually rooted using the established relationship between *Angophora*, *Corymbia*, and *Eucalyptus* (Fig. 2; Supplemental Fig. S7; Thornhill et al. 2019). The species tree in Newick format is available within the Supplemental Results.

After constructing the species tree, *Eucalyptus salubris* was unexpectedly found to be grouped with *E. pauciflora* and *Eucalyptus regnans*. If correctly placed, *E. salubris* would be a sister lineage to the *Adnataria* group (*Eucalyptus victrix* to *Eucalyptus sideroxylon*) (Thornhill et al. 2019). Morphological examination of the sample tree revealed that the tree was incorrectly labeled. The correct species name is currently unknown, as such we use its NCBI name, *Eucalyptus ANBG9806169*.

### Genome conservation and loss

To resolve the syntenic and nonsyntenic regions of our *Eucalyptus* genomes, we performed one-to-one genome comparisons for all genome pairs. Whole-genome alignments for all comparisons were analyzed with SyRI (Goel et al. 2019), and subsequently, all genomic regions within both genomes of an alignment pair were annotated as syntenic, rearranged (inversion, translocation, or duplication), or unaligned (sequence that only exists in one genome, resulting from either an insertion, deletion, or sequence divergence). As repeat masking would inflate the unaligned proportion of genome alignments and bias results, all genomes remained unmasked. This analysis resulted in all genomes being annotated for syntenic, rearranged, and unaligned regions 32 times, giving a total of 1056 annotated genomes. A visual summary of shared synteny was plotted using our phylogenetic ordering (see Fig. 2; Supplemental Figs. S8–S17). Inspection of synteny plots indicated that syntenic regions exist across the length of all chromosomes; however, synteny has become highly fragmented and is differently maintained. We acknowledge that large rearrangements (exceeding contig size) may be under- or overrepresented in our analysis owing to the current limitations of genome sequencing and scaffolding methods (see Discussion).

Next, we calculated the proportion of sequences shared between genomes, how sequences were shared (syntenic, inverted, translocated, and duplicated), and the frequency at which rearrangements occurred between genomes. For this analysis, we excluded all events <200 bp in length (the majority of which were small unaligned annotations). The majority of sequence was shared (syntenic and rearranged) between genomes, averaging 69.35% (range: 46.67%–91.86%). Only four pairwise alignments had <50% shared sequence: *Eucalyptus coolabah, Eucalyptus dawsonii*, *E. grandis*, and *Eucalyptus melliodora*, all compared with *Eucalyptus erythrocorys*. Synteny was the major contributor to shared sequence, averaging 39.32% (range: 21.34%–60.44%). Rearrangements averaged 30.24% (range: 16.97%–49.49%). The remainder of sequence was annotated as unaligned, averaging 30.43% (range: 8.08%–53.32%) (Table 2). For a per-species comparison breakdown of the percentage of genome shared, syntenic, rearranged, and unaligned, see Supplemental Tables S10 through S13.

**Table 2.** Summary of synteny, rearranged, and unaligned statistics of all pairwise genome analyses

	Average event size within each pairwise alignment (kbp)			Event counts			Percentage of genome		
Alignment type	Least	Most	Average	Least	Most	Average	Least	Most	Average
Syntenic	14.03	29.45	18.34	6657	18,810	12,153	21.34%	60.44%	39.32%
Unaligned	3.28	15.62	7.58	11,808	35,983	22,365	8.08%	53.32%	30.43%
Inverted	29.48	477.33	125.18	81	209	148	0.87%	10.46%	3.28%
Translocated	6.70	22.98	11.65	4322	15,975	9521	9.26%	41.14%	19.92%
Duplicated	2.48	6.14	3.60	3807	32,286	15,350	3.60%	38.97%	14.03%
Rearranged	—	—	—	8274	46,066	25,020	16.97%	49.49%	30.24%
Total shared	—	—	—	—	—	—	46.67%	91.86%	69.35%

Examination of the size and frequency of syntenic regions indicates that synteny between the 11 chromosomes of all genome pairs has, on average, fragmented into 12,153 (range: 6657 to 18,810) regions with an average size of 17.97 kbp (range: 16.27 kbp to 24.26 kbp) (for per genome average event size and frequency plots, see Supplemental Figs. S18, S19). Rearrangements in total (inversions + duplications + translocations) contributed more to synteny loss than did unaligned regions; however, unaligned contributed more than any single rearrangement type. A more detailed examination of the relative size and frequency of syntenic, rearrangement, and unaligned events showed that syntenic regions were long and common, unaligned regions were short and common, inversions were long and very rare, duplications were shortest and very common, and translocations occurred at a moderate frequency and size. Syntenic regions are distributed over the entire length of all chromosomes between all genome pairs; however, synteny has become highly fragmented by rearrangements and unaligned regions.

### Divergence time and genome conservation/loss

**Figure 3.**
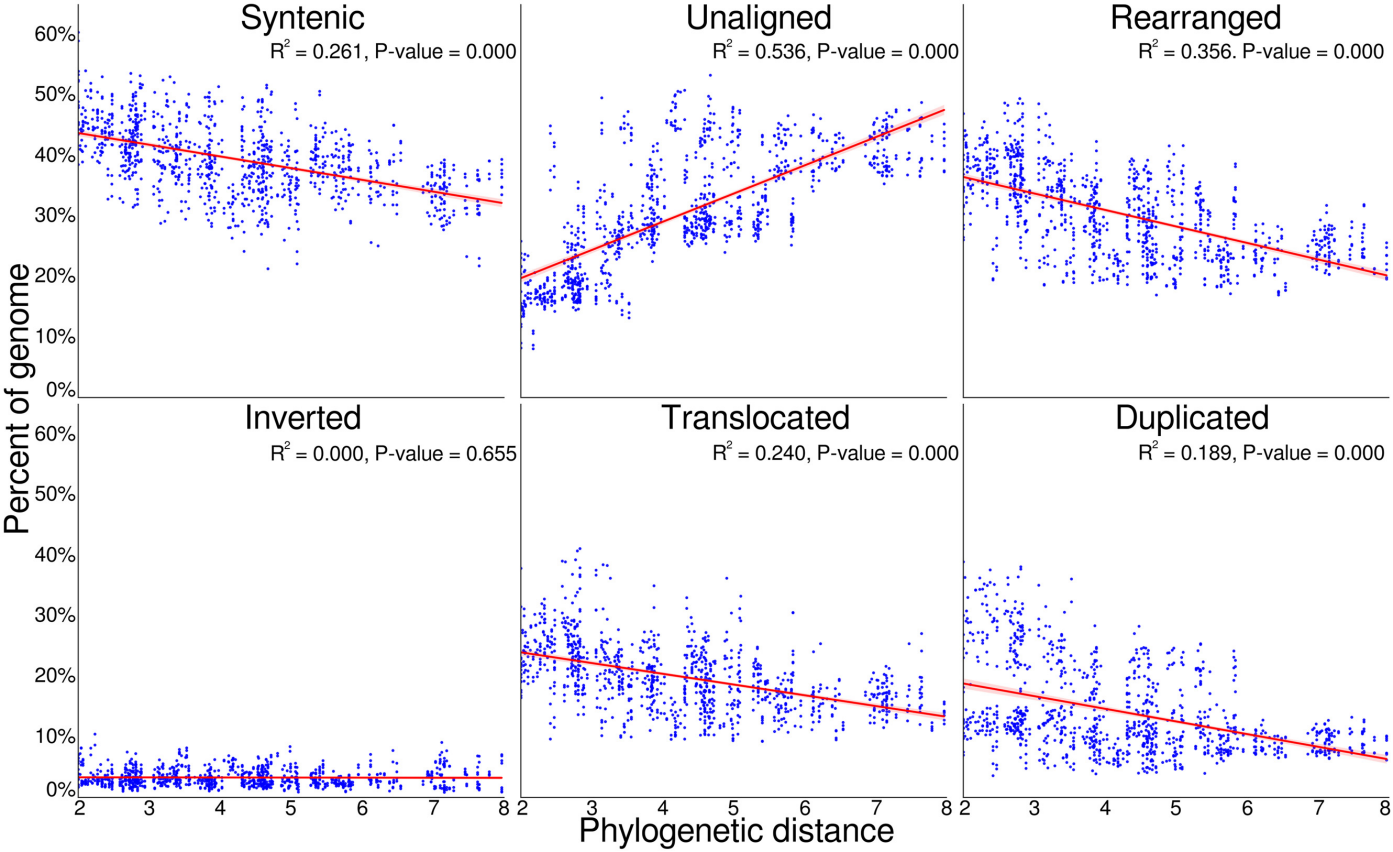
Pairwise genome conservation and loss, as phylogenetic distance increases. The proportion of both *Eucalyptus* genomes with an alignment pair that was identified as syntenic, rearranged, or unaligned, plotted against the phylogenetic distance of the two genomes. The unaligned proportion is the species-specific fraction of the genome between genome pairs, resulting from either an insertion, deletion, differential inheritance, or sequence divergence. When combined, the proportion of sequence that is syntenic, unaligned, and rearranged equals 100% for each genome within an alignment pair. The rearranged fraction is further broken down into inverted, translocated, and duplicated regions. Phylogenetic distance was calculated as the sum of branch lengths between each genome pair within phylogeny. *P*-value tests if the slope of the regression line is nonzero.

To examine these trends of architecture change over increasing divergence time, we examined the relationship between phylogenetic distance and genome conservation and divergence (Fig. 3). We find that as phylogenetic distances increase, the proportion of syntenic (R^2^ = 0.261) and rearranged (R^2^ = 0.356) sequence decreased as lineages acquire unique genomic variation. Similarly, as phylogenetic distances increase, the proportion of genomes within duplications (R^2^ = 0.189) and translocations (R^2^ = 0.240) decreased, whereas the portion of genomes unaligned quickly increases with increasing phylogenetic distance (R^2^ = 0.536). Inversions consistently occupied a small proportion of genomes across all phylogenetic distances (R^2^ = 0.000).

Unaligned sequences accumulate through the loss, gain, or divergence of sequences. As genome sizes are similar (average: 552.75 Mbp; standard deviation: 65.62 Mbp), sequence loss and gain are unlikely to fully explain the rapid accumulation of unaligned sequences. Divergence beyond recognition is likely the largest contributing factor. To test which regions were contributing to the growth of unaligned sequences, we gathered all alignment identity scores for all syntenic, inverted, translocated, and duplicated regions, in each pairwise alignment. Plotting identities against phylogenetic distance, we examined the rate at which sequences diverge. Syntenic was observed to lose sequence homology more rapidly (R^2^ = 0.516) compared with duplicated (R^2^ = 0.236), translocated (R^2^ = 0.303), and inverted (R^2^ = 0.260) (Supplemental Figure S20). However, in all cases the regression spanned a very small interval (syntenic: 91.58%–93.14%; duplicated: 91.48%–92.63%; translocated: 91.56%–92.81%; and inverted: 91.49%–92.72%), and none approached our 80% sequence similarity threshold for alignments.

Overall, we find that the syntenic proportion of the genome decreases slowly with increasing divergence time, whereas the proportion rearranged as duplications and translocations decreases faster. The loss of homology between synteny, duplicated, and translocated regions leads to a strong increase in the unaligned portion of the genome (insertions, deletions, and diverged sequences) as divergence time increases. The loss of duplications and translocations contribute more to the growth of unaligned than does synteny. We benchmarked our scaffolding with one species using Hi-C and found consistent results, with a limitation on the number of inversions, which may be underreported, and translocations, which may contribute less to ongoing genome divergence than reported (Supplemental Results).

### Genome-specific and group-wide sequences

**Figure 4.**
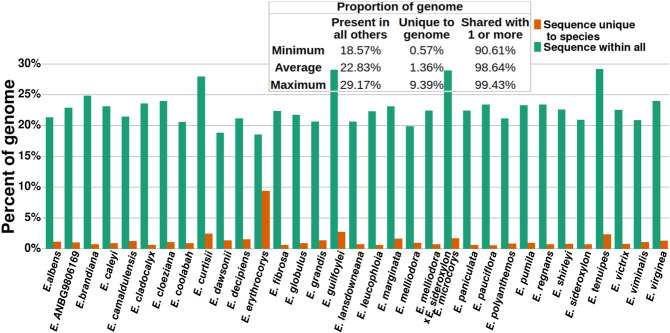
Proportion of *Eucalyptus* genomes unique and shared by all others. Sequence unique to species is the union of the genome that was classified as unaligned within all pairwise alignments. Sequence within all is the union of the genome that was classified syntenic and rearranged (i.e., common between genomes) within all pairwise alignments.

Unaligned sequences occupied on average 30.65% of each *Eucalyptus* genome within each pairwise alignment. To determine if these sequences were unique to a single genome or were shared between multiple, all pairwise alignments for each species were combined and the number of species sharing each base calculated. Subsequently, genome regions that were unique to a genome, shared by multiple genomes, or shared by all genomes were identified (Fig. 4).

Genome-specific (unique) sequences occupied an average of 1.36% (241.55 Mbp) of the 33 *Eucalyptus* genomes; the remaining 98.64% of sequence was shared by one or more genomes. The proportion of each genome shared by all others averaged 22.83%. This finding mirrors our OG analysis in which 2.67% of groups were private, 76.00% dispensable, and 21.33% were core.

Of note is *E. erythrocorys*, whose genome had a significantly lower proportion of genome-specific sequence and a higher proportion of sequence shared by all other genomes. *E. erythrocorys* is the sister taxon of all our other genomes within our *Eucalyptus* data set. Given the age of the divergence between the *E. erythrocorys* lineage and its sister lineage, this genome was expected to display a unique pattern in this analysis; however, the extent to which *E. erythrocorys* is different from all others was surprising.

### Lineage-conserved rearrangements

**Figure 5.**
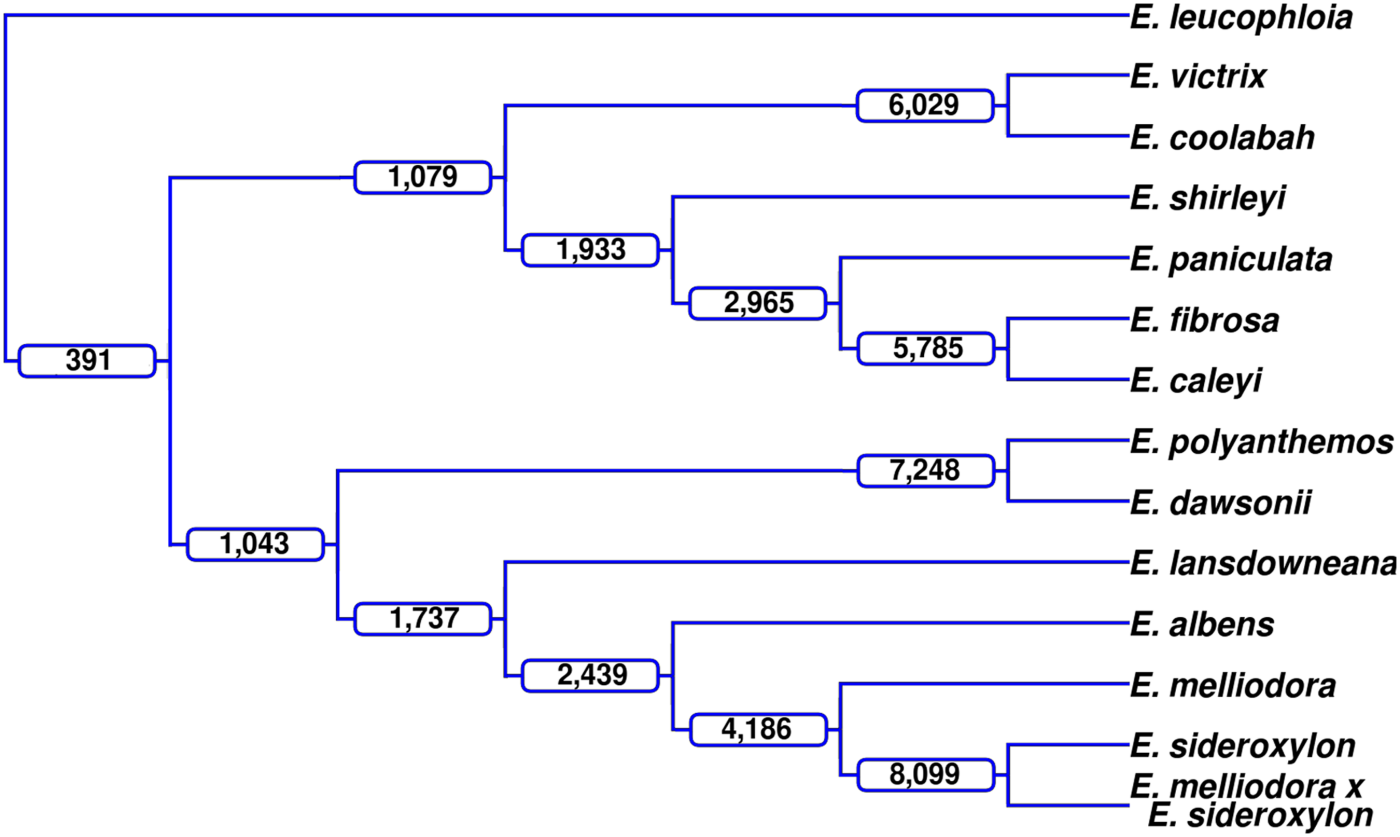
Lineage-conserved rearrangements. Using an outgroup genome, *Eucalyptus leucophloia*, rearrangements were identified that were shared among members of a lineage, that is, rearrangements with the same start and end points within the outgroup genome. At each branch in the dendrogram, the number of rearrangements shared by all taxa within that clade are labeled; for example, *E. victrix* and *E. coolabah* share 6029 rearrangements.

The one-to-one analysis of our *Eucalyptus* genomes has described a genome structure that has become highly fragmented by frequently occurring rearrangements and unaligned regions. As genome structure is inherited by offspring, some of the rearrangements discovered during our analysis are assumed to exist within the genomes of monophyletic groups, namely, a group of species that have descended from a single ancestral species. Rearrangements found within multiple genomes also help to confirm their validity. To search for evidence of inherited rearrangements, we analyzed the *Adnataria* section, for which we have the best coverage of genomes (13 genomes) (see Fig. 5). Additionally, using only *Adnataria* genomes should maximize the occurrence of retained rearrangements, as the phylogenetic distances within the *Adnataria* group are relatively low, with many species still hybridizing (Delaporte et al. 2001).

As all alignments and subsequent annotations are relative to the two species involved, directly comparing the breakpoints of annotations to find common rearrangements is not possible. Therefore, an outgroup genome, *Eucalyptus leucophloia*, a sister species of the *Adnataria* group, is used for comparison with each of the 13 selected genomes. The outgroup genome imposes a single set of genetic coordinates and genome architecture, enabling comparisons of rearrangement breakpoints and subsequent identification of shared rearrangements. Shared rearrangements will contain the same sequence. Although this method allows us to find common inversions, translocations, and duplications, it does not allow us to find unaligned (insertions, deletions, and highly diverged) regions between our ingroup genomes, as genomes are not being directly compared.

Comparing the start and end breakpoints (±50 bp) for events >1 kbp (250,693 total rearrangements across all *Adnataria* genomes) identified 58,388 (23.29%) common rearrangements (rearrangements that exist within two or more genomes). Of the 58,388 common rearrangements, 28,059 (48.06%) were shared by two genomes, and 391 (0.67%) were shared by all. The number of common rearrangements quickly decreased as the number of genomes increased (Supplemental Fig. S21). Lineage-conserved rearrangements were identified by tracing common rearrangements through *Adnataria's* phylogeny (Fig. 5). As expected, more closely related genomes shared the largest number of rearrangements, whereas more distant genomes shared less. Additionally, as the number of descendant genomes of nodes increased, the number of shared rearrangements also decreased. Inherited rearrangements were identified within the *Adnataria* group. We repeated this analysis twice using *Eucalyptus brandiana* and *Eucalyptus cladocalyx* as the outgroup genome achieving similar results (Supplemental Figs. S22, S23).

### Gene content of synteny, rearrangements, and unaligned events

To assess whether rearrangements that encompass genes are selected against, we calculated the proportion of genic (contains a gene/s) and nongenic (contains no gene/s) rearrangements, as well as syntenic and unaligned events per genome. Initially, all events too small to contain a gene and genes unplaced within an OG were removed. A conservative event length of 1 kbp was used to filter out events, as events smaller than this are unlikely to contain a gene (Xu et al. 2006). Genes unplaced within an OG are highly dissimilar to all other gene candidates and may be false positives resulting from incorrect annotation. The remaining rearrangement, synteny, and unaligned events were examined for the presence of genes placed within an OG and subsequently classed as genic or nongenic.

**Figure 6.**
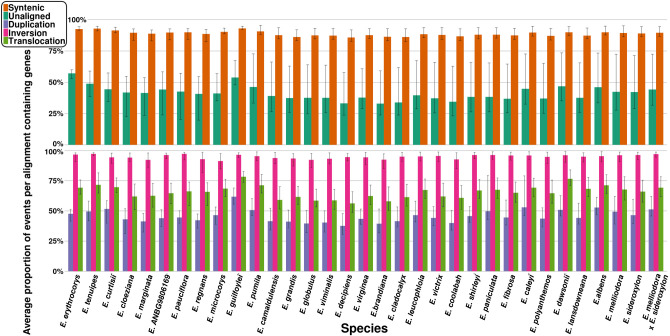
Average proportion of genic events for each species genome. The proportion of genic events was calculated for each pairwise alignment and was averaged. Error bars indicate the minimum and maximum proportion of genic events found when aligned to all other genomes.

For each genome, compared with all other genomes, we calculated the average proportion of genic syntenic, inverted, translocated, duplicated, and unaligned events and plotted the results (Fig. 6). An average of 88.80% (range: 82.52%–95.57%) genic syntenic events were observed across our genomes. Genic unaligned averaged 41.13% (range: 19.76%–73.48%), genic inversions averaged 94.93% (range: 81.65%–99.13%), genic translocations averaged 65.70% (range: 48.77%–83.98%), and genic duplications averaged 45.71% (range: 30.59%–79.20%).

Additionally, we analyzed the effects of divergence time on the proportion of genic events for all rearrangement types, synteny, and unaligned (Supplemental Fig. S24). Results indicated that phylogenetic distance has little to no impact on the proportion of genic syntenic events (R^2^ = 0.162), inverted events (R^2^ = 0.000), translocation events (R^2^ = 0.004), or duplication events (R^2^ = 0.002). Unaligned was the only event type whose genic proportion was affected by phylogenetic distance. As phylogenetic distance increases, unaligned events become more genic (R^2^ = 0.292; *P*-value = 0.000).

## Discussion

In this study, we created a large collection of wild and naturally evolving high-quality *Eucalyptus* genomes covering 1 million to 50 million years of divergent evolution. Using these genomes, we find a pattern of genome evolution led by an initial rapid accumulation of rearrangements and subsequently a slow loss of both rearranged and syntenic sequences as lineage-specific mutations erode sequence homology. Rearrangements, likely because of their recombination effects and subsequent fixation/reduction of alleles (Faria and Navarro 2010), are lost more rapidly than are syntenic regions. Translocations and duplications were the major disruptors of synteny and were rapidly lost as divergence times increased. Inversions did not contribute substantially to the loss of synteny or the loss of rearrangements, instead occurring at consistently low rates across all divergence times. As genome sizes remain constant and little species-specific sequence exists across our data set, loss of existing sequence or gain of new sequence provides an unlikely explanation for the growth of unaligned sequences as divergence times increase. Hi-C results provided confidence that our scaffolding method was not highly influencing our conclusions. These results showed that the translocations’ initial contribution to genome divergence was significant; however, they do not significantly contribute to ongoing genome divergence. But as this assessment was an almost worst-case scenario for our scaffolding, translocations are still likely a significant contributor to ongoing genome divergence.

Duplications are a major contributor to functional and genome divergence (Lynch and Conery 2000; Adams and Wendel 2005) especially within plant lineages (Hanada et al. 2008; Van de Peer et al. 2009). We found that duplications were highly abundant between all genomes, and that at smaller divergence times, duplications contributed strongly to genome divergence. As the time since divergence increased, the contribution of duplications to genome divergence lessened, becoming overshadowed by unaligned portions of the genome. However, at all phylogenetic distances, duplications were a major contributor to genome divergence (occupying on average 14.03% of genomes across an average of 15,350 events). The observed pattern of duplication loss as the time since divergence increased was unsurprising, as duplications, although highly important to adaptation and evolution, are rarely conserved (Inoue et al. 2015; Naseeb et al. 2017). Why some duplications are preserved while the majority are lost is speculative; however, theory centers on neofunctionalization, subfunctionalization, and novel function evolution (Freeling et al. 2015; Braasch et al. 2016; Lien et al. 2016; Wu and Cox 2019). These hypotheses rely upon the genic properties of duplications; namely, if duplications do not gain novel function or retain ancestral function, purifying selection will likely result in their removal (Wu and Cox 2019). Although duplications were the least genic of all rearrangement types, a significant number (45.71%) were found to contain genes, likely contributing to their preservation. Nongenic duplications, being less visible to selection, are likely to experience increased evolutionary rates (mutations) and genetic drift (Scannell and Wolfe 2008), eventually mutating beyond recognition and ultimately contributing to the unaligned proportion of alignments. Duplications, both preserved and unpreserved, are likely one of the greatest sources of genome divergence.

Chromosomal inversions, which are known to be associated with the development of complex phenotypes, local adaptation, and speciation (Lowry and Willis 2010; Twyford and Friedman 2015; Arostegui et al. 2019), were extremely rare between all genomes (average: 148 between genomes) and contributed less than all other types of rearrangement to genome divergence. This observation was consistent at all phylogenetic distances: As the time since divergence increased, the number of inversions remained constant. A similar finding was made by Hirabayashi and Owens (2023). Inversions likely occur at a high rate within plant genomes (Huang and Rieseberg 2020); however, a low number of inversions was identified, suggesting that inversions are strongly selected against and rarely maintained. To survive underdominant selection, a novel inversion must provide enough selective advantages to outweigh its disadvantages. Inversions may provide a selective advantage by rearranging recombination loci and by linking alleles captured within their bounds. Inversion-linked alleles can be strongly selected for, if adaptive, and rise to high frequencies within populations (Rieseberg 2001; Harringmeyer and Hoekstra 2022). Additionally, adaptive alleles linked by inversions can be protected from strong gene flow (Yeaman 2013). Alternatively, inversions may instead hinder adaptation. If selective conditions were to alter, previously adaptive inversions could prevent recombination from producing new allele combinations suitable for the new conditions (Rieseberg 2001). Inversions, because of recombination suppression, also reduce effective population size and increase genetic load, as purifying selection cannot purge linked deleterious mutations (Jay et al. 2021). The inversions identified here, which are assumed to have survived selection, were all very large, as expected (Wellenreuther and Bernatchez 2018), with the majority (94.93%) containing genes. Inversions are rare and contribute little to genome divergence but are highly genic and likely play a significant role in adaptation, evolution, and speciation processes.

Translocations can have similar genomic effects to inversions (Ortiz-Barrientos et al. 2016), contributing to the development of complex phenotypes, local adaptation, and speciation by disrupting recombination (Martin et al. 2020). As for inversions, novel translocations that survive drift must provide enough selective advantages to outweigh their disadvantages or be removed by purifying or underdominant selection. Translocations were highly abundant between recently diverged, phylogenetically close genomes. As the time since divergence increased, translocations reduced in frequency but remained common. Translocations were the most common type of large rearrangement (average size: 11.2 kbp), mirroring results obtained by Martin et al. (2020). Translocations were much more abundant than inversions, especially when genomes have recently diverged, suggesting that translocations are less strongly selected against than inversions, despite having a similar effect on recombination. Additionally, translocations, although highly genic (65.70%), were less genic than inversions (94.93%). The different genomic pattern observed for translocations and inversions is possibly owing to the effects of local versus nonlocal changes to recombination. Meiotic recombination may be more disrupted when reordered recombination loci are close to their location of origin. If true, purifying selection acts more strongly on inversions than on translocations. Translocations are common and, along with duplications, are a major contributor to genome divergence, possibly aiding in adaptation, evolution, and speciation processes. However, as the effects and mechanisms of translocations have been less studied than other rearrangements (Robberecht et al. 2013), it remains to be seen if they are more likely to have functional/adaptive significance.

Although new long-read sequencing technologies have accelerated studies on genome structural variations, identifying structural variations still presents challenges. Here, we used RaGOO (Alonge et al. 2019) for reference-guided scaffolding of our mega-base-pair-sized contigs into chromosomes, as obtaining Hi-C data from recalcitrant *Eucalyptus* tissue with high oil content is challenging. We assert that this approach is most suitable given data set limitations and the well-conserved genome organization observed in the *Eucalyptus* genus (Potts and Wiltshire 1997; Booth et al. 2015; Grattapaglia et al. 2015; Butler et al. 2017; Supple et al. 2018), as well as in closely related genera within the Myrtaceae family, including Corymbia (Healey et al. 2021), Melaluca (Voelker et al. 2021; Chen et al. 2022), and Syzygium (Low et al. 2022; Ouadi et al. 2022). This simplifies reference-guided scaffolding, unlike genera with variable karyotypes, ploidy, and intentional introgressions, such as Solanum (Alonge et al. 2019; Razifard et al. 2020). However, reference-guided scaffolding may underrepresent macroscale inversions (those larger than contig lengths), as observed when comparing results obtained with reference-scaffolded and Hi-C-scaffolded genome assemblies of *E. melliodora*. Despite this limitation, our primary results and conclusions remain unaffected; syntenic and rearrangements were contained within contigs that are orders of magnitude longer (Tables 1, 2). In *Eucalyptus*, we found inversions to be rare, ∼1% of all structural variations (Table 2), consistent with studies across diverse plant genera (Hirabayashi and Owens 2023) and highly domesticated crop plants like maize (Hufford et al. 2021). Obtaining Hi-C data in more species may help resolve large-scale inversions, although they can introduce errors (Alonge et al. 2019) and still represent prediction and hypothesis. An alternative strategy is single-cell/single-strand genome sequencing (Falconer et al. 2012), which was found to be one of the most reliable methods to detect large-scale inversions in human genomes (Chaisson et al. 2019). As long-read sequencing technologies advance, the assembly of telomere-to-telomere genomes, independent of Hi-C data and genome scaffolding, will greatly enhance genome studies and overcome technical challenges in structural variation discovery. These advances are exemplified by long-read de novo assemblers such as hifiasm (UL) (Cheng et al. 2023) and Verkko (Rautiainen et al. 2023).

To further investigate the potential importance of the syntenic, rearranged, and unaligned genome regions identified in our study, further research using genome-wide association studies (GWAS) of phenotypes measured on seedlings in pots or field trials, as well as landscape and genome-wide genotyping for genome–environment association (GEA) scans for adaptive rearrangements, are needed. Within-species-derived rearrangements are predicted to be predominantly neutral and exist at low frequencies, whereas others rising to higher frequencies could be true lineage-specific adaptive rearrangements. With additional genomes from populations, the frequency of rearrangements within each species could be assessed. This would provide insight into the functional significance of the widespread genomic rearrangements we have found and would potentially identify rearrangements conferring adaptive traits across the landscape.

*Eucalyptus* contains more than 800 species that exist across a wide geographic and environmental range, while retaining a largely conserved karyotype (Potts and Wiltshire 1997; Booth et al. 2015; Grattapaglia et al. 2015; Butler et al. 2017; Supple et al. 2018), which makes the genus ideal to study plant genome evolution. Here we assembled representative genomes of 33 species, creating one of the most comprehensive data sets to study plant genome evolution. These genomes provide a genus-wide resource to study genome rearrangements, and they support future *Eucalyptus* research that require genomic references. Our findings suggest that following divergence, genome architecture is highly fragmented, predominantly by rearrangements. As genomes continue to diverge, genome architecture continues to be slowly lost. Additionally, as genomes diverge, they increasingly become unalignable owing to the divergence of duplications and translocations. Syntenic regions also contribute to the growing unalignable proportion of genomes, but at a slower rate than that of rearrangements. Duplications and translocations are potentially the greatest contributors to functional and genome divergence, aiding in the development of complex phenotypes, and local adaptation. Inversions occur at consistently low rates, contributing little to genome architecture loss or accumulation of unalignable sequences. However, inversions were highly genic, much more so than either duplications or translocations, and likely also play a crucial role in the development of complex phenotypes and in local adaptation. Genome architecture results from a complex interaction of positive, neutral, and negative forces, all of which contribute to the evolution, divergence, and adaptability of species (Koonin 2009; Huang and Rieseberg 2020; Mérot et al. 2020). However, owing to technical limitations, the evolution of genome architecture and its role within biology is not well understood (Lynch et al. 2011; Cortés et al. 2018; Jiggins 2019). Here, by describing the pattern of genome architecture as time since divergence increases of 33 *Eucalyptus* genomes, we contribute to a better understanding of the evolution of plant genomes. Rearrangements, along with polyploidy, TEs, and other genome evolutionary mechanisms, play an important role in plant genome evolution (Galindo-González et al. 2017; Marques et al. 2019; Meudt et al. 2021). Further research in other plant lineages is required to assess the prominence of rearrangements upon genome evolution.

## Methods

### Sampling

*Eucalyptus* species used in this study were collected throughout multiple locations in Australia, which are detailed in the Supplemental Results. The majority of collected species are living collections with accession numbers at the Australian National Botanic Gardens (Canberra, Australian Capital Territory [ACT]) and Currency Creek Arboretum (Currency Creek, South Australia). Additional samples were sourced from the Australian National University (Acton, ACT), from the National Arboretum Canberra (Molonglo Valley, ACT), from the University of Tasmania Herbarium (Sandy Bay, Tasmania), and within *Eucalyptus* woodlands of southern Tasmania. Leaves were placed in plastic zip-lock bags, lightly sprayed with water to keep them moist, and transported to the laboratory as soon as possible, where they were washed with water and stored at −80°C until DNA extraction.

### DNA extraction, sequencing, and basecalling

To extract high-molecular-weight DNA from recalcitrant *Eucalyptus* samples, we developed two methods. Initially we combined a protocol to purify nuclei with hexylene glycol (Bolger et al. 2014) with a magnetic bead-based DNA extraction protocol (Mayjonade et al. 2016), which was further developed and is available on Protocols.io in detail (Jones and Borevitz 2019). This was further optimized and developed, which led to the second method of adopting a sorbitol prewash of homogenate (Inglis et al. 2018) to wash crude nuclei instead of isolating pure nuclei, followed by a magnetic bead–based DNA extraction, according to a method previously described (Jones et al. 2021). We found this method to be more time and resource efficient; hence, we switched to this method for all subsequent high-molecular-weight DNA extractions. For each *Eucalyptus* sample, the method that was used is listed within the Supplemental Material, Supplemental Table S1, with the two methods being referred to as nuclei and sorbitol, respectively.

After isolating high-molecular-weight DNA, we further purified and size-selected the DNA by using a PippinHT (Sage Science). The DNA was size-selected for fragments ≥20 kb or ≥40 kb depending on DNA yield and molecular weight, which are listed in the Supplemental Material, Supplemental Table S1, for each sample. Two Oxford Nanopore Technologies (ONT) long-read native DNA sequencing libraries were prepared for each species according to the manufacturer's protocol 1D genomic DNA by ligation (SQK-LSK109). *Eucalyptus marginata* was an exception, which had one ligation library as described, but the second was a transposome library prep, according to the manufacturer's protocol for rapid sequencing (SQK-RAD004). Sequencing was performed on MinION Mk1B devices using two FLO-MIN106D R9.4.1 flow cells per species. Sequencing output was improved when ONT flow cell wash kits (EXP-WSH003 and EXP-WSH004) were made available, whereby flow cells were washed when sequencing declined and were primed again, and more library was loaded, according to the manufacturer's instructions. After sequencing was complete, the FAST5 reads were basecalled with ONT Guppy (versions 3.3.0, 4.0.11, 4.0.14, and 4.0.15) (for per species versions, see Supplemental Results).

We complemented the long-read sequencing with highly accurate Illumina short-read sequencing for later use in genome polishing of the long-read de novo assemblies. Illumina short-read, whole-genome DNA sequencing libraries were generated using a cost-optimized, transposome protocol based on Illumina Nextera DNA prep methods (Jones et al. 2023). The pooled libraries were then size-selected for fragments with insert sizes between 350 and 600 bp with a PippinHT (Sage Science). Multiplexed sequencing with other projects was performed on a NovaSeq 6000 (Illumina), using a lane of an S4 flow cell with a 300-cycle kit (150-bp paired-end sequencing), at the Biomolecular Resource Facility, Australian National University, ACT, Australia.

### De novo assembly

De novo assembly and annotation were performed using the long-read de novo plant assembly protocol developed by Ferguson et al. (2022). Briefly, FASTQ reads are quality-screened, removing DNA control strand, sequencing adaptors, low-quality read ends (the first and last 200 bp), short reads (>1 kbp in length), and low-quality reads (average quality < Q7), using the NanoPack set of tools (De Coster et al. 2018). Curated reads are next assembled using the long-read assembler Canu (versions 1.9 and 2.0) (Koren et al. 2017), which assembles high-quality *Eucalyptus* genomes (Ferguson et al. 2023). Assemblies were filtered of contamination (nonplant contigs), assembly artifact, plasmid, and haplotig contigs (contigs that span the same genomic region but originate from different parental chromosomes) using BlobTools (Laetsch and Blaxter 2017) and Purge Haplotigs (version 1.1.0) (Roach et al. 2018). Next, all assemblies were long-read and then short-read polished, using assembly reads and Illumina reads originating from the same individual as used for assembly. Long-read polishing was performed with Racon (Vaser et al. 2017); short-read, with Pilon (version 1.3.1) (Walker et al. 2014). Long-read polishing made use of the long-read aligner minimap2 (version 2.17) (Li 2018), whereas short-read polishing used BWA-MEM (version 0.7.17) (Li 2013). Next, assemblies were filtered to remove all contigs <1 kbp in length. We chose this contig length threshold so as to maximize genome contiguity while removing all contigs too small to contain a gene. Finally, assemblies were scaffolded using homology with *E. grandis* (Myburg et al. 2014). Scaffolding was performed with RaGOO (version 1.1) (Alonge et al. 2019) and minimap2.

After assembly, all genomes were quality-assessed using BUSCO (version 5; database: eudicots_odb10.2020-09-10) (Manni et al. 2021), LAI (version 2.9.0) (Ou et al. 2018), and assembly statistics.

### Transposon and gene annotation, and gene orthogrouping

Genome repeat and gene annotation was also performed using the long-read de novo plant assembly protocol developed by Ferguson et al. (2022). First, de novo repeat libraries were created for each genome using EDTA (version 1.9.6) (Ou et al. 2019); subsequently, all genomes were repeat-annotated with RepeatMasker (version 4.0.9) (Smit et al. 2020). All genomes were repeat-soft-masked and subsequently annotated for genes. Gene annotation was performed with BRAKER (version 2.1.5) (Brůna et al. 2021) using GeneMark-EP (version 4) (Brůna et al. 2020). Gene transcript sequences for model training were obtained from the National Center for Biotechnology Information (NCBI) (Sayers et al. 2021). Included in gene training data were all Myrtaceae (taxonomy ID: 3931) and *A. thaliana* (taxonomy ID: 3702) transcripts. All gene candidates were grouped into OGs using OrthoFinder (version 2.5.4) (Emms and Kelly 2019). Using DIAMOND (Buchfink et al. 2021), OrthoFinder aligned all gene transcripts, grouping those with >40% identity and achieving an *e*-score < 0.001.

### Genome synteny, rearrangement, and unaligned annotation

Identification of all shared sequences began by aligning all pairwise combinations of genomes with the MUMmer (version 3) (Kurtz et al. 2004) tool NUCmer (parameters: ‐‐maxmatch -l 40 -b 500 -c 200). NUCmer first identifies all shared 40-mers between genomes and their locations. Next, 40-mers within 500 bp are clustered, creating a list of collinear blocks or alignments. Last, using MUMmer's delta-filter tool, alignments are filtered, removing all alignments <200 bp in length and <80% similar. A low 80% alignment similarity score was used as *Eucalyptus* are highly heterozygous (Murray et al. 2019), and a more stringent similarity score may incorrectly filter out real alignments.

Having identified all shared sequences, we next annotated all syntenic, rearranged (inverted, translocated, and duplicated), and unaligned (sequence that only exists in one genome, resulting from either an insertion, a deletion, or a sequence divergence) sequences between pairwise genomes using SyRI (version 1.5) (Goel et al. 2019). SyRI's use of a directed acyclic graph results in genomes being annotated for smaller regions, which, when occurring in an unbroken series of a single type, get combined. The resulting output includes both levels of annotations: smaller and more fragmented, and larger and more contiguous. We make use of the larger and more-continuous alignments. Additionally, we combined inverted duplications with duplications, as well as inverted translocations with translocations.

### Phylogeny

Using highly conserved and single-copy BUSCO genes, we built a eucalypt phylogenetic tree describing the evolutionary relationships between all genomes included in this study. The phylogenetic tree included four previously and identically assembled genomes for *Eucalyptus albens*, *E. melliodora*, *E. sideroxylon*, and *C. calophylla*, creating a data set of 36 genomes. To begin, FASTA sequences for all single-copy BUSCO genes found within 30+ genomes were collected. Using masce (version 2.03) (Ranwez et al. 2018), MSA was performed individually on all genes. As errors within gene MSAs will subsequently lead to errors in phylogenetic inferences, we trimmed and filtered all gene MSAs. Gene sequence errors were detected and removed using HmmCleaner version 0.180750; (Di Franco et al. 2019). HmmCleaner uses a profile-hidden Markov model to identify sequence segments that poorly fit the gene MSA and subsequently removes them. Errors resulting from poor alignments were removed using report2AA (parameters: -min_NT_to_keep_seq 30, -min_seq_to_keep_site 4,-dist_isolate_AA 3, -min_homology_to_keep_seq 0.5, -min_percent_NT_at_ends 0.7) from the macse program. report2AA removed sites within MSAs that included fewer than 30 genomes, had fewer than four informative characters, or had isolated sites (site was more than three characters away from the next nongap character). Additionally, report2AA removed genomes from MSAs that had <50% homology with another genome within the MSA, and trimmed both MSA ends that had <70% of aligned sites as nucleotides (i.e., 26+ genomes had to have a nongap character). Additionally, as a result of filtering and trimming, MSAs of low quality are removed.

Individual gene trees were constructed for all filtered and trimmed MSAs using IQ-TREE (version 1.6.12) (Nguyen et al. 2015). Finally, all gene trees were concatenated into a single file, from which a species tree was generated using Astral III (version 5.7.3) (Zhang et al. 2018). The resulting species tree was manually rooting at the *Angophora/Corymbia* and *Eucalyptus* branch, using Figtree (version 1.4.4) (http://tree.bio.ed.ac.uk/software/figtree/).

## Data access

Sequencing data and reference genomes generated in this study have been submitted to the NCBI BioProject database (https://www.ncbi.nlm.nih.gov/bioproject/) under accession number PRJNA509734. Gene predictions, repeat annotations, and SyRI annotations generated in this study are available on FigShare (https://figshare.com/projects/Plant_genome_evolution_in_the_genus_Eucalyptus_driven_by_structural_rearrangements_that_promote_sequence_divergence/97010). All of the analysis scripts used in this study are available at GitHub (https://github.com/fergsc/33-Eucs) and as Supplemental Scripts.

## Supplementary Material

Supplement 1

Supplement 2

Supplement 3
