# Supplementary material for: Plant genome evolution in the genus Eucalyptus is driven by structural rearrangements that promote sequence divergence
Source: Genome Res. 2024 Apr;34(4):606–19. doi: 10.1101/gr.277999.123 (PMC11146599; doi:10.1101/gr.277999.123)
Supplement: Supplement 1 [file Supplementary_results.pdf]

Supplementary Results

Species Tree in Newick format

(E_shirleyi,((E_paniculata,(E_fibrosa,E_caleyi)0.93:0.03818940196774094)0.65:0.024618219050996856,((E_victrix,E_coolabah)1:0.13501517352840187,((E_leucophloia,(E_cladocalyx,(E_brandiana,((E_decipiens,E_virginea)1:0.17383604920002835,((E_pumila,(E_camaldulensis,(E_grandis,(E_globulus,E_viminalis)1:0.7374137843754482)1:0.13185695820026266)1:0.06546708650187055)1:1.1428633008653426,((E_microcorys,E_guilfoylei)1:0.06833655254303206,((E_erythrocorys,(C_maculata,(C_calophylla,A_floribunda)1:0.12607625812950585)1:4.876263306873159)0.36:0.0020176659618624977,((E_tenuipes,E_curtisii)1:0.0750975666719172,(E_cloeziana,(E_marginata,(E_ANBG9806169,(E_pauciflora,E_regnans)1:0.17462472627975154)1:1.249760502603425)0.43:0.09969136141117224)1:1.1168835379264237)1:0.3399612201488104)1:0.39999272937405755)1:0.5094427288635782)1:0.05598199781195624)1:0.44071244563817585)0.96:0.04579355413565084)0.83:0.055938305860067754)1:0.4977614606460969,((E_lansdowneana,(E_albens,(E_melliodora,(E_sideroxylon,E_melliodora_x_E_sideroxylon)1:0.3364035400804881)1:0.09678075007391215)0.94:0.04516806243295854)0.93:0.040626462682509606,(E_polyanthemos,E_dawsonii)1:0.5426175208391719)1:0.12001841168558196)0.57:0.03562032649218256)1:0.07349992296058556):0.0);

Guppy version, Canu version, NCBI ID and accession source

Species	Guppy version	Canu version	NCBI ID	Source	Accession
A. floribunda	3.3.0	1.9	GCA_014182895.1	ANU	
C. maculata	3.3.0	1.9	GCA_014182735.1	NAC	
E. brandiana	3.3.0	1.9	GCA_014182725.1	CCA	DN 5514
E. caleyi	3.3.0	1.9	GCA_014182885.1	CCA	DN 746
E. camaldulensis	3.3.0	1.9	GCA_014182705.1	ANBG	CBG 8401853
E. cladocalyx	4.0.11	2.0	GCA_017140615.1	CCA	DN 2569
E. cloeziana	3.3.0	1.9	GCA_014182715.1	ANBG	ANBG 68772.1
E. coolabah	3.3.0	1.9	GCA_014182585.1	ANBG	CBG 9404868.3
E. curtisii	4.0.11	2.0	GCA_017140595.1	ANBG	ANBG 68981
E. dawsonii	4.0.11	2.0	GCA_016097615.1	CCA	DN 743
E. decipiens	3.3.0	1.9	GCA_014182575.1	CCA	DN 3700
E. erythrocorys	3.3.0	1.9	GCA_014182555.1	ANBG	CBG 9806156
E. fibrosa	4.0.14	2.0	GCA_017140475.1	CCA	DN 666
E. globulus	3.3.0	1.9	GCA_014182545.1	UTAS	
E. grandis	3.3.0	1.9	GCA_016545825.1	ANBG	ANBG 69807
E. guilfoylei	4.0.14	2.0	GCA_016097605.1	CCA	DN 4632
E. lansdowneana	4.0.11	2.0	GCA_017140395.1	CCA	DN 5933
E. leucophloia	4.0.14	2.0	GCA_017140325.1	CCA	DN 2498
E. marginata	3.3.0	1.9	GCA_014182565.1	ANBG	CBG 9806545
E. melliodora x E. sideroxylon	3.3.0	2.0	GCA_016097485.1	CCA	1219
E. microcorys	3.3.0	1.9	GCA_014182515.1	ANBG	ANBG -224.1
E. ANBG9806169	3.3.0	1.9	GCA_014182395.1	ANBG	CBG 9806169
E. paniculata	4.0.11	2.0	GCA_017140255.1	CCA	639
E. pauciflora	4.0.15	2.0	#N/A	#N/A	#N/A
E. polyanthemos	4.0.11	2.0	GCA_017140185.1	CCA	742
E. pumila	4.0.11	2.0	GCA_016097595.1	CCA	636
E. regnans	3.3.0	1.9	GCA_014182855.1	TAS	Centurion
E. shirleyi	4.0.14	2.0	GCA_017140165.1	CCA	2508
E. tenuipes	3.3.0	1.9	GCA_014182365.1	ANBG	CBG 9807792.4
E. victrix	4.0.14	2.0	GCA_016097545.1	CCA	1178
E. viminalis	3.3.0	1.9	GCA_014182385.1	UTAS	
E. virginea	3.3.0	1.9	GCA_014182375.1	CCA	4635

ANBG: Australian National Botanic Gardens, Canberra, Australian Capital Territory (ACT), Australia. Indigenous Ngunnawal country.
CCA: Currency Creek Arboretum, Currency Creek, South Australia, Australia.

ANU: Australian National University, Acton, ACT, Australia.
NAC: the National Arboretum Canberra, Molonglo Valley, ACT, Australia.
UTAS: the University of Tasmania, Sandy Bay, Tasmania, Australia.
TAS: Tasmania, Eucalyptus woodlands of southern Tasmania.

Assessment of Scaffolding

Scaffolding of all genomes within this study used homology to *E. grandis* (Myburg *et al.*, 2014), the only available scaffolding information. Using this external and non-species specific data source for scaffolding can potentially introduce false positive and false negative rearrangements, altering synteny. To assess potential impacts the scaffolding method had on our results we scaffolded *E. melliodora* using individual-specific Hi-C and reanalysed our genome alignments and annotations.

Hi-C sequencing of *E. melliodora* generated 45.48 Gbp in 151,590,503 paired reads, giving an estimated genome coverage of 71.14x. After aligning Hi-C reads to *E. melliodora* contigs and identifying PCR duplicates 18,507,548 (12.21%) of read pairs were found to contain linkage information. Further examination showed that 9,612,532 (6.34%) read pairs spanned contigs, and 8,895,016 (5.87%) of read pairs were contained within a single contig. Non-informative reads were either chimeric, unmapped, PCR duplicates, or had low mapping quality (MAPQ < 30, mostly due to multi-mapping). Using 3D-DNA, the *E. melliodora* contigs were scaffolded, placing 97.60% of its genome within 11 scaffolds, Figure 1.

The vast majority of contigs between the two differentially scaffolded *E. melliodora* genomes were found to be identically grouped, ordered, and orientated within the 11 scaffolds, Figure 2. However, a small number of contigs, while correctly grouped and ordered, are incorrectly orientated. Similarly, a small number of contigs, while correctly grouped and orientated, are incorrectly ordered within chromosomes.

To examine the potential impact the incorrectly orientated and ordered contigs had upon our results we repeated our comparative phylogenetic analysis. All other Eucalyptus genomes were aligned to Hi-C *E. melliodora* and both genomes annotated for synteny, rearrangements, and unaligned regions giving 64 annotated genomes, as per our main methods. The proportion of synteny, unaligned, inverted, translocated, and duplicated was plotted against the genomes' phylogenetic distance and regressions calculated. These results were compared to those obtained homology scaffolded *E. melliodora*, Figure 3 and Table 1. Comparing the syntenic trendlines, homology scaffolding was found to inflate the proportion of syntenic content, however the rate of synteny loss as phylogenetic distance increases was nearly identical. The unaligned genome proportion and rate of accumulation was inflated using homology scaffolding, however unaligned still significantly rose as phylogenetic distance increased. Hi-C left more contigs unplaced than did homology scaffolding, reducing unaligned within the Hi-C genome. Inversions were more frequent within the Hi-C genome, however they still occupied a minority of the genome, and neither increased or decreased as phylogenetic distance increased. Translocations occupied a similar proportion of genomes, however the rate of loss was not found to be significant when Hi-C scaffolded. Finally, duplications gave a very similar result and were significantly lost using both scaffolding methods.

In summary, homology scaffolding against *E. grandis* retained the majority of genome architecture. Syntenic, rearranged, and unaligned regressions were raised and lowered, but only translocations saw a loss of significance. Translocations may not be contributing to genome divergence as significantly as homology scaffolding predicted. However, in this comparison the phylogenetic distance between E. grandis and E. melliodora is among our

largest. Performing this analysis using E. melliodora is almost our worst-case scenario, 24 of our genomes will be less diverged, and only 8 more diverged.

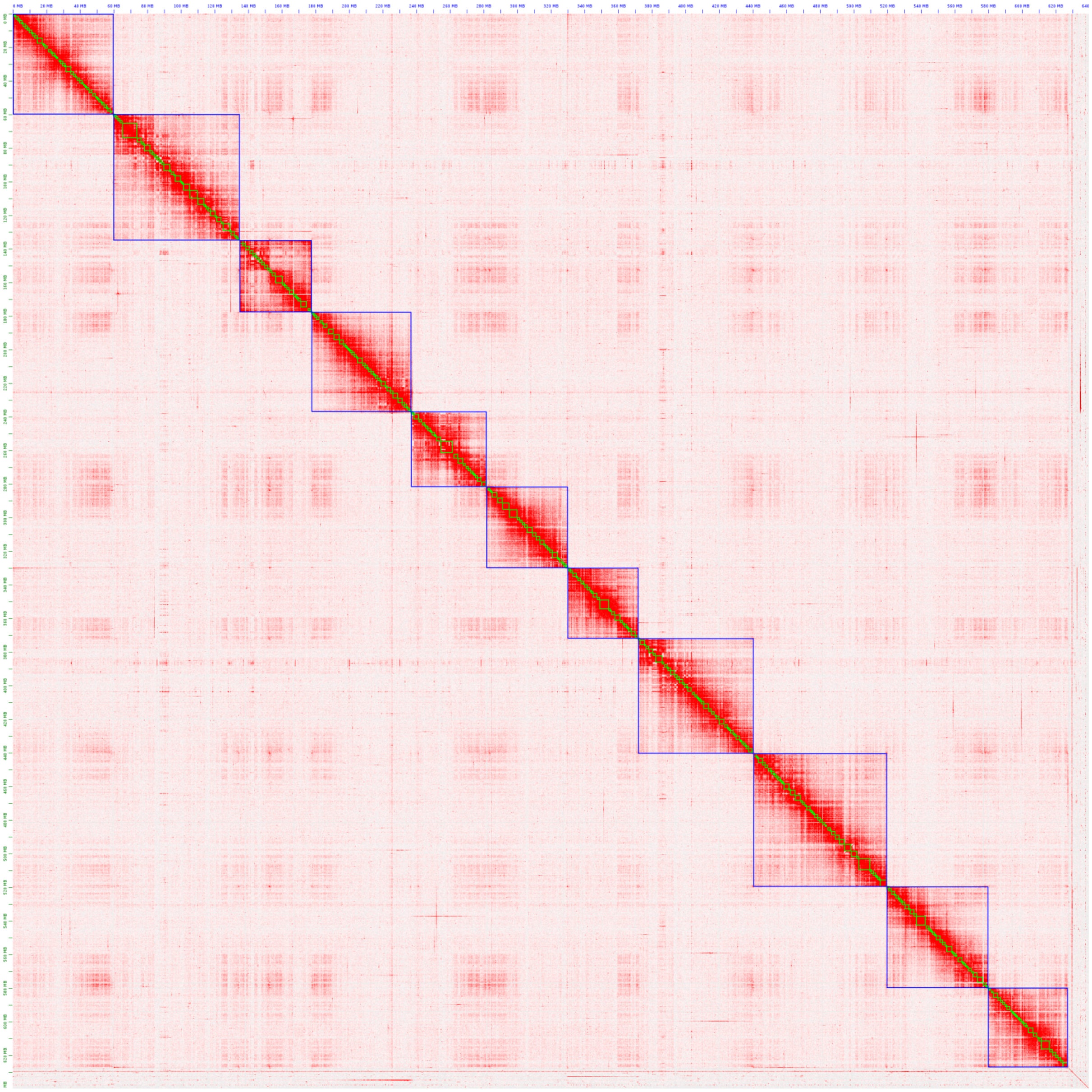

Figure 1. Manually curated, final, Hi-C contact map of E. melliodora's scaffolded contigs. Contigs were scaffolded using 3D DNA (parameter: "--editor-repeat-coverage 5, -i 1000) (Dudchenko *et al.*, 2017) and visualised with Juicebox (Durand *et al.*, 2016).

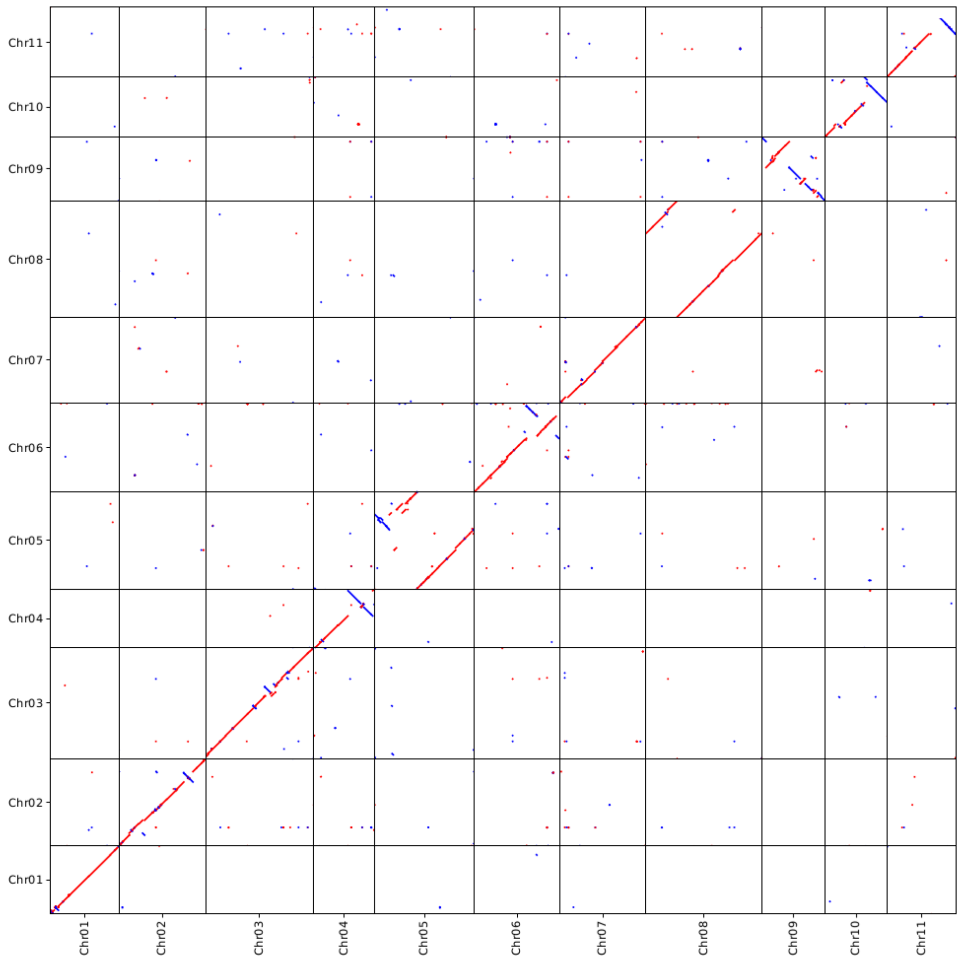

Figure 2. Dot plot showing sequence homology between *E. melliodora* scaffolded using Hi-C and scaffolded using homology to *E. grandis*. The majority of contigs are identically grouped, oriented, and ordered within scaffolds. However, several inversions can be observed along with a smaller number of translocations.

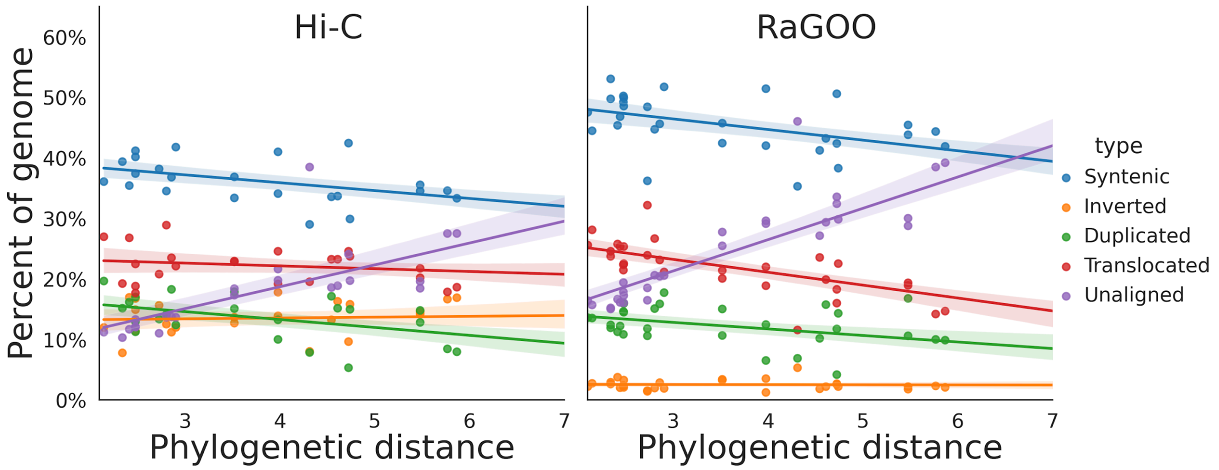

Figure 3. Pairwise genome conservation and loss, as phylogenetic distance increases. The proportion of both Eucalyptus genomes within an alignment pair that was identified as syntenic, unaligned, inverted, translocated, or duplicated plotted against the phylogenetic distance of the two genomes. The unaligned proportion is the species-specific fraction of the genome between genome pairs, resulting from either an insertion, deletion,

differential inheritance, or sequence divergence. When combined, the proportion of sequence that is syntenic, unaligned, and rearranged equals 100% for each genome within an alignment pair. The rearranged fraction is further broken down into inverted, translocated, and duplicated regions. Phylogenetic distance was calculated as the sum of branch lengths between each genome pair within phylogeny. P-value tests if the slope of the regression line is nonzero.

	**Hi-C**	**RaGOO**
**Syntenic**	*0.320	*0.340
**Unaligned**	*0.645	*0.802
**Inverted**	0.006	0.002
**Duplicated**	*0.327	*0.263
**Translocated**	0.064	*0.564

Table 1. R2 values for Figure 3, comparing the rate of loss or gain of the proportion of the genome syntenic, unaligned, inverted, translocated, and duplicated. * indicates a significant result (p>= 0.05).

References

Dudchenko, O. *et al.* (2017) ‘De novo assembly of the Aedes aegypti genome using Hi-C yields chromosome-length scaffolds’, *Science*, 356(6333), pp. 92–95. Available at: https://doi.org/10.1126/science.aal3327.

Durand, N.C. *et al.* (2016) ‘Juicebox Provides a Visualization System for Hi-C Contact Maps with Unlimited Zoom’, *Cell Systems*, 3(1), pp. 99–101. Available at: https://doi.org/10.1016/j.cels.2015.07.012.

Myburg, A.A. *et al.* (2014) ‘The genome of Eucalyptus grandis’, *Nature*, 510(7505), pp. 356–362. Available at: https://doi.org/10.1038/nature13308.
