# Supplementary material for: Plant genome evolution in the genus Eucalyptus is driven by structural rearrangements that promote sequence divergence
Source: Genome Res. 2024 Apr;34(4):606–19. doi: 10.1101/gr.277999.123 (PMC11146599; doi:10.1101/gr.277999.123)
Supplement: Supplement 2 [file Supplementary_tables-figures.pdf]

Supplementary Figures and Tables

Species	DNA extraction method	Size selection (kbp)	Actual Coverage	N50 (kbp)	Number of reads	Total size (Mbp)	Longest read (kbp)
A. floribunda	Nuclei	20	68.5	30,913	1,165,378	26,218.73	234.42
C. maculata	Nuclei	20	62.0	32,785	1,068,357	24,727.52	186.19
E. brandiana	Nuclei	20	59.0	25,064	1,572,473	29,661.57	185.11
E. caleyi	Sorbitol	20	44.2	38,986	981,871	25,568.27	245.74
E. camaldulensis	Nuclei	20	43.1	26,925	1,246,437	23,709.64	236.32
E. cladocalyx	Nuclei	20	44.3	22,608	1,603,737	23,813.99	871.14
E. cloeziana	Nuclei	20	35.8	23,441	1,106,171	16,977.71	202.46
E. coolabah	Nuclei	40	33.5	38,840	843,066	19,942.69	258.17
E. curtisii	Nuclei	20	61.2	26,200	1,482,403	26,364.96	184.51
E. dawsonii	Sorbitol	20	31.4	39,210	833,846	21,893.54	340.53
E. decipiens	Nuclei	20	36.9	29,788	937,250	21,488.84	241.66
E. erythrocorys	Nuclei	20	43.4	30,515	994,422	23,167.70	201.42
E. fibrosa	Sorbitol	20	39.1	40,269	808,634	22,664.55	239.76
E. globulus	Nuclei	20	27.5	26,580	789,684	14,775.47	196.77
E. grandis	Nuclei	40	26.6	40,622	566,332	16,120.84	223.69
E. guilfoylei	Sorbitol	20	44.9	37,750	748,484	21,030.20	238.04
E. lansdowneana	Sorbitol	20	33.5	40,454	766,963	20,885.35	264.89
E. leucophloia	Nuclei	20	42.3	31,376	1,112,561	23,719.81	220.51
E. marginata	Nuclei	20	37.6	19,330	2,030,612	18,996.49	403.50
E. melliodora x E. sideroxylon	Sorbitol	20	63.5	28,218	2,096,002	38,010.87	493.59
E. microcorys	Nuclei	20	83.8	26,233	1,891,415	36,501.91	259.59
E. ANBG9806169	Nuclei	20	45.0	23,768	1,361,586	22,622.88	125.92
E. paniculata	Sorbitol	20	37.5	36,669	878,971	21,750.73	223.73
E. pauciflora	NA	NA	72.3	26.93	2,583,680	35,691.93	537.11
E. polyanthemos	Sorbitol	20	43.8	30,700	1,317,377	26,018.59	220.24
E. pumila	Nuclei	20	40.7	25,051	1,632,628	21,358.64	165.40
E. regnans	Sorbitol	40	47.9	46,297	718,316	23,254.44	253.70
E. shirleyi	Sorbitol	20	44.3	44,993	862,028	26,095.39	273.96
E. tenuipes	Nuclei	40	48.7	41,950	663,327	19,070.76	296.92
E. victrix	Sorbitol	20	50.6	41,561	1,149,096	27,764.76	270.78
E. viminalis	Nuclei	20	32.0	22,308	1,535,956	17,640.60	163.61
E. virginea	Nuclei	20	47.7	25,319	1,406,739	25,089.92	164.74

Supplementary Table S1. Raw read library statistics. Actual coverage is calculated after assembly from the final genome size.

Species	Coverage	Coverage lost	N50 (kbp)	Number of reads	Total size (Mbp)	Longest read (kbp)
A. floribunda	62.9	5.6	30,973	961,453	24,080.30	234.02
C. maculata	58.0	4	32,784	902,490	23,154.27	185.79
E. brandiana	54.7	4.3	24,964	1,318,778	27,465.22	184.71
E. caleyi	41.1	3.1	39,257	797,371	23,780.80	245.34
E. camaldulensis	39.8	3.3	26,890	1,036,444	21,912.23	235.92
E. cladocalyx	40.3	4	22,553	1,357,574	21,658.32	167.28
E. cloeziana	32.4	3.4	23,421	891,520	15,359.12	150.84
E. coolabah	31.1	2.4	39,047	696,864	18,498.71	257.77
E. curtisii	56.8	4.4	26,212	1,260,701	24,482.11	184.11
E. dawsonii	29.1	2.3	39,530	705,446	20,309.35	340.13
E. decipiens	34.8	2.1	29,702	818,918	20,220.97	232.70
E. erythrocorys	40.1	3.3	30,501	854,347	21,394.22	201.02
E. fibrosa	36.7	2.4	40,481	710,154	21,285.37	239.36
E. globulus	24.7	2.8	26,783	625,059	13,252.63	196.37
E. grandis	24.7	1.9	40,618	478,371	14,946.13	223.29
E. guilfoylei	41.8	3.1	37,759	663,545	19,576.72	237.64
E. lansdowneana	31.1	2.4	40,677	662,726	19,393.64	264.49
E. leucophloia	39.4	2.9	31,506	966,679	22,088.48	220.11
E. marginata	32.6	5	19,997	1,441,634	16,472.64	266.42
E. melliodora x E. sideroxylon	58.3	5.2	28,356	1,760,405	34,919.15	203.52
E. microcorys	78.0	5.8	26,255	1,628,396	33,963.49	259.19
E. ANBG9806169	41.3	3.7	23,609	1,134,657	20,769.73	125.52
E. paniculata	35.3	2.2	36,846	769,367	20,476.85	223.33
E. pauciflora	73.3	0	26,926	2,583,680	35,691.93	537.11
E. polyanthemos	40.4	3.4	30,888	1,121,971	24,048.44	219.84
E. pumila	36.8	3.9	25,254	1,217,779	19,269.00	165.00
E. regnans	45.6	2.3	46,254	611,946	22,142.04	212.45
E. shirleyi	41.7	2.6	45,127	740,464	24,593.44	273.56
E. tenuipes	46.0	2.7	41,997	574,526	18,007.59	296.52
E. victrix	46.9	3.7	42,005	923,073	25,731.32	266.30
E. viminalis	28.8	3.2	22,386	955,619	15,865.49	163.21
E. virginea	44.4	3.3	25,299	1,196,234	23,361.01	164.34

Supplementary Table S2. Filtered read library statistics. Statistics describing read libraries after trimming, length filtering, and quality filtering. Note: *E. pauciflora* reads were filtered and trimmed before being randomly down sampled, hence no loss of sequence.

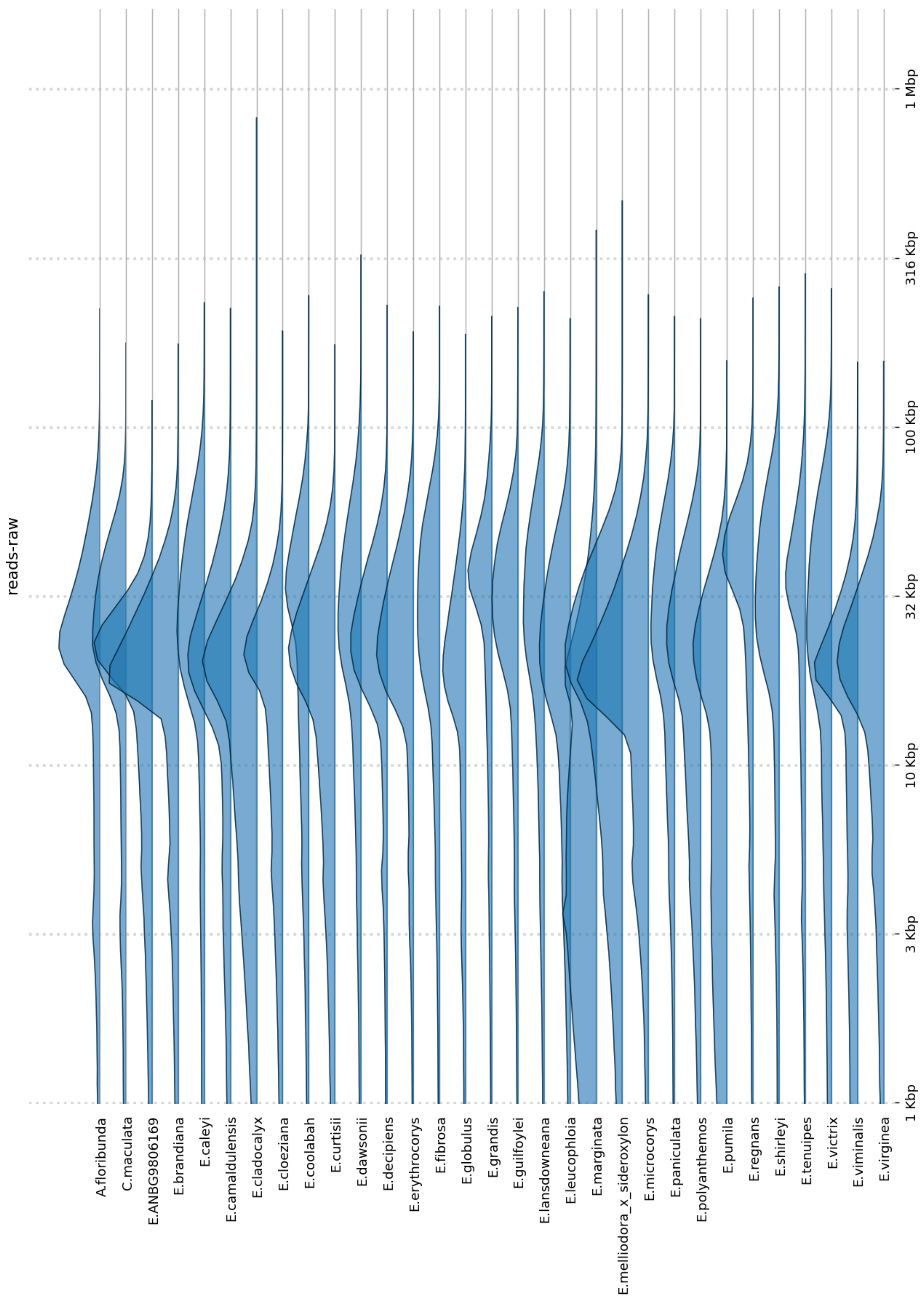

Supplementary Figures S1. Distribution of read lengths of raw read libraries

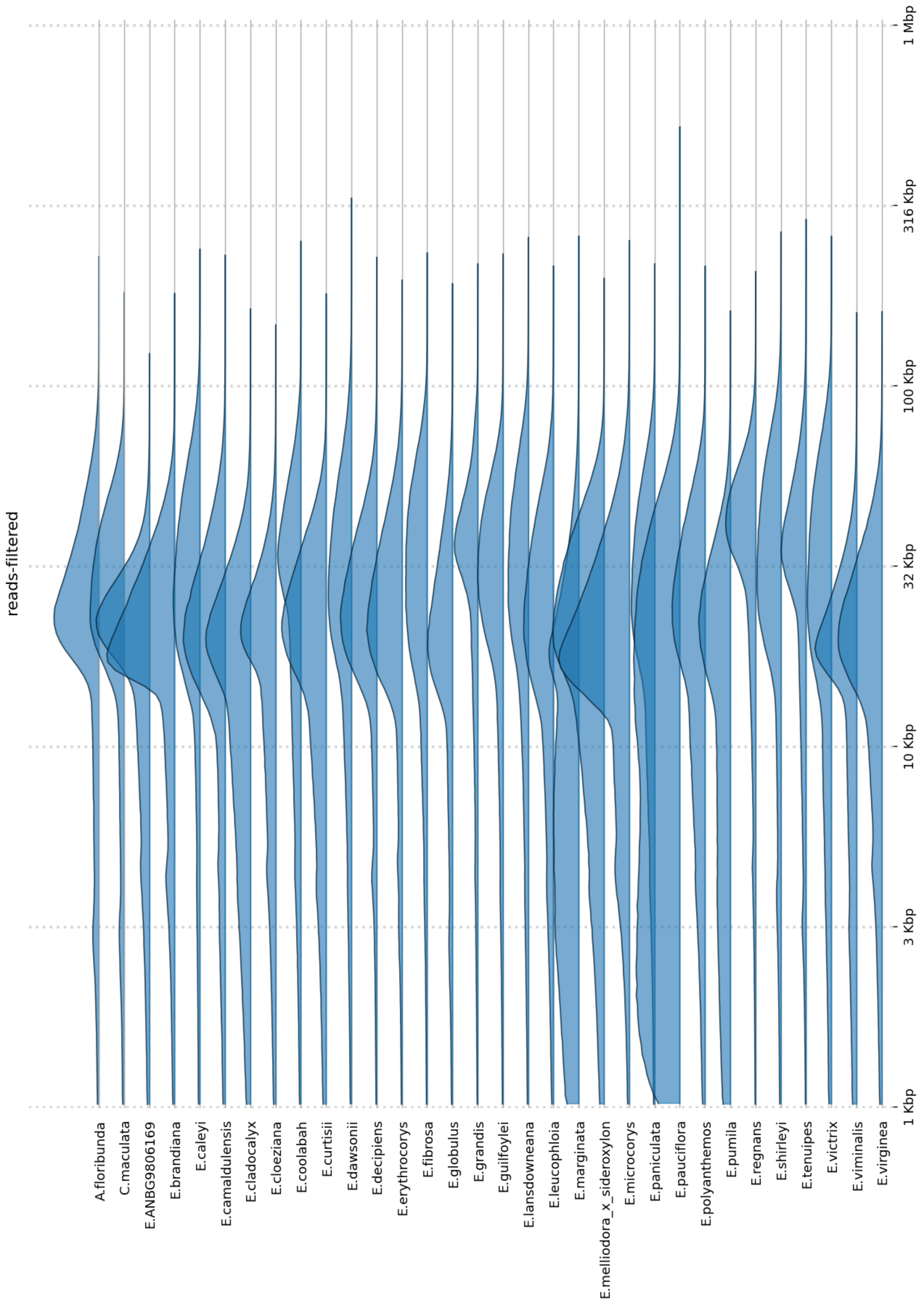

Supplementary Figures S2. Distribution of read lengths of read libraries aftering trimming, length filtering, and quality filtering.

	Contamination	Haplotigs		Assembly artifacts	
**Species**	**Total Size (kbp)**	**Total Size (Mbp)**	**% of Genome**	**Total Size (Mbp)**	**% of Genome**
A. floribunda	None	253.37	36.74%	53.73	7.79%
C. maculata	None	265.09	36.74%	57.48	7.97%
E. brandiana	161.18	38.62	7.01%	10.05	1.82%
E. caleyi	3,448.47	458.40	42.15%	46.71	4.29%
E. camaldulensis	None	394.82	39.70%	49.34	4.96%
E. cladocalyx	33.10	383.48	41.54%	1.86	0.20%
E. cloeziana	None	188.67	27.07%	33.94	4.87%
E. coolabah	None	381.48	38.25%	21.07	2.11%
E. curtisii	None	234.46	35.06%	3.51	0.53%
E. dawsonii	18,227.31	426.59	37.01%	9.88	0.86%
E. decipiens	40.93	384.89	37.82%	51.03	5.02%
E. erythrocorys	None	115.73	16.78%	40.38	5.86%
E. fibrosa	1.38	553.42	48.66%	3.49	0.31%
E. globulus	None	228.73	29.17%	18.52	2.36%
E. grandis	None	280.46	30.84%	23.47	2.58%
E. guilfoylei	None	93.14	16.44%	4.66	0.82%
E. lansdowneana	7.00	463.32	42.41%	5.02	0.46%
E. leucophloia	241.53	446.00	44.08%	4.61	0.46%
E. marginata	34.66	290.90	35.09%	32.13	3.88%
E. melliodora x E. sideroxylon	133.04	507.89	45.74%	3.94	0.36%
E. microcorys	133.10	228.43	30.66%	81.15	10.89%
E. ANBG9806169	None	231.78	30.09%	36.05	4.68%
E. paniculata	249.95	501.04	46.14%	4.47	0.41%
E. pauciflora	107.77	441.32	47.36%	3.62	0.39%
E. polyanthemos	None	552.62	48.03%	3.35	0.29%
E. pumila	42.45	272.67	34.07%	3.18	0.40%
E. regnans	267.24	370.84	40.60%	56.32	6.17%
E. shirleyi	None	442.43	42.74%	3.67	0.35%
E. tenuipes	None	204.32	31.75%	47.28	7.35%
E. victrix	None	482.44	46.63%	3.68	0.36%
E. viminalis	43.43	296.85	34.18%	20.42	2.35%
E. virginea	None	281.01	32.69%	52.26	6.08%
**Average**	724.14	334.23	36.15%	24.70	3.04%

Supplementary Table S3. Amount and proportion of sequence removed from genomes during contamination filtering, haplotig purging, and artifact filtering.

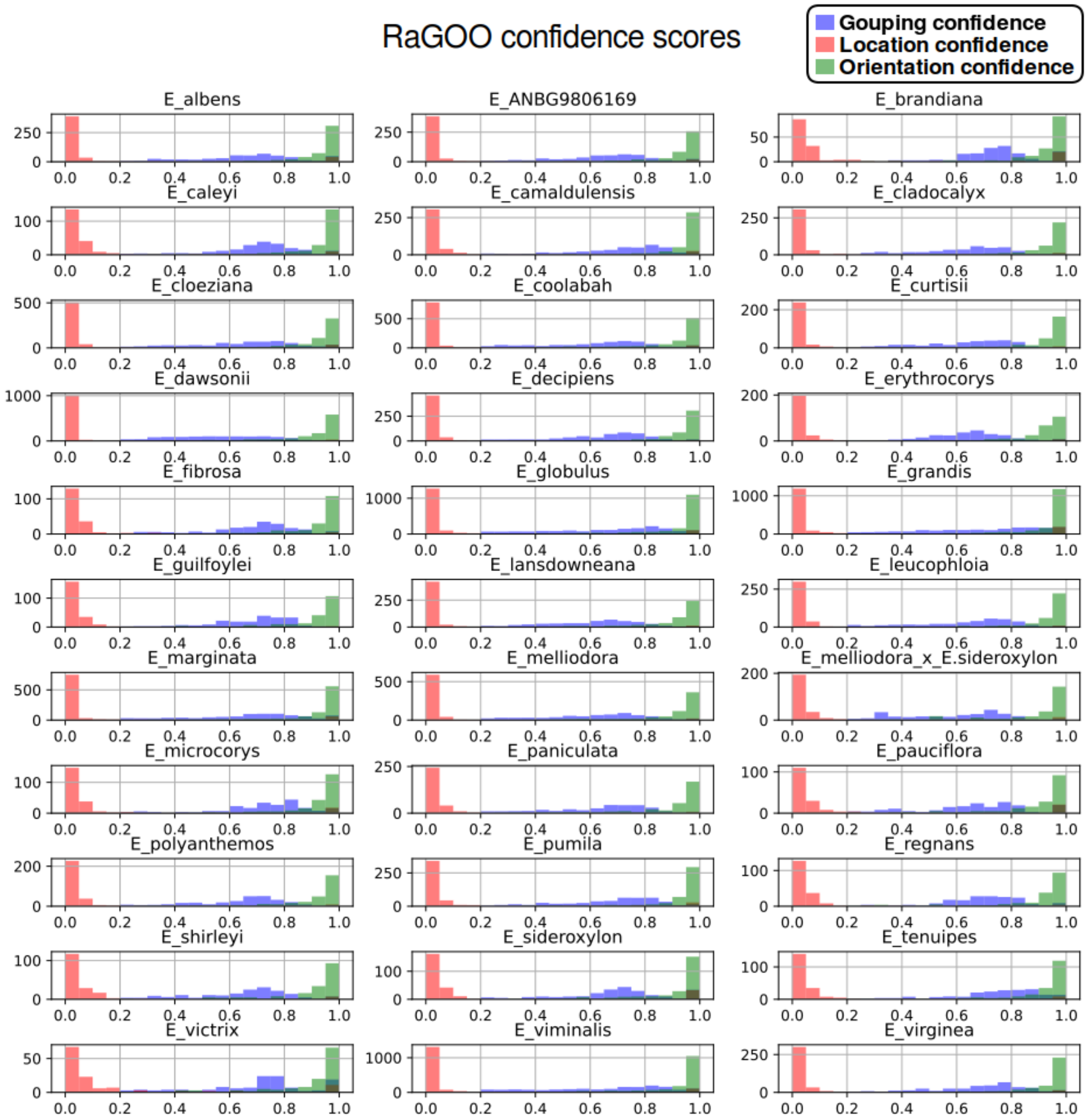

Supplementary Figure S3. Confidence scores reported by RaGOO during scaffolding of genomes. Grouping confidence = assigning contigs to a chromosome; location confidence = ordering contigs relative to each other along the scaffold; orientation confidence = orienting contigs within scaffolds.

species	Complete (single-copy + duplicated)	single-copy	duplicated	fragmented	missing	LAI
A. floribunda	96.82%	92.78%	4.04%	1.33%	1.85%	14.5
C. maculata	97.25%	94.93%	2.32%	1.12%	1.63%	15.92
E. brandiana	98.11%	95.06%	3.05%	1.03%	0.86%	23.85
E. caleyi	96.47%	90.80%	5.67%	1.59%	1.93%	18.24
E. camaldulensis	96.73%	92.09%	4.64%	1.33%	1.93%	16.99
E. cladocalyx	97.59%	93.29%	4.30%	1.12%	1.29%	18.53
E. cloeziana	97.12%	93.55%	3.57%	1.59%	1.29%	19.06
E. coolabah	95.44%	88.52%	6.92%	2.15%	2.41%	15.9
E. curtisii	97.29%	93.21%	4.08%	1.42%	1.29%	18.34
E. dawsonii	97.51%	83.10%	14.40%	0.95%	1.55%	17.01
E. decipiens	96.99%	91.49%	5.50%	1.33%	1.68%	18.87
E. erythrocorys	97.55%	94.41%	3.14%	0.95%	1.50%	20.18
E. fibrosa	96.73%	87.96%	8.77%	1.63%	1.63%	17.49
E. globulus	96.69%	91.87%	4.82%	1.25%	2.06%	17.46
E. grandis	96.09%	90.11%	5.98%	1.59%	2.32%	17.11
E. guilfoylei	98.02%	94.84%	3.18%	0.90%	1.07%	16.39
E. lansdowneana	97.12%	86.76%	10.36%	1.12%	1.76%	19.46
E. leucophloia	96.99%	90.58%	6.41%	1.29%	1.72%	17.91
E. marginata	96.17%	90.93%	5.25%	1.72%	2.11%	19.58
E. melliodora x E. sideroxylon	97.72%	91.70%	6.02%	1.03%	1.25%	17.96
E. microcorys	97.21%	93.90%	3.31%	1.25%	1.55%	16.2
E. ANBG9806169	96.86%	93.29%	3.57%	1.29%	1.85%	22.16
E. paniculata	97.12%	90.58%	6.53%	1.20%	1.68%	18.58
E. pauciflora	97.25%	93.29%	3.96%	1.25%	1.50%	20.29
E. polyanthemos	96.82%	89.85%	6.96%	1.55%	1.63%	17.52
E. pumila	97.38%	93.29%	4.08%	1.42%	1.20%	17.74
E. regnans	97.25%	93.47%	3.78%	1.25%	1.50%	20.18
E. shirleyi	97.29%	88.99%	8.30%	1.16%	1.55%	19.89
E. tenuipes	96.39%	92.56%	3.83%	1.81%	1.81%	15.07
E. victrix	96.65%	91.32%	5.33%	1.46%	1.89%	18.71
E. viminalis	96.47%	91.83%	4.64%	1.29%	2.24%	16.5
E. virginea	97.08%	92.30%	4.77%	1.16%	1.76%	17.69

Supplementary Table S4. BUSCO and LAI scores for all genomes.

	N50 (kbp)	Number of contigs	Size (Mbp)	Longest contig (Mbp)
A. floribunda	1,139.19	2,857	689.71	10.21
C. maculata	1,540.02	3,174	721.55	12.94
E. brandiana	6,578.56	802	551.18	24.53
E. caleyi	1,805.12	2,810	1,087.60	25.88
E. camaldulensis	939.69	4,219	994.59	8.75
E. cladocalyx	953.07	4,591	923.15	16.82
E. cloeziana	855.51	4,172	697.06	7.22
E. coolabah	552.28	4,066	997.21	5.38
E. curtisii	1,332.99	2,628	668.67	16.95
E. dawsonii	474.20	4,951	1,152.64	8.02
E. decipiens	747.03	4,718	1,017.59	6.82
E. erythrocorys	2,931.08	2,208	689.47	18.40
E. fibrosa	1,730.72	1,790	1,137.28	24.72
E. globulus	317.08	5,378	784.04	3.78
E. grandis	321.42	5,311	909.45	4.43
E. guilfoylei	3,303.65	1,041	566.64	21.44
E. lansdowneana	1,031.71	2,572	1,092.36	13.56
E. leucophloia	1,038.45	3,108	1,011.89	12.88
E. marginata	458.19	5,121	828.93	4.33
E. melliodora x E. sideroxylon	1,719.77	2,555	1,110.47	21.42
E. microcorys	1,549.62	3,462	745.17	13.19
E. ANBG9806169	1,006.91	4,115	770.36	14.88
E. paniculata	1,259.10	2,337	1,085.95	17.29
E. pauciflora	1,379.37	2,654	931.94	23.39
E. polyanthemos	1,287.56	2,369	1,150.67	22.17
E. pumila	1,064.83	3,313	800.22	15.45
E. regnans	1,571.93	2,834	913.33	22.03
E. shirleyi	2,635.00	1,462	1,035.28	25.64
E. tenuipes	1,548.79	2,406	643.48	16.67
E. victrix	3,210.46	1,298	1,034.58	36.56
E. viminalis	289.36	7,315	868.55	4.50
E. virginea	1,222.18	3,963	859.54	11.49

Supplementary Table S5. Raw genome statistics. Genomes have not been curated in any way.

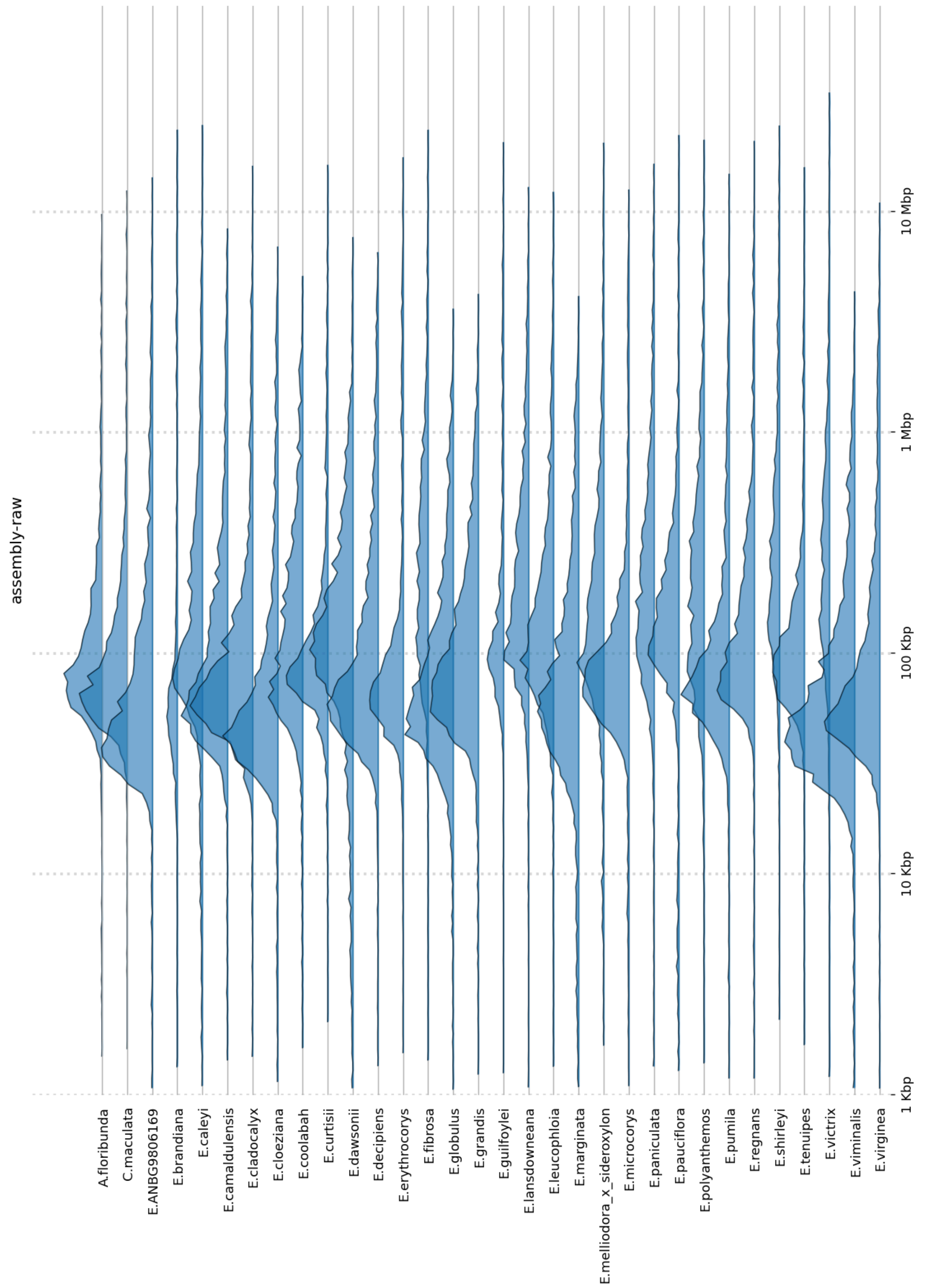

Supplementary Figure S4. Raw genome contig distributions. Genomes have not been curated in any way.

	N50 (kbp)	Number of contigs	Size (Mbp)	Longest contig (Mbp)
A. floribunda	4,011.83	225	388.67	10.21
C. maculata	4,690.14	176	404.38	12.94
E. brandiana	7,278.40	170	507.20	24.53
E. caleyi	4,769.92	276	589.30	25.88
E. camaldulensis	2,481.60	420	558.53	8.75
E. cladocalyx	2,796.55	390	544.04	16.82
E. cloeziana	1,739.94	626	480.25	7.22
E. coolabah	1,289.21	937	606.36	5.38
E. curtisii	2,961.00	288	435.23	16.95
E. dawsonii	985.62	1,343	706.79	8.02
E. decipiens	1,985.74	553	590.96	6.82
E. erythrocorys	4,024.36	253	539.36	18.40
E. fibrosa	6,447.41	192	589.89	24.72
E. globulus	636.15	1,750	545.00	3.78
E. grandis	613.64	1,749	616.23	4.43
E. guilfoylei	4,251.44	209	472.34	21.44
E. lansdowneana	2,352.90	489	633.47	13.56
E. leucophloia	2,659.36	382	568.44	12.88
E. marginata	1,011.74	991	513.33	4.33
E. melliodora x E. sideroxylon	6,217.05	282	603.80	21.42
E. microcorys	4,004.79	234	440.96	13.19
E. ANBG9806169	2,396.19	477	507.94	14.88
E. paniculata	3,699.06	330	588.82	17.29
E. pauciflora	6,583.95	216	494.01	23.39
E. polyanthemos	4,657.75	300	603.25	22.17
E. pumila	2,492.89	473	529.71	15.45
E. regnans	5,259.26	206	495.05	22.03
E. shirleyi	6,912.49	181	597.16	25.64
E. tenuipes	3,433.87	211	398.08	16.67
E. victrix	11,095.62	120	557.15	36.56
E. viminalis	652.95	1,759	558.84	4.50
E. virginea	2,391.64	379	532.97	11.49

Supplementary Table S6. Final genome statistics. Genomes have been fully curated, i.e. all contaminate, haplotig, plastid, and artifact contigs have been removed. Genomes have also been polished.

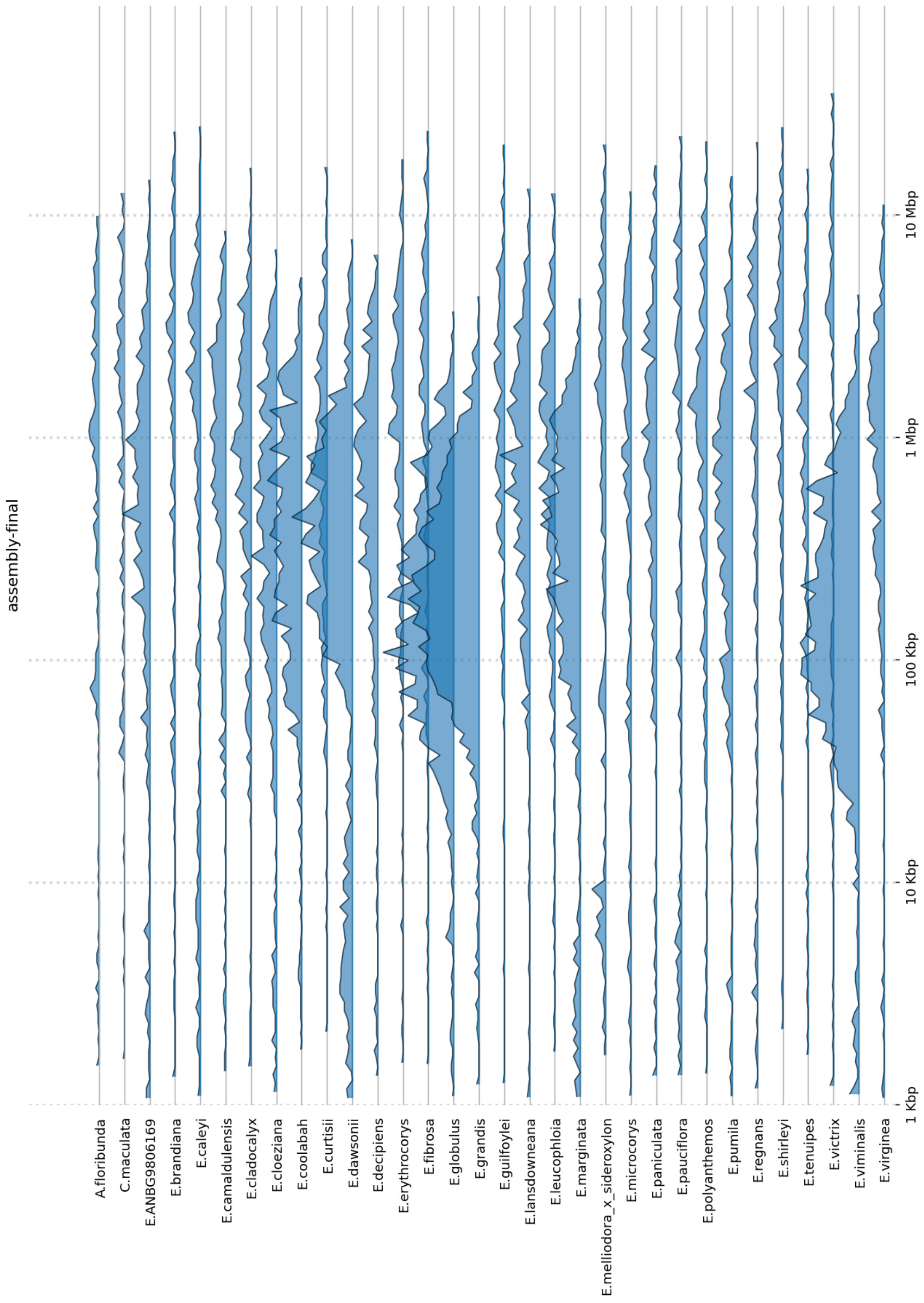

Supplementary Figure S5. Final genome contig distributions. Genomes have been fully curated, i.e. all contaminate, haplotig, plastid, and artifact contigs have been removed. Genomes have also been polished.

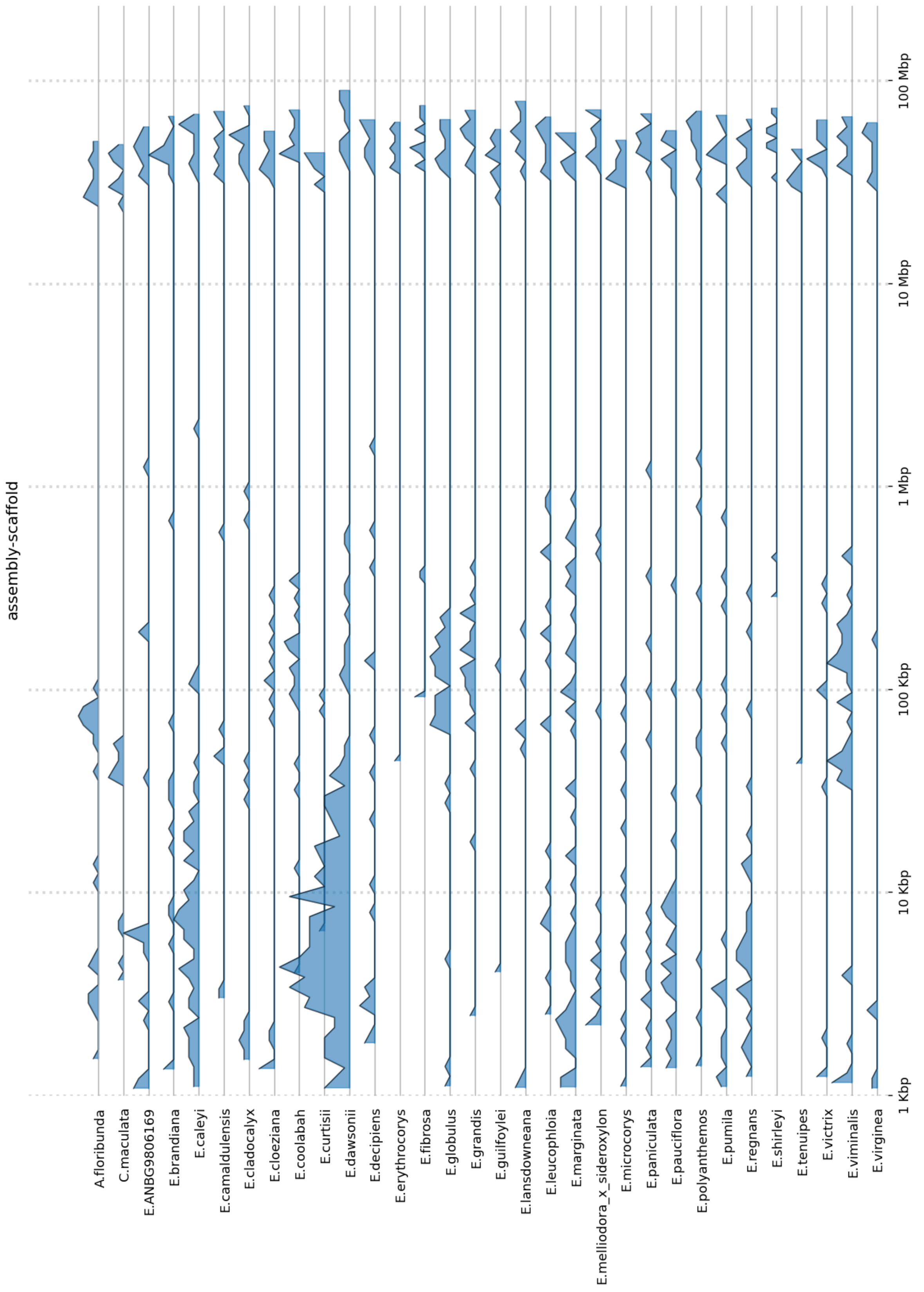

Supplementary Figure S6. Scaffolded genome contig distributions.

	Proportion of TE	Proportion of simple repeats	Total repeat proportion
A. floribunda	34.55%	1.26%	35.81%
C. calophylla	34.79%	1.14%	35.93%
E. brandiana	44.21%	1.24%	45.45%
E. caleyi	46.00%	1.19%	47.19%
E. camaldulensis	45.31%	1.25%	46.56%
E. cladocalyx	45.85%	1.28%	47.13%
E. cloeziana	42.57%	1.25%	43.82%
E. coolabah	45.89%	1.27%	47.16%
E. curtisii	41.66%	1.31%	42.97%
E. dawsonii	45.88%	1.17%	47.05%
E. decipiens	46.95%	1.19%	48.14%
E. erythrocorys	47.07%	1.30%	48.37%
E. fibrosa	45.10%	1.22%	46.32%
E. globulus	44.29%	1.23%	45.52%
E. grandis	46.53%	1.17%	47.70%
E. guilfoylei	41.22%	1.20%	42.42%
E. lansdowneana	46.10%	1.27%	47.37%
E. leucophloia	44.37%	1.28%	45.65%
E. marginata	43.43%	1.21%	44.64%
E. melliodora x E. sideroxylon	46.71%	1.19%	47.90%
E. microcorys	41.39%	1.31%	42.70%
E. ANBG9806169	44.00%	1.21%	45.21%
E. paniculata	44.92%	1.32%	46.24%
E. pauciflora	43.10%	1.23%	44.33%
E. polyanthemos	45.55%	1.23%	46.78%
E. pumila	44.17%	1.28%	45.45%
E. regnans	43.06%	1.20%	44.26%
E. shirleyi	45.85%	1.25%	47.10%
E. tenuipes	37.82%	1.34%	39.16%
E. victrix	44.34%	1.39%	45.73%
E. viminalis	44.57%	1.19%	45.76%
E. virginea	43.78%	1.29%	45.07%

Supplementary Table S7. Repeat content of all genomes.

Species	Number of genes	Genes in orthogroup	Unassigned genes	Paralogue groups	Genes in paralogues
E. albens	65,121	63,903 (98.13%)	1,218 (1.87%)	43	94 (0.14%)
E. brandiana	46,176	45,632 (98.82%)	544 (1.18%)	20	44 (0.1%)
E. caleyi	62,000	60,606 (97.75%)	1,394 (2.25%)	59	143 (0.23%)
E. camaldulensis	51,855	50,949 (98.25%)	906 (1.75%)	33	80 (0.15%)
E. cladocalyx	48,487	47,797 (98.58%)	690 (1.42%)	30	68 (0.14%)
E. cloeziana	43,093	42,349 (98.27%)	744 (1.73%)	26	61 (0.14%)
E. coolabah	51,879	50,855 (98.03%)	1,024 (1.97%)	29	62 (0.12%)
E. curtisii	43,242	42,182 (97.55%)	1,060 (2.45%)	79	194 (0.45%)
E. dawsonii	77,763	73,579 (94.62%)	4,184 (5.38%)	207	481 (0.62%)
E. decipiens	49,909	49,012 (98.2%)	897 (1.8%)	43	101 (0.2%)
E. erythrocorys	47,392	45,659 (96.34%)	1,733 (3.66%)	299	966 (2.04%)
E. fibrosa	53,954	53,276 (98.74%)	678 (1.26%)	18	40 (0.07%)
E. globulus	51,735	50,781 (98.16%)	954 (1.84%)	23	47 (0.09%)
E. grandis	55,224	53,939 (97.67%)	1,285 (2.33%)	50	114 (0.21%)
E. guilfoylei	54,024	52,853 (97.83%)	1,171 (2.17%)	95	236 (0.44%)
E. lansdowneana	59,574	58,536 (98.26%)	1,038 (1.74%)	42	93 (0.16%)
E. leucophloia	53,950	52,869 (98%)	1,081 (2%)	43	101 (0.19%)
E. marginata	48,255	47,059 (97.52%)	1,196 (2.48%)	61	151 (0.31%)
E. melliodora	63,907	62,839 (98.33%)	1,068 (1.67%)	88	224 (0.35%)
E. melliodora x E. sideroxylon	64,299	63,447 (98.67%)	852 (1.33%)	35	73 (0.11%)
E. microcorys	44,367	43,578 (98.22%)	789 (1.78%)	34	88 (0.2%)
E. ANBG9806169	48,845	47,891 (98.05%)	954 (1.95%)	55	134 (0.27%)
E. paniculata	55,821	55,011 (98.55%)	810 (1.45%)	25	57 (0.1%)
E. pauciflora	47,634	46,796 (98.24%)	838 (1.76%)	59	156 (0.33%)
E. polyanthemos	56,111	55,253 (98.47%)	858 (1.53%)	21	44 (0.08%)
E. pumila	59,604	58,260 (97.75%)	1,344 (2.25%)	63	142 (0.24%)

E. regnans	45,608	44,753 (98.13%)	855 (1.87%)	49	120 (0.26%)
E. shirleyi	55,816	55,022 (98.58%)	794 (1.42%)	34	81 (0.15%)
E. sideroxylon	60,360	59,278 (98.21%)	1,082 (1.79%)	27	59 (0.1%)
E. tenuipes	41,622	40,587 (97.51%)	1,035 (2.49%)	50	123 (0.3%)
E. victrix	51,710	51,070 (98.76%)	640 (1.24%)	21	44 (0.09%)
E. viminalis	50,955	50,223 (98.56%)	732 (1.44%)	26	56 (0.11%)
E. virginea	51,559	50,667 (98.27%)	892 (1.73%)	35	74 (0.14%)

Supplementary Table S8. Genome orthogrouping statistics. For each genome lists the number of annotated genes, the number and proportion of genes placed within an orthogroup, the number and proportion of genes not placed within an orthogroup, the number and proportion of genes placed within a species-specific orthogroup, and the number of paralogues for each species.

Number of species	33
Number of genes	1,761,851
Number of genes in orthogroups	1,726,511
Number of unassigned genes	35,340
Percentage of genes in orthogroups	97.99%
Percentage of unassigned genes	2.01%
Number of orthogroups	68,245
Number of species-specific orthogroups	1,822
Number of genes in species-specific orthogroups	4,551
Percentage of genes in species-specific orthogroups	0.26%
Mean orthogroup size	25.3

Supplementary Table S9. Orthogrouping summary statistics.

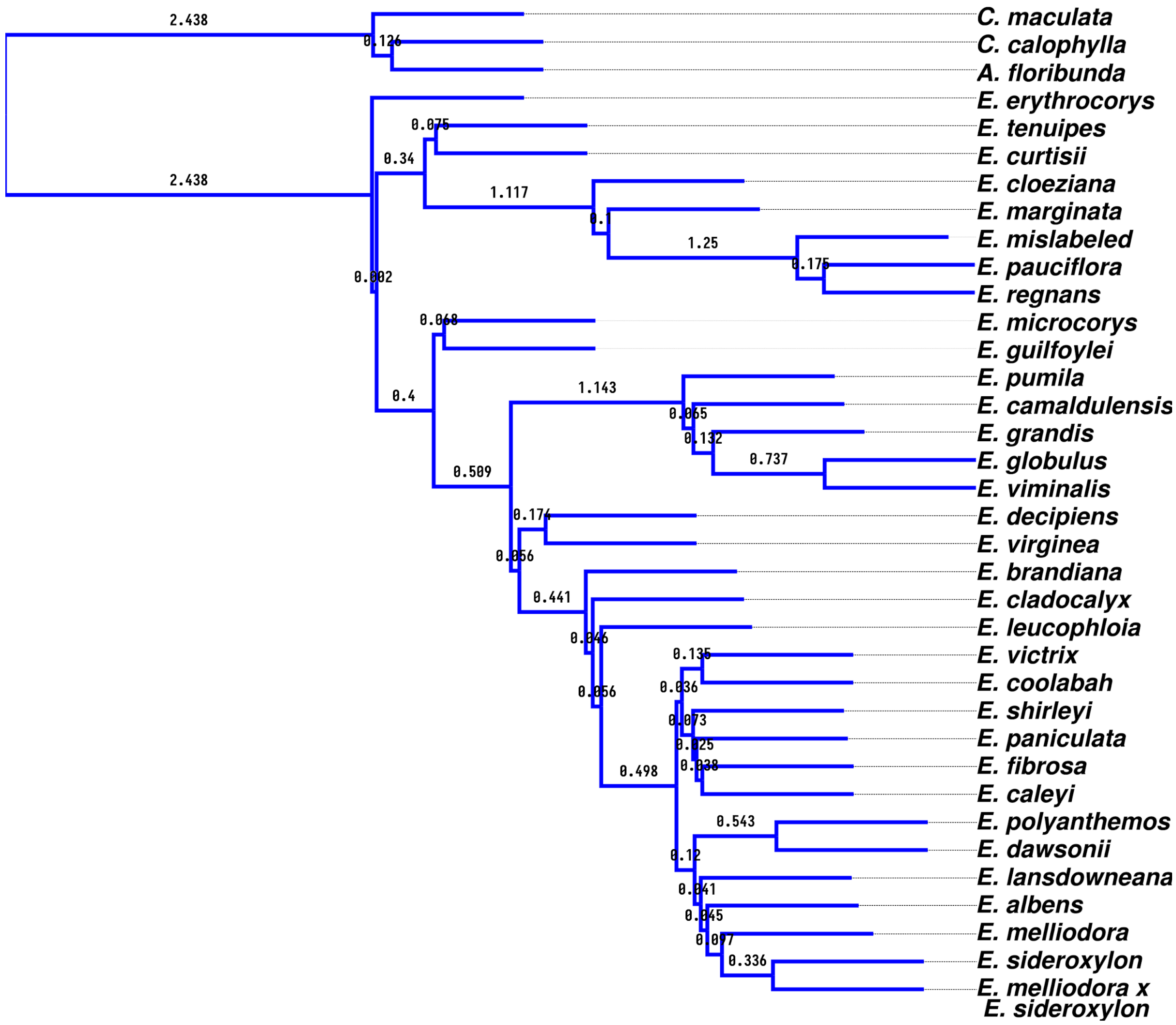

Supplementary Figure S7. Maximum likelihood species phylogeny estimated from 1,674 BUSCO genes. Branch lengths are in coalescent units, as calculated by Astral III. Astral doesn't calculate branch lengths for tips, as tips are a single genome.

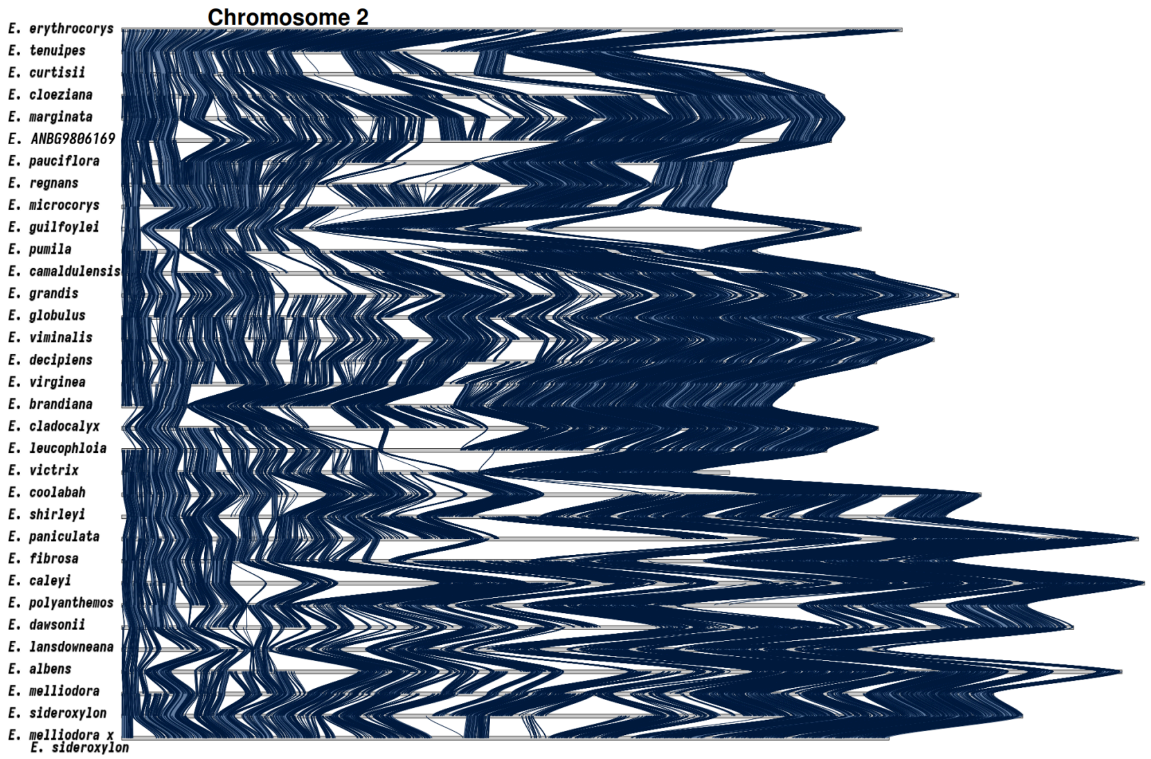

Supplementary Figure S8. Synteny karyotype of chromosome 2. The blue ribbons between karyotypes indicate the presence of syntenic sequences between species pairs. In all other regions synteny has become lost. Synteny is lost to either rearrangements (inverted, translocated, or duplicated), sequence divergence, loss or gain.

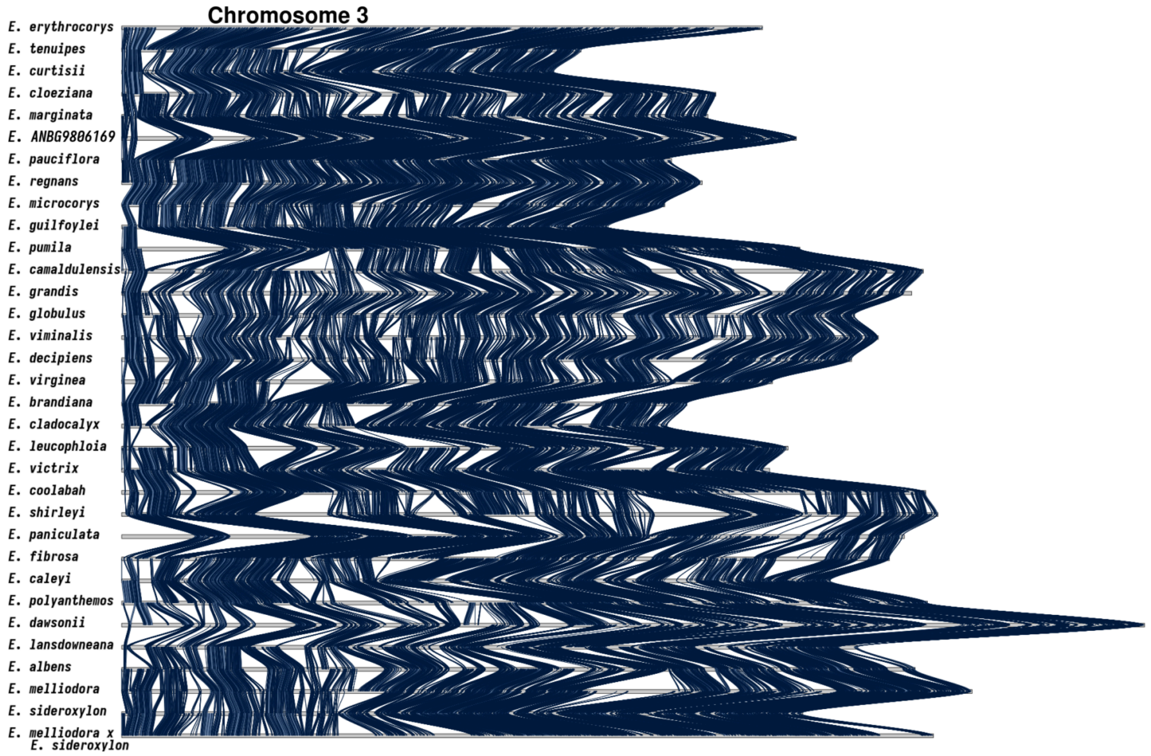

Supplementary Figure S9. Synteny karyotype of chromosome 3. The blue ribbons between karyotypes indicate the presence of syntenic sequences between species pairs. In all other regions synteny has become lost. Synteny is lost to either rearrangements (inverted, translocated, or duplicated), sequence divergence, loss or gain.

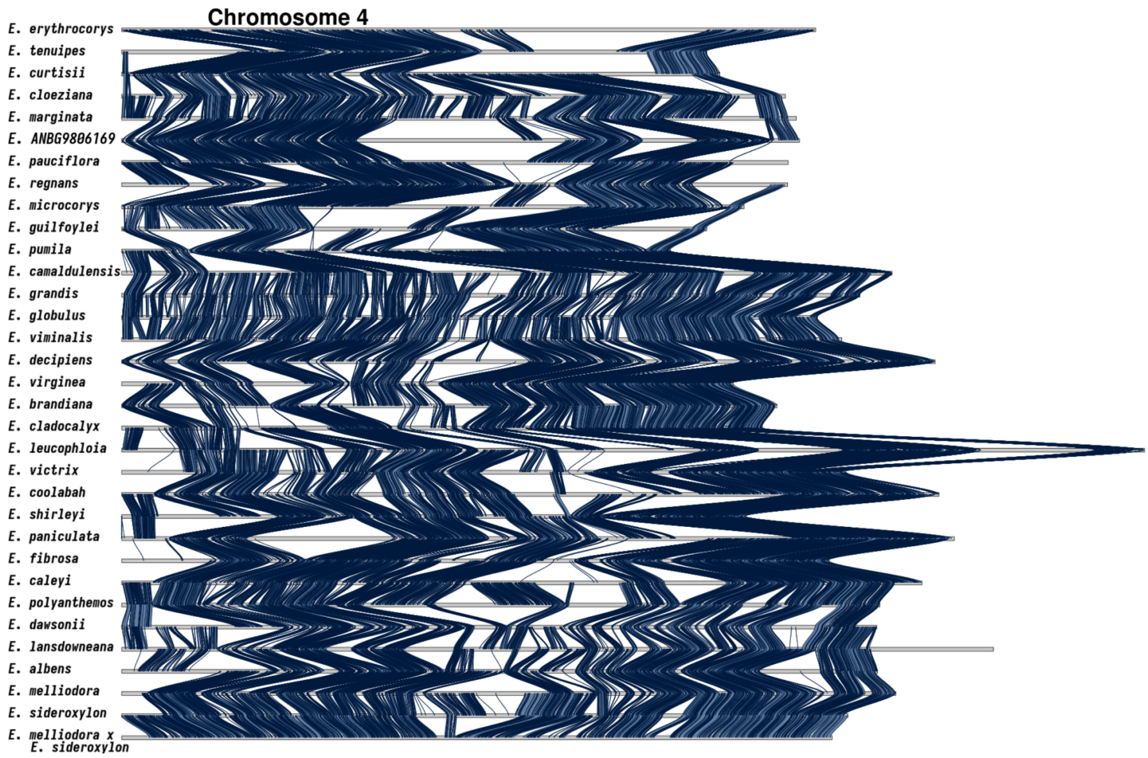

Supplementary Figure S10. Synteny karyotype of chromosome 4. The blue ribbons between karyotypes indicate the presence of syntenic sequences between species pairs. In all other regions synteny has become lost. Synteny is lost to either rearrangements (inverted, translocated, or duplicated), sequence divergence, loss or gain.

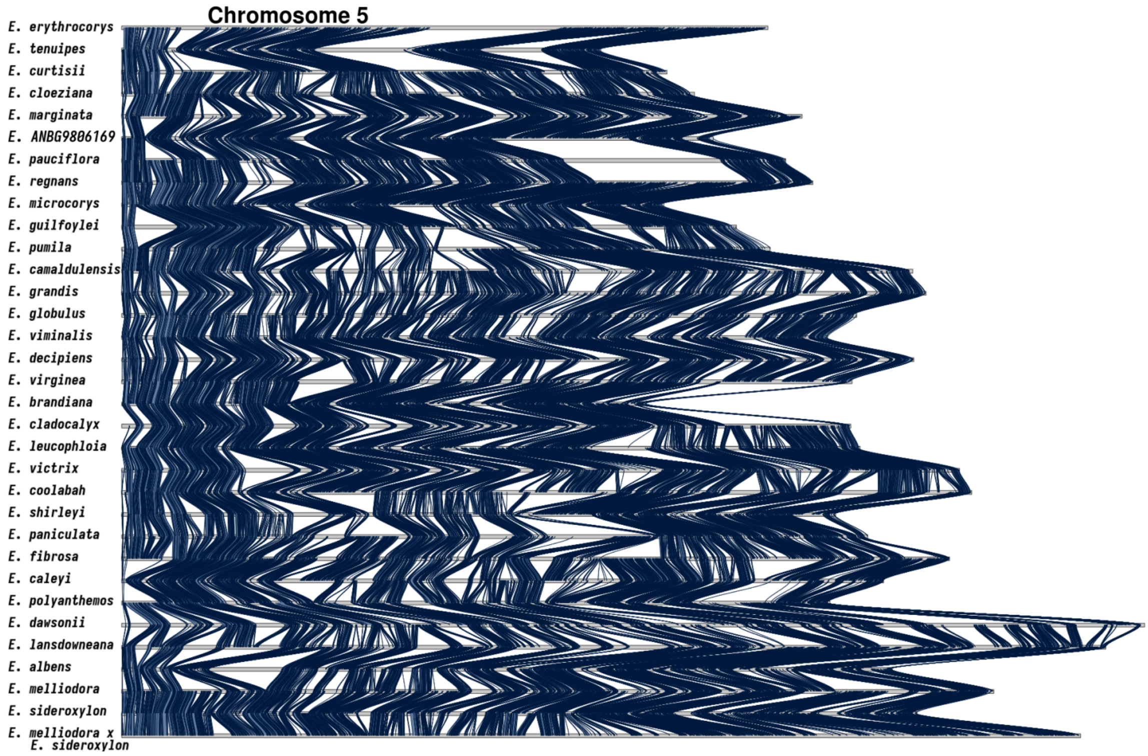

Supplementary Figure S11. Synteny karyotype of chromosome 5. The blue ribbons between karyotypes indicate the presence of syntenic sequences between species pairs. In all other regions synteny has become lost. Synteny is lost to either rearrangements (inverted, translocated, or duplicated), sequence divergence, loss or gain.

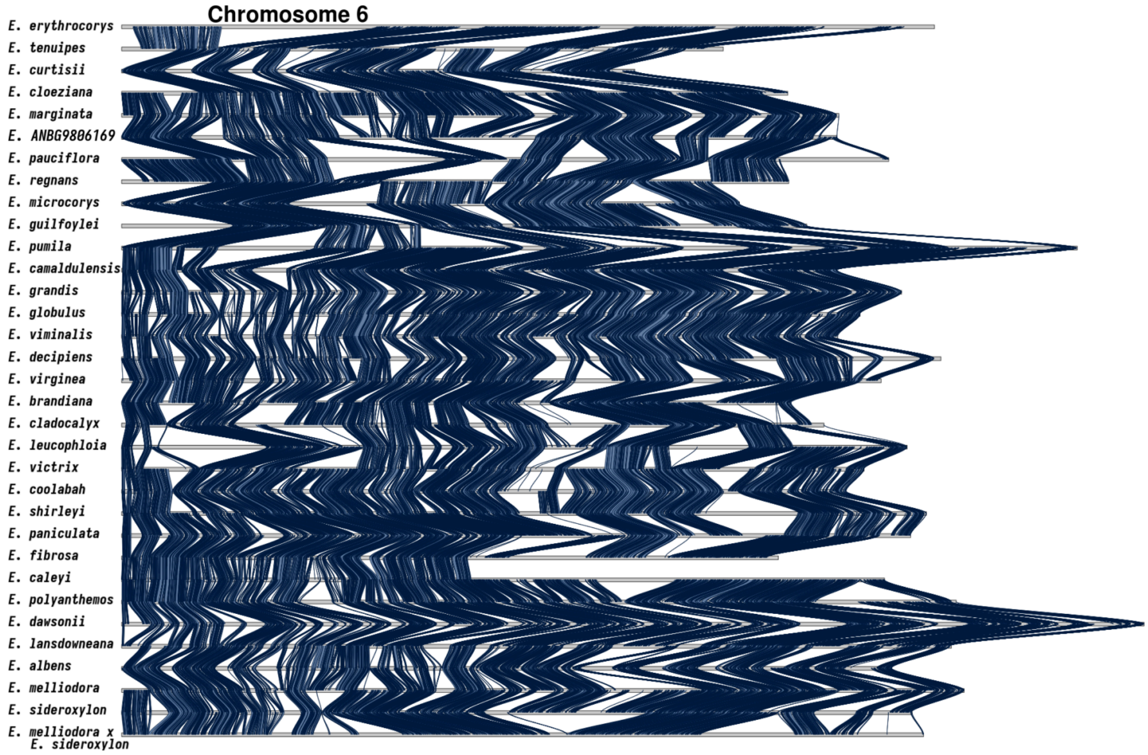

Supplementary Figure S12. Synteny karyotype of chromosome 6. The blue ribbons between karyotypes indicate the presence of syntenic sequences between species pairs. In all other regions synteny has become lost. Synteny is lost to either rearrangements (inverted, translocated, or duplicated), sequence divergence, loss or gain.

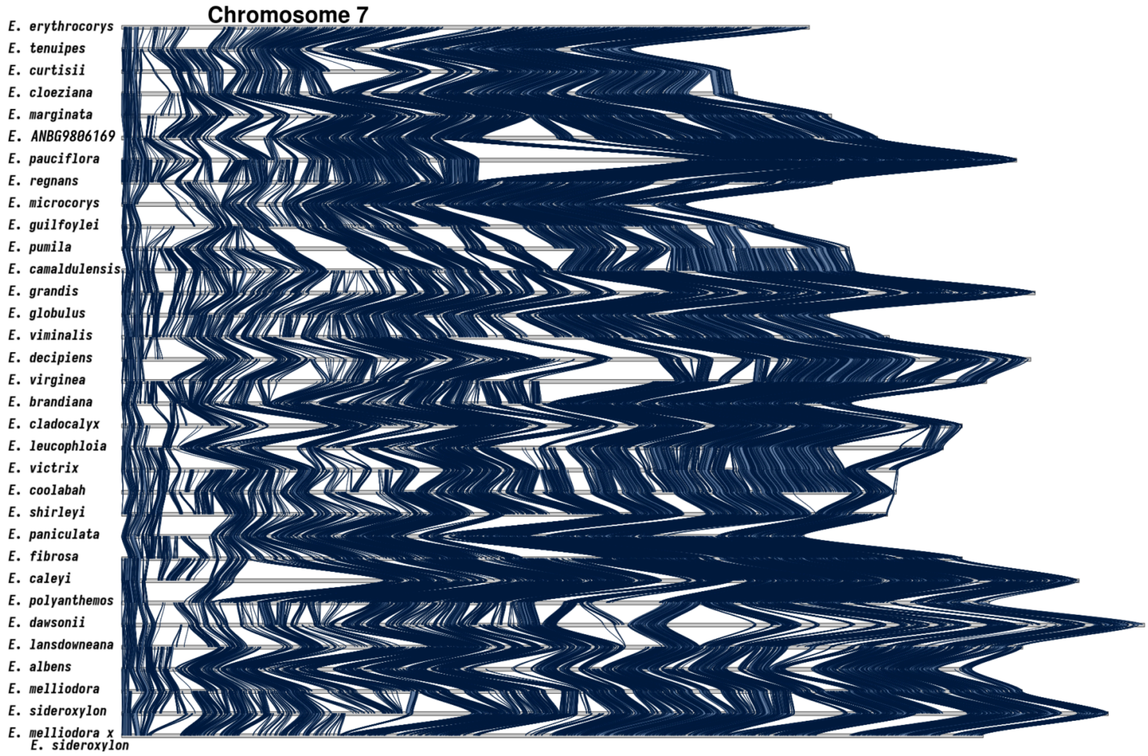

Supplementary Figure S13. Synteny karyotype of chromosome 7. The blue ribbons between karyotypes indicate the presence of syntenic sequences between species pairs. In all other regions synteny has become lost. Synteny is lost to either rearrangements (inverted, translocated, or duplicated), sequence divergence, loss or gain.

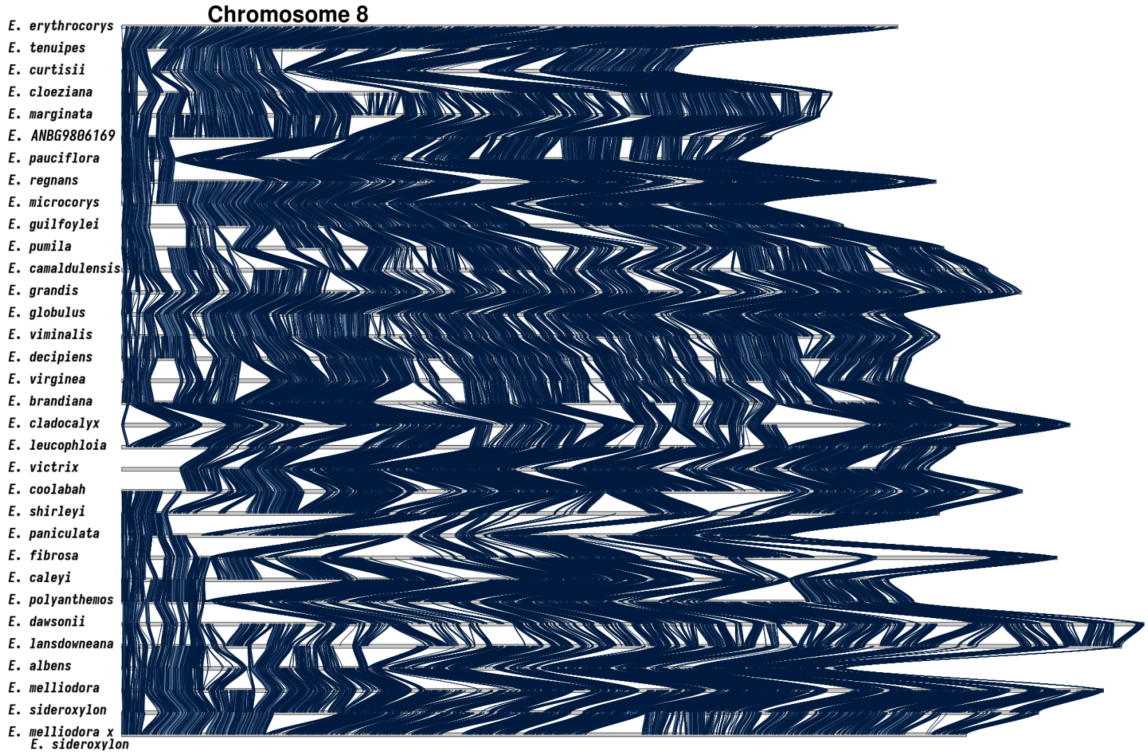

Supplementary Figure S14. Synteny karyotype of chromosome 8. The blue ribbons between karyotypes indicate the presence of syntenic sequences between species pairs. In all other regions synteny has become lost. Synteny is lost to either rearrangements (inverted, translocated, or duplicated), sequence divergence, loss or gain.

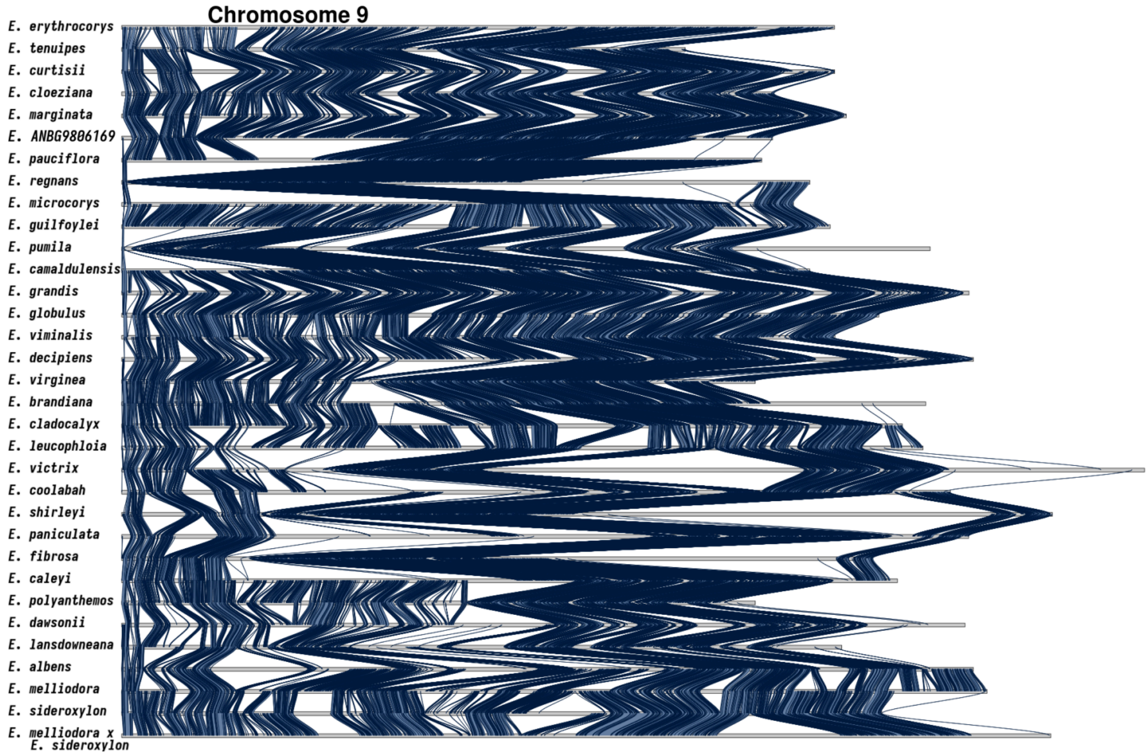

Supplementary Figure S15. Synteny karyotype of chromosome 9. The blue ribbons between karyotypes indicate the presence of syntenic sequences between species pairs. In all other regions synteny has become lost. Synteny is lost to either rearrangements (inverted, translocated, or duplicated), sequence divergence, loss or gain.

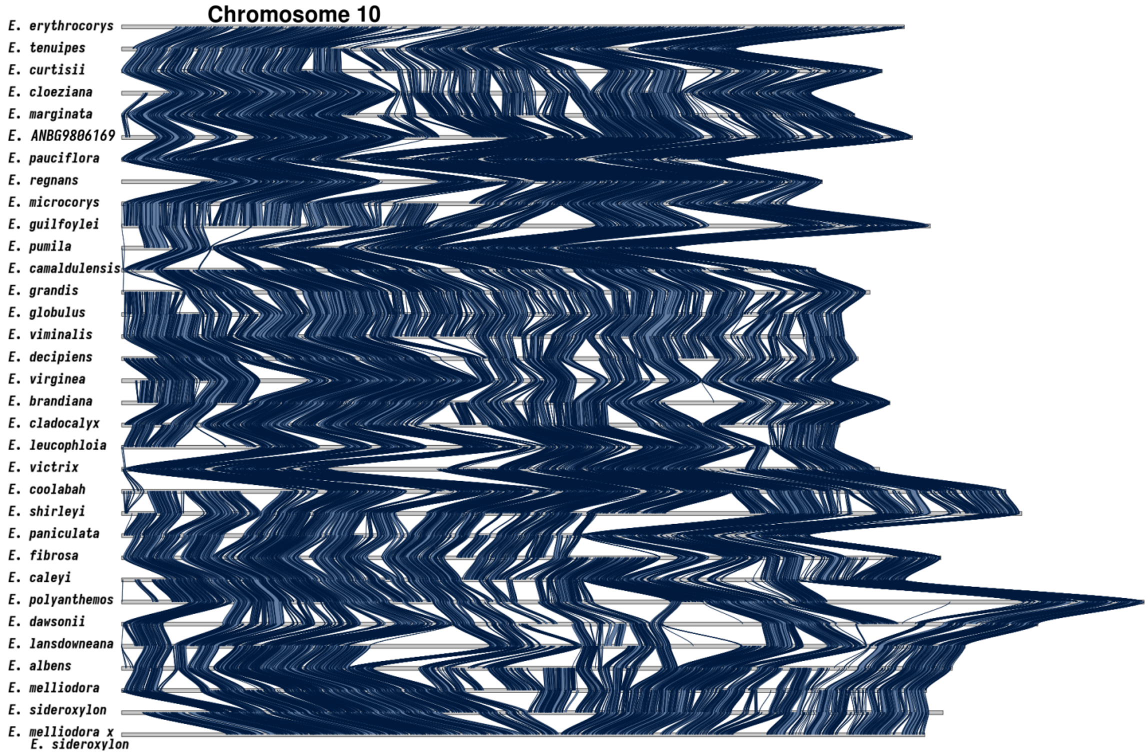

Supplementary Figure S16. Synteny karyotype of chromosome 10. The blue ribbons between karyotypes indicate the presence of syntenic sequences between species pairs. In all other regions synteny has become lost. Synteny is lost to either rearrangements (inverted, translocated, or duplicated), sequence divergence, loss or gain.

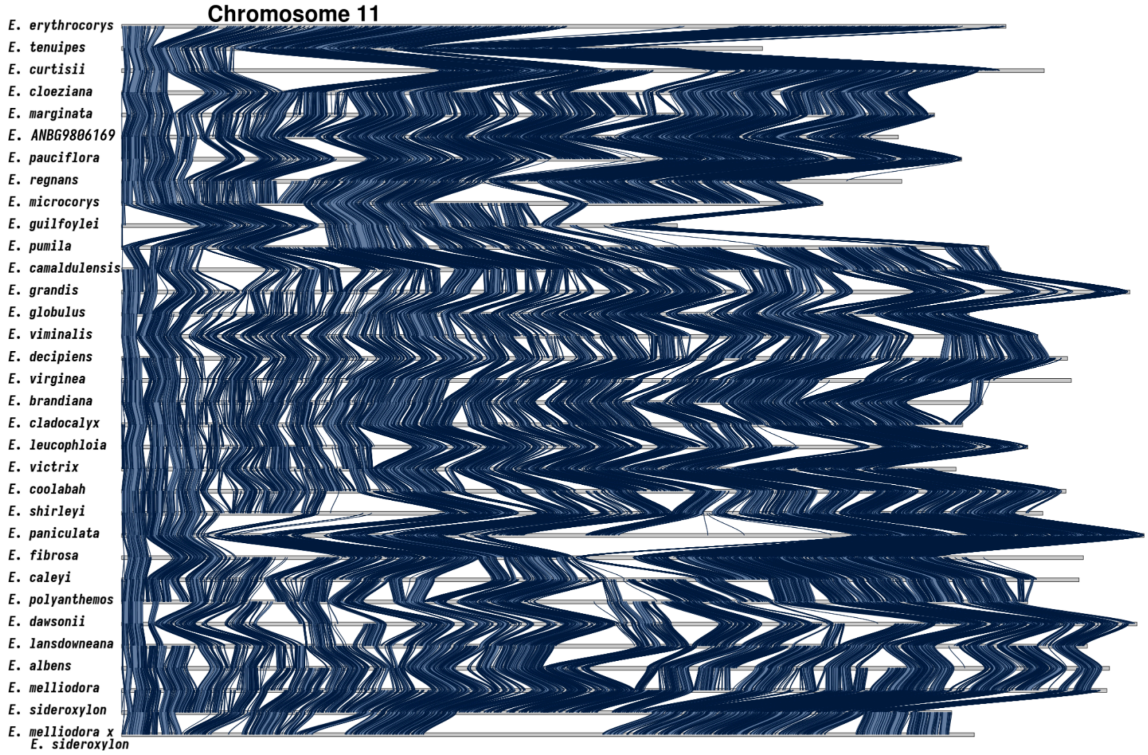

Supplementary Figure S17. Synteny karyotype of chromosome 11. The blue ribbons between karyotypes indicate the presence of syntenic sequences between species pairs. In all other regions synteny has become lost. Synteny is lost to either rearrangements (inverted, translocated, or duplicated), sequence divergence, loss or gain.

	albens	brandiana	caleyi	camaldulensis	cladocalyx	cloeziana	coolabah	curtisii	dawsonii	decipiens	erythrocorys
albens		39.2%	43.1%	35.8%	36.8%	32.0%	43.8%	28.9%	37.5%	36.9%	28.4%
brandiana	46.7%		49.2%	45.5%	46.9%	41.8%	50.2%	36.6%	42.5%	48.2%	36.5%
caleyi	44.6%	42.6%		38.6%	40.5%	35.0%	47.5%	29.5%	40.3%	39.9%	29.3%
camaldulensis	38.7%	41.5%	40.6%		39.1%	38.4%	42.1%	32.9%	35.3%	42.3%	32.1%
cladocalyx	41.3%	44.0%	43.8%	40.3%		36.7%	44.2%	31.4%	37.9%	41.5%	30.9%
cloeziana	39.7%	43.8%	42.1%	43.8%	40.6%		44.1%	40.5%	36.5%	43.6%	37.2%
coolabah	44.0%	42.4%	46.3%	39.1%	39.7%	35.9%		31.0%	40.1%	40.2%	30.4%
curtisii	39.0%	41.6%	38.9%	41.3%	38.5%	44.4%	41.8%		34.7%	41.3%	37.8%
dawsonii	32.3%	30.7%	33.7%	28.0%	29.2%	25.7%	34.4%	22.1%		28.8%	21.3%
decipiens	38.1%	41.9%	40.1%	40.3%	38.4%	36.5%	41.3%	31.3%	34.7%		31.7%
erythrocorys	32.9%	36.1%	33.1%	34.7%	32.5%	35.3%	35.2%	32.8%	28.7%	35.8%	
fibrosa	43.9%	42.7%	45.2%	38.2%	38.5%	34.2%	47.0%	30.3%	39.2%	39.2%	29.5%
globulus	41.2%	44.1%	43.0%	53.3%	41.6%	40.2%	44.7%	34.5%	37.7%	44.8%	33.9%
grandis	34.6%	36.9%	35.8%	45.4%	34.6%	34.0%	37.6%	29.0%	32.0%	37.8%	28.4%
guilfoylei	38.0%	41.2%	40.5%	40.8%	38.7%	39.8%	41.8%	36.2%	34.9%	41.9%	35.3%
lansdowneana	40.9%	38.8%	40.7%	34.1%	36.6%	31.1%	42.1%	27.1%	36.7%	35.5%	26.4%
leucophloia	40.5%	41.6%	42.0%	38.1%	39.0%	34.5%	44.2%	30.9%	36.0%	39.4%	29.5%
marginata	37.8%	41.6%	40.0%	40.9%	37.9%	50.8%	41.6%	38.6%	34.1%	41.5%	35.9%
melliodora	45.5%	41.5%	46.2%	38.1%	39.0%	34.5%	46.9%	30.0%	40.2%	39.3%	29.1%
melliodora x sideroxylon	40.7%	40.7%	40.7%	40.7%	40.7%	40.7%	40.7%	40.7%	40.7%	40.7%	40.7%
microcorys	46.6%	50.5%	49.7%	49.7%	46.9%	48.6%	50.4%	42.6%	42.4%	50.5%	41.9%
ANBG9806169	35.1%	38.0%	36.5%	38.1%	35.0%	48.9%	38.4%	36.4%	31.4%	38.0%	33.6%
paniculata	42.0%	39.8%	44.2%	36.5%	37.5%	33.2%	44.6%	29.0%	38.2%	37.9%	28.2%
pauciflora	34.2%	38.9%	34.9%	36.4%	34.1%	46.5%	36.8%	34.7%	30.3%	37.3%	32.7%
polyanthemos	43.1%	40.8%	45.4%	36.6%	38.1%	32.9%	45.0%	29.1%	45.1%	38.2%	28.3%
pumila	37.2%	40.0%	38.3%	47.9%	37.8%	36.6%	40.2%	30.9%	34.4%	40.4%	30.5%
regnans	34.8%	39.2%	37.2%	37.8%	35.8%	46.7%	38.2%	36.6%	31.8%	37.9%	34.1%
shirleyi	40.8%	39.7%	44.2%	35.8%	37.3%	33.0%	44.8%	28.5%	37.2%	37.4%	28.1%
sideroxylon	45.9%	43.1%	47.6%	38.6%	41.3%	35.4%	47.7%	30.9%	41.6%	40.3%	30.5%
tenuipes	45.6%	49.7%	48.0%	49.0%	46.0%	52.5%	49.3%	48.0%	40.8%	50.2%	46.0%
victrix	45.6%	45.6%	47.2%	40.7%	41.6%	36.9%	52.5%	32.8%	40.6%	41.6%	32.3%
viminalis	39.9%	42.5%	41.5%	51.4%	39.9%	39.1%	43.4%	33.8%	36.6%	43.2%	32.8%
virginea	41.4%	45.5%	43.4%	43.7%	41.0%	38.5%	44.7%	34.1%	37.2%	48.0%	32.6%

	fibrosa	globulus	grandis	guilfoylei	lansdowneana	leucophloia	marginata	melliodora	melliodora x sideroxylon	microcorys	ANBG9806169
albens	42.7%	37.0%	34.9%	30.5%	42.6%	37.6%	32.5%	47.5%	43.7%	34.9%	30.1%
brandiana	49.7%	47.3%	44.6%	39.0%	48.3%	46.4%	42.5%	51.7%	48.3%	44.8%	38.8%
caleyi	45.4%	39.8%	37.4%	33.4%	43.7%	40.3%	35.5%	49.9%	45.3%	38.2%	32.3%
camaldulensis	40.3%	51.9%	49.8%	35.4%	38.6%	38.6%	38.4%	43.2%	40.0%	40.4%	35.5%
cladocalyx	41.9%	41.8%	39.2%	34.6%	42.7%	40.7%	36.5%	45.6%	42.7%	39.2%	33.6%
cloeziana	41.1%	45.1%	42.7%	39.4%	40.0%	39.9%	54.1%	44.4%	40.9%	45.2%	52.1%
coolabah	45.9%	40.2%	38.2%	33.9%	44.1%	41.4%	36.2%	49.2%	44.7%	37.9%	33.2%
curtisii	39.9%	42.3%	39.9%	39.4%	38.0%	38.9%	45.3%	42.4%	40.7%	43.5%	42.2%
dawsonii	32.8%	29.2%	27.9%	24.2%	33.0%	28.9%	25.1%	36.2%	33.7%	27.4%	23.3%
decipiens	39.4%	41.5%	39.5%	34.4%	38.2%	37.9%	36.7%	42.4%	39.9%	38.6%	33.5%
erythrocorys	33.3%	36.0%	33.7%	33.3%	32.0%	32.4%	36.3%	35.3%	33.5%	36.7%	33.3%
fibrosa		39.3%	37.2%	32.7%	44.2%	39.2%	34.4%	48.6%	45.3%	36.9%	32.5%
globulus	42.6%		53.7%	37.2%	41.1%	41.1%	40.5%	45.4%	42.1%	42.5%	37.0%
grandis	35.7%	47.7%		31.4%	34.1%	34.0%	33.6%	38.3%	35.5%	35.9%	31.0%
guilfoylei	39.9%	41.9%	39.9%		38.0%	38.0%	40.6%	42.0%	39.8%	45.6%	38.0%
lansdowneana	41.4%	35.5%	33.1%	29.4%		36.2%	31.3%	44.5%	41.8%	33.3%	28.7%
leucophloia	40.9%	39.6%	37.1%	32.7%	40.7%		35.0%	44.7%	41.6%	37.1%	31.9%
marginata	39.0%	42.4%	39.8%	37.8%	37.8%	38.0%		41.9%	39.2%	42.6%	49.0%
melliodora	45.2%	39.0%	37.1%	32.3%	44.5%	39.9%	34.7%		35.9%	35.9%	35.9%
melliodora x sideroxylon	40.7%	40.7%	40.7%	40.7%	40.7%	40.7%	40.7%	40.7%		40.7%	40.7%
microcorys	48.2%	51.7%	48.9%	48.9%	46.5%	46.5%	49.0%	51.5%	47.7%		45.0%
ANBG9806169	37.2%	39.1%	36.8%	35.6%	35.1%	34.8%	49.2%	38.9%	36.5%	39.5%	
paniculata	44.5%	37.6%	35.5%	30.4%	41.9%	38.4%	33.0%	46.8%	43.2%	34.7%	30.1%
pauciflora	37.6%	37.6%	35.3%	35.7%	34.5%	33.9%	47.0%	37.7%	36.1%	38.7%	50.7%
polyanthemos	43.7%	37.8%	36.0%	31.7%	43.7%	38.4%	33.3%	48.5%	44.9%	36.5%	30.4%
pumila	38.4%	48.7%	46.1%	33.2%	38.0%	37.0%	36.4%	41.2%	37.7%	38.3%	33.9%
regnans	39.3%	39.4%	36.3%	36.5%	35.3%	35.4%	47.2%	38.5%	36.9%	39.8%	52.0%
shirleyi	45.1%	37.2%	35.3%	32.1%	41.3%	38.3%	33.2%	45.4%	42.3%	35.4%	30.6%
sideroxylon	46.8%	40.1%	37.7%	33.9%	46.6%	40.8%	35.8%	53.1%	53.4%	38.3%	32.6%
tenuipes	49.1%	50.7%	47.6%	45.9%	44.8%	45.0%	53.1%	50.6%	47.7%	51.2%	49.3%
victrix	50.6%	41.9%	39.4%	36.2%	46.2%	42.6%	37.5%	50.2%	46.1%	40.1%	34.5%
viminalis	41.1%	59.0%	52.2%	36.3%	39.6%	39.7%	39.2%	43.8%	40.8%	41.4%	36.2%
virginea	43.6%	44.8%	42.2%	37.3%	41.7%	41.4%	39.1%	45.8%	42.6%	41.8%	35.8%

	paniculata	*pauciflora*	*polyanthemos*	*pumila*	*regnans*	*shirleyi*	*sideroxylon*	*tenuipes*	*victrix*	*viminalis*	*virginea*
albens	40.6%	28.7%	42.7%	32.6%	29.2%	40.1%	44.8%	31.4%	41.8%	36.7%	36.6%
brandiana	46.1%	38.6%	48.2%	41.9%	39.0%	46.6%	50.2%	40.3%	49.8%	46.5%	47.9%
caleyi	44.2%	30.1%	46.5%	34.6%	32.2%	44.9%	47.9%	34.0%	44.7%	39.3%	39.5%
camaldulensis	38.3%	33.1%	39.3%	45.6%	34.5%	38.2%	40.9%	36.6%	40.4%	51.2%	41.9%
cladocalyx	40.5%	31.9%	42.2%	37.0%	33.6%	41.0%	45.0%	35.1%	42.6%	41.0%	40.5%
cloeziana	39.8%	48.4%	40.4%	39.9%	48.6%	40.2%	42.4%	44.3%	42.0%	45.0%	43.0%
coolabah	43.3%	31.1%	44.8%	35.4%	32.5%	44.3%	46.7%	34.2%	48.4%	40.0%	39.7%
curtisii	38.3%	39.4%	38.9%	37.0%	41.5%	37.9%	40.8%	44.4%	40.9%	42.4%	41.5%
dawsonii	31.8%	21.8%	38.6%	26.0%	23.1%	31.5%	34.9%	24.2%	32.0%	28.9%	28.3%
decipiens	38.1%	32.2%	39.1%	36.6%	32.8%	38.2%	40.6%	35.4%	39.3%	40.9%	43.6%
erythrocorys	31.5%	31.9%	32.4%	31.3%	33.2%	32.2%	34.4%	36.8%	34.2%	35.5%	34.0%
fibrosa	44.4%	32.2%	44.4%	34.7%	33.6%	45.7%	46.8%	34.5%	47.6%	38.7%	39.6%
globulus	40.5%	34.8%	41.7%	47.4%	36.4%	40.7%	43.5%	38.1%	42.7%	60.4%	44.1%
grandis	33.9%	28.9%	35.1%	39.8%	29.8%	34.3%	36.3%	32.1%	35.7%	47.5%	36.9%
guilfoylei	36.7%	37.3%	39.2%	36.5%	38.0%	39.3%	40.9%	39.1%	41.3%	41.8%	41.7%
lansdowneana	38.8%	27.8%	41.6%	31.9%	28.4%	39.1%	43.5%	29.4%	40.5%	34.9%	35.3%
leucophloia	39.8%	30.4%	40.7%	34.7%	31.9%	40.5%	42.7%	33.1%	41.9%	39.0%	39.1%
marginata	37.2%	45.8%	38.4%	37.2%	46.2%	38.1%	40.5%	41.8%	39.6%	42.1%	40.7%
melliodora	35.9%	35.9%	35.9%	35.9%	35.9%	35.9%	35.9%	35.9%	35.9%	35.9%	35.9%
melliodora x sideroxylon	40.7%	40.7%	40.7%	40.7%	40.7%	40.7%	40.7%	40.7%	40.7%	40.7%	40.7%
microcorys	44.8%	43.1%	48.2%	45.3%	44.6%	46.7%	49.6%	46.7%	49.1%	51.2%	49.9%
ANBG9806169	34.1%	49.6%	35.0%	35.0%	50.9%	35.3%	37.0%	39.4%	36.9%	39.2%	37.5%
paniculata		29.8%	43.5%	32.8%	28.4%	41.7%	44.2%	31.6%	40.5%	37.3%	37.2%
pauciflora	34.5%		35.9%	33.3%	54.0%	34.9%	36.7%	38.5%	38.4%	37.0%	37.2%
polyanthemos	42.5%	30.5%		33.5%	30.6%	41.9%	46.5%	32.1%	43.2%	37.7%	37.9%
pumila	36.4%	31.4%	37.9%		33.4%	36.9%	39.2%	34.3%	38.2%	48.3%	39.3%
regnans	32.8%	54.0%	36.1%	35.4%		36.7%	37.6%	40.9%	38.7%	38.6%	38.2%
shirleyi	41.1%	29.5%	42.2%	32.8%	31.1%		43.1%	32.9%	44.3%	36.7%	37.1%
sideroxylon	43.8%	31.8%	47.3%	35.4%	32.5%	43.6%		34.4%	45.6%	39.3%	39.5%
tenuipes	44.8%	47.2%	46.1%	44.2%	50.2%	47.3%	48.7%		48.9%	50.1%	48.0%
victrix	42.8%	35.1%	46.8%	36.7%	35.6%	47.6%	48.6%	36.9%		41.1%	41.6%
viminalis	39.3%	33.6%	40.6%	46.1%	34.9%	39.5%	41.7%	36.9%	41.0%		42.6%
virginea	40.9%	34.6%	42.5%	39.2%	35.6%	41.5%	43.7%	36.8%	43.1%	44.4%	

Supplementary Tables S10. Matrix of pairwise shared synteny. Shows the proportion of the genome that is syntenic for all genome pairs. Read down the genome list (far left column) and extend across until the comparison genome is found. As genomes are different lengths the proportion that species X has syntenic to species Y is different to the proportion that species Y has syntenic to species X.

	albens	brandiana	caleyi	camaldulensis	cladocalyx	cloeziana	coolabah	curtisii	dawsonii	decipiens	erythrocorys
**albens**		35.2%	39.5%	34.5%	40.0%	22.9%	36.3%	26.8%	45.8%	34.5%	20.9%
**brandiana**	33.1%		31.6%	28.2%	32.3%	18.5%	28.3%	24.9%	38.8%	29.1%	19.1%
**caleyi**	37.9%	32.1%		33.4%	36.6%	21.4%	34.1%	28.0%	43.5%	33.5%	22.6%
**camaldulensis**	32.6%	27.6%	31.3%		31.3%	18.3%	27.1%	24.1%	36.9%	27.7%	20.4%
**cladocalyx**	39.2%	33.1%	36.7%	32.0%		21.4%	33.9%	27.9%	43.1%	33.6%	22.6%
**cloeziana**	23.2%	18.4%	21.7%	18.4%	21.3%		18.3%	27.4%	26.1%	19.9%	20.6%
**coolabah**	36.6%	30.6%	35.5%	29.1%	34.9%	18.7%		23.7%	40.9%	30.2%	19.5%
**curtisii**	28.2%	25.4%	29.6%	25.7%	28.8%	27.7%	24.6%		32.0%	26.9%	24.5%
**dawsonii**	48.3%	42.0%	46.9%	40.2%	45.1%	27.5%	43.4%	30.3%		40.8%	25.3%
**decipiens**	34.2%	29.9%	32.3%	29.0%	32.7%	20.2%	28.7%	26.1%	37.6%		18.6%
**erythrocorys**	22.6%	19.2%	22.7%	20.2%	22.1%	20.2%	18.9%	22.2%	24.3%	18.7%	
**fibrosa**	40.3%	33.6%	40.1%	34.6%	39.9%	22.8%	36.0%	27.4%	44.9%	35.1%	21.8%
**globulus**	30.9%	26.3%	30.0%	28.6%	29.2%	17.6%	25.1%	23.6%	34.8%	26.7%	18.3%
**grandis**	36.1%	31.6%	35.8%	35.2%	34.8%	21.6%	30.8%	26.3%	38.3%	32.4%	21.0%
**guilfoylei**	32.4%	30.1%	30.8%	29.4%	31.5%	27.8%	27.6%	32.5%	35.5%	30.1%	26.4%
**lansdowneana**	43.4%	36.6%	42.9%	36.9%	40.6%	24.3%	39.2%	28.4%	47.0%	37.5%	23.7%
**leucophloia**	39.8%	34.9%	38.9%	33.5%	37.3%	23.2%	34.9%	27.1%	43.3%	33.3%	23.1%
**marginata**	23.8%	19.6%	22.7%	20.2%	23.2%	30.4%	19.3%	28.1%	27.2%	20.3%	20.6%
**melliodora**	37.9%	32.3%	35.5%	31.1%	36.0%	18.9%	32.4%	24.6%	42.6%	31.7%	20.0%
**melliodora x sideroxylon**	22.9%	22.9%	22.9%	22.9%	22.9%	22.9%	22.9%	22.9%	22.9%	22.9%	22.9%
**microcorys**	25.9%	21.5%	22.8%	21.6%	25.0%	19.4%	20.9%	27.4%	30.2%	23.1%	21.6%
**ANBG9806169**	25.2%	22.7%	25.4%	23.0%	25.2%	35.0%	21.7%	29.9%	28.1%	23.2%	23.4%
**paniculata**	42.2%	36.9%	41.4%	36.3%	41.6%	25.0%	37.8%	29.2%	46.0%	35.8%	22.9%
**pauciflora**	28.4%	22.3%	28.5%	26.2%	28.3%	40.3%	24.8%	33.1%	31.7%	26.0%	24.9%
**polyanthemos**	40.1%	34.2%	37.8%	34.2%	39.1%	22.9%	35.9%	26.6%	42.7%	33.8%	22.3%
**pumila**	36.4%	32.1%	35.8%	35.1%	35.1%	22.7%	31.0%	28.5%	39.0%	33.2%	23.6%
**regnans**	26.9%	22.9%	24.9%	23.4%	24.6%	39.2%	22.1%	29.9%	28.6%	24.5%	21.9%
**shirleyi**	42.8%	35.8%	40.4%	35.8%	39.9%	23.6%	37.7%	29.1%	45.7%	35.3%	24.4%
**sideroxylon**	38.1%	31.7%	35.5%	32.4%	34.8%	20.3%	33.2%	24.7%	42.2%	32.0%	19.6%
**tenuipes**	24.3%	19.0%	22.7%	20.4%	23.0%	22.1%	19.0%	30.4%	27.7%	19.7%	19.7%
**victrix**	37.1%	29.9%	36.2%	31.1%	35.8%	20.3%	31.8%	24.6%	42.5%	31.7%	19.7%
**viminalis**	31.0%	26.8%	30.2%	29.1%	29.7%	17.4%	25.9%	23.1%	34.4%	27.1%	19.1%
**virginea**	34.0%	28.6%	32.7%	29.4%	33.4%	21.9%	28.5%	27.3%	38.8%	31.5%	23.1%

	fibrosa	globulus	grandis	guilfoylei	lansdowneana	leucophloia	marginata	melliodora	melliodora x sideroxylon	microcorys	ANBG9806169
albens	41.0%	32.5%	37.0%	32.4%	41.4%	41.0%	23.1%	36.1%	41.0%	27.4%	23.7%
brandiana	31.5%	25.7%	30.5%	30.3%	32.5%	34.5%	18.8%	27.7%	33.2%	22.6%	21.8%
caleyi	39.3%	31.5%	36.0%	31.3%	39.8%	39.8%	22.5%	32.8%	38.5%	25.0%	24.1%
camaldulensis	32.8%	28.7%	33.7%	29.1%	33.1%	32.8%	19.2%	27.4%	33.3%	22.9%	21.6%
cladocalyx	39.9%	30.3%	35.2%	32.3%	38.2%	38.2%	22.8%	34.0%	39.2%	26.5%	23.9%
cloeziana	22.4%	17.0%	21.1%	27.7%	22.6%	23.4%	28.6%	17.2%	24.2%	19.9%	32.2%
coolabah	37.1%	27.8%	32.2%	28.4%	37.7%	36.9%	19.5%	31.3%	37.8%	23.7%	21.2%
curtisii	28.2%	24.5%	28.4%	33.3%	29.0%	28.7%	27.9%	24.0%	28.8%	27.5%	29.4%
dawsonii	48.6%	38.6%	41.7%	36.5%	48.7%	47.0%	28.7%	45.3%	48.5%	32.6%	29.1%
decipiens	35.0%	28.4%	32.4%	30.9%	35.1%	33.7%	20.5%	29.8%	33.8%	25.7%	23.1%
erythrocorys	22.2%	18.7%	21.9%	25.1%	23.0%	22.3%	19.0%	18.7%	23.2%	21.1%	22.1%
fibrosa		32.7%	37.3%	33.9%	39.6%	41.9%	23.7%	35.4%	40.0%	28.3%	23.6%
globulus	31.0%		31.3%	29.0%	32.0%	30.1%	17.7%	25.8%	32.6%	22.2%	20.6%
grandis	36.2%	33.0%		32.0%	37.2%	36.0%	23.0%	31.8%	37.4%	27.4%	24.9%
guilfoylei	32.2%	28.5%	31.8%		33.1%	33.6%	27.7%	28.4%	32.9%	28.5%	28.0%
lansdowneana	42.3%	35.0%	39.2%	34.4%		43.2%	25.2%	39.8%	43.8%	30.1%	26.1%
leucophloia	40.8%	30.8%	35.9%	33.2%	39.5%		23.2%	34.7%	39.5%	27.7%	25.6%
marginata	23.4%	18.3%	23.1%	28.8%	24.0%	23.9%		18.9%	24.9%	21.4%	34.1%
melliodora	37.2%	29.9%	34.0%	29.9%	38.2%	36.7%	20.1%		39.6%	39.6%	39.6%
melliodora x sideroxylon	22.9%	22.9%	22.9%	22.9%	22.9%	22.9%	22.9%	22.9%		22.9%	22.9%
microcorys	25.6%	19.8%	24.8%	27.9%	26.5%	26.9%	19.6%	19.4%	26.4%		23.1%
ANBG9806169	24.0%	21.3%	25.9%	28.6%	25.4%	26.4%	34.7%	21.1%	26.7%	24.0%	
paniculata	41.7%	34.5%	38.2%	35.0%	42.2%	41.0%	25.8%	37.0%	42.3%	30.6%	26.8%
pauciflora	25.7%	24.8%	28.0%	30.9%	27.9%	27.8%	39.2%	22.7%	27.2%	26.8%	41.1%
polyanthemos	40.3%	32.4%	36.2%	31.9%	39.7%	39.7%	23.0%	35.1%	39.5%	26.1%	25.1%
pumila	36.1%	33.0%	37.5%	35.0%	36.2%	36.0%	23.5%	31.7%	38.2%	28.3%	24.2%
regnans	23.1%	21.1%	25.9%	30.4%	26.3%	26.3%	38.3%	21.4%	26.5%	24.9%	39.3%
shirleyi	39.6%	33.7%	37.8%	32.7%	41.8%	41.4%	24.6%	38.0%	41.9%	29.7%	26.1%
sideroxylon	37.3%	29.4%	33.9%	29.9%	37.4%	37.7%	20.9%	31.7%	34.8%	24.1%	22.9%
tenuipes	21.7%	18.7%	23.2%	29.2%	24.8%	25.2%	21.7%	17.0%	22.6%	21.5%	24.2%
victrix	32.9%	29.4%	33.8%	29.4%	36.5%	38.1%	20.4%	32.0%	37.6%	23.9%	22.7%
viminalis	32.3%	26.5%	31.5%	28.4%	31.8%	30.4%	17.8%	26.1%	32.5%	22.0%	20.5%
virginea	33.1%	28.3%	32.9%	31.6%	34.2%	33.7%	22.2%	28.8%	34.8%	26.5%	24.6%

	paniculata	pauciflora	polyanthemos	pumila	regnans	shirleyi	sideroxylon	tenuipes	victrix	viminalis	virginea
albens	43.3%	27.8%	40.3%	37.4%	24.2%	42.7%	38.4%	24.1%	38.6%	32.7%	36.1%
brandiana	36.0%	20.3%	31.5%	32.4%	20.4%	34.4%	29.2%	20.3%	28.8%	27.1%	29.9%
caleyi	41.8%	26.9%	36.3%	37.0%	22.6%	39.8%	34.7%	23.7%	36.8%	31.4%	34.7%
camaldulensis	34.8%	23.6%	31.9%	35.4%	20.6%	34.5%	29.9%	21.8%	30.0%	29.0%	30.2%
cladocalyx	41.3%	25.8%	38.4%	36.3%	23.0%	39.7%	34.4%	23.3%	36.0%	30.8%	35.4%
cloeziana	24.3%	35.9%	22.2%	22.5%	35.8%	23.0%	20.1%	23.4%	20.0%	17.3%	21.9%
coolabah	39.2%	24.0%	36.1%	32.8%	20.4%	38.4%	33.5%	19.8%	34.0%	27.7%	31.4%
curtisii	30.6%	32.7%	27.9%	30.3%	29.6%	30.5%	26.1%	31.7%	25.9%	24.3%	28.0%
dawsonii	49.4%	31.9%	46.9%	41.6%	28.1%	48.6%	46.3%	28.7%	46.4%	38.2%	42.3%
decipiens	35.2%	23.8%	32.4%	34.3%	22.6%	34.1%	31.1%	20.8%	31.9%	29.2%	34.6%
erythrocorys	23.5%	23.0%	22.0%	23.2%	19.5%	22.2%	19.9%	19.5%	20.1%	18.1%	22.2%
fibrosa	42.1%	24.8%	39.8%	37.8%	21.7%	39.1%	37.0%	23.2%	34.2%	33.8%	35.6%
globulus	33.5%	23.5%	30.3%	34.1%	19.6%	32.4%	28.0%	19.6%	28.7%	26.0%	29.3%
grandis	38.3%	26.2%	35.3%	39.5%	23.8%	37.6%	33.8%	23.6%	34.6%	33.7%	34.9%
guilfoylei	35.6%	29.4%	31.5%	34.8%	28.5%	32.0%	29.3%	29.2%	29.1%	28.1%	30.8%
lansdowneana	45.6%	27.7%	42.0%	39.2%	25.5%	44.6%	40.8%	26.5%	40.6%	35.6%	38.7%
leucophloia	41.0%	26.7%	38.6%	36.7%	24.3%	40.9%	36.3%	26.0%	38.5%	31.5%	35.4%
marginata	26.4%	37.5%	23.1%	24.5%	36.6%	24.4%	21.3%	24.4%	21.2%	18.9%	22.9%
melliodora	39.6%	39.6%	39.6%	39.6%	39.6%	39.6%	39.6%	39.6%	39.6%	39.6%	39.6%
melliodora x sideroxylon	22.9%	22.9%	22.9%	22.9%	22.9%	22.9%	22.9%	22.9%	22.9%	22.9%	22.9%
microcorys	29.8%	24.3%	24.1%	27.4%	22.6%	27.0%	22.2%	22.7%	22.7%	20.0%	24.7%
ANBG9806169	27.5%	39.3%	25.4%	25.1%	38.4%	26.0%	23.6%	26.9%	23.3%	21.3%	25.0%
paniculata		26.5%	40.3%	39.5%	30.6%	43.3%	39.9%	25.6%	42.8%	34.7%	38.6%
pauciflora	27.2%		24.6%	29.4%	37.7%	28.4%	25.8%	28.2%	23.5%	25.6%	28.6%
polyanthemos	41.4%	23.1%		37.5%	23.3%	41.0%	37.0%	24.3%	37.8%	32.4%	34.9%
pumila	38.5%	27.8%	36.2%		24.5%	37.7%	33.9%	25.9%	34.5%	33.7%	35.9%
regnans	31.8%	36.9%	24.6%	25.9%		26.5%	23.4%	25.3%	22.2%	22.1%	25.8%
shirleyi	44.2%	27.7%	40.6%	39.0%	25.2%		40.2%	25.0%	37.8%	34.6%	37.2%
sideroxylon	40.4%	23.2%	36.0%	34.9%	21.3%	39.9%		21.4%	35.4%	30.4%	33.7%
tenuipes	25.6%	25.4%	22.9%	25.8%	22.3%	23.3%	20.4%		19.7%	18.7%	23.5%
victrix	41.7%	21.8%	35.2%	35.0%	20.4%	35.8%	33.5%	20.6%		30.2%	32.4%
viminalis	33.4%	23.5%	30.0%	34.2%	19.9%	32.2%	28.6%	19.7%	28.8%		29.3%
virginea	36.2%	26.0%	32.2%	35.5%	23.5%	34.7%	31.0%	24.7%	31.1%	28.1%	

Supplementary Tables S11. Matrix of pairwise rearrangements. Shows the proportion of the genome that is rearranged for all genome pairs. Read down the genome list (far left column) and extend across until the comparison genome is found. As genomes are different lengths the proportion that species X has rearranged to species Y is different to the proportion that species Y has rearranged to species X.

	albens	brandiana	caleyi	camaldulensis	cladocalyx	cloeziana	coolabah	curtisii	dawsonii	decipiens	erythrocorys
albens		74.4%	82.6%	70.3%	76.8%	54.9%	80.1%	55.6%	83.3%	71.4%	49.3%
brandiana	79.9%		80.7%	73.8%	79.1%	60.4%	78.6%	61.5%	81.3%	77.3%	55.6%
caleyi	82.5%	74.7%		71.9%	77.1%	56.4%	81.6%	57.6%	83.8%	73.4%	51.9%
camaldulensis	71.4%	69.0%	71.9%		70.3%	56.7%	69.2%	57.0%	72.1%	70.0%	52.5%
cladocalyx	80.5%	77.1%	80.5%	72.3%		58.1%	78.1%	59.3%	80.9%	75.0%	53.4%
cloeziana	63.0%	62.2%	63.7%	62.2%	61.9%		62.4%	68.0%	62.6%	63.5%	57.8%
coolabah	80.6%	73.0%	81.8%	68.2%	74.6%	54.6%		54.7%	81.1%	70.3%	49.9%
curtisii	67.2%	67.0%	68.5%	67.0%	67.3%	72.1%	66.4%		66.7%	68.2%	62.4%
dawsonii	80.6%	72.7%	80.7%	68.3%	74.2%	53.2%	77.8%	52.4%		69.6%	46.7%
decipiens	72.3%	71.8%	72.4%	69.3%	71.1%	56.7%	70.0%	57.4%	72.3%		50.4%
erythrocorys	55.4%	55.3%	55.8%	54.9%	54.5%	55.4%	54.2%	55.0%	53.0%	54.5%	
fibrosa	84.2%	76.3%	85.3%	72.8%	78.4%	57.0%	83.0%	57.7%	84.1%	74.3%	51.4%
globulus	72.1%	70.4%	73.0%	81.9%	70.9%	57.8%	69.8%	58.1%	72.4%	71.6%	52.2%
grandis	70.7%	68.5%	71.7%	80.6%	69.4%	55.6%	68.4%	55.4%	70.3%	70.3%	49.5%
guilfoylei	70.4%	71.3%	71.4%	70.2%	70.1%	67.6%	69.4%	68.7%	70.3%	72.0%	61.7%
lansdowneana	84.3%	75.5%	83.6%	71.0%	77.2%	55.4%	81.3%	55.5%	83.7%	73.0%	50.1%
leucophloia	80.3%	76.5%	80.8%	71.6%	76.2%	57.7%	79.1%	58.0%	79.4%	72.7%	52.7%
marginata	61.6%	61.2%	62.7%	61.1%	61.1%	81.2%	60.9%	66.7%	61.2%	61.9%	56.5%
melliodora	83.4%	73.8%	81.7%	69.2%	74.9%	53.4%	79.3%	54.6%	82.7%	70.9%	49.1%
melliodora x sideroxylon	63.5%	63.5%	63.5%	63.5%	63.5%	63.5%	63.5%	63.5%	63.5%	63.5%	63.5%
microcorys	72.5%	72.0%	72.5%	71.3%	72.0%	68.0%	71.3%	70.0%	72.7%	73.6%	63.5%
ANBG9806169	60.3%	60.7%	61.9%	61.1%	60.2%	84.0%	60.1%	66.3%	59.5%	61.2%	56.9%
paniculata	84.1%	76.7%	85.6%	72.8%	79.1%	58.2%	82.3%	58.2%	84.3%	73.7%	51.1%
pauciflora	62.6%	61.2%	63.4%	62.7%	62.4%	86.7%	61.6%	67.8%	62.0%	63.3%	57.5%
polyanthemos	83.2%	75.0%	83.3%	70.8%	77.2%	55.7%	81.0%	55.7%	87.8%	72.0%	50.5%
pumila	73.6%	72.1%	74.1%	83.1%	72.9%	59.3%	71.2%	59.4%	73.4%	73.6%	54.1%
regnans	61.7%	62.1%	62.2%	61.2%	60.4%	85.9%	60.2%	66.5%	60.4%	62.4%	56.0%
shirleyi	83.6%	75.5%	84.6%	71.6%	77.1%	56.6%	82.5%	57.6%	82.9%	72.7%	52.5%
sideroxylon	84.0%	74.8%	83.0%	71.0%	76.1%	55.7%	80.9%	55.7%	83.9%	72.2%	50.1%
tenuipes	70.0%	68.7%	70.7%	69.4%	68.9%	74.6%	68.3%	78.4%	68.5%	69.9%	65.7%
victrix	82.7%	75.5%	83.5%	71.7%	77.4%	57.1%	84.3%	57.5%	83.1%	73.3%	52.0%
viminalis	70.9%	69.4%	71.7%	80.4%	69.6%	56.5%	69.3%	56.8%	71.0%	70.3%	51.9%
virginea	75.3%	74.2%	76.1%	73.1%	74.4%	60.4%	73.2%	61.4%	76.0%	79.5%	55.7%

	fibrosa	*globulus*	*grandis*	*guilfoylei*	*lansdowneana*	*leucophloia*	*marginata*	*melliodora*	*melliodora x sideroxylon*	*microcorys*	*ANBG9806169*
albens	83.7%	69.6%	72.0%	63.0%	84.0%	78.6%	55.6%	83.6%	84.7%	62.3%	53.8%
brandiana	81.2%	73.0%	75.1%	69.3%	80.8%	80.9%	61.3%	79.4%	81.6%	67.4%	60.6%
caleyi	84.8%	71.4%	73.5%	64.6%	83.5%	80.0%	57.9%	82.7%	83.8%	63.3%	56.3%
camaldulensis	73.2%	80.6%	83.5%	64.6%	71.7%	71.4%	57.6%	70.6%	73.3%	63.2%	57.1%
cladocalyx	81.8%	72.1%	74.4%	66.9%	80.9%	78.9%	59.3%	79.6%	81.8%	65.7%	57.5%
cloeziana	63.4%	62.1%	63.8%	67.1%	62.6%	63.3%	82.7%	61.5%	65.1%	65.1%	84.3%
coolabah	83.0%	68.0%	70.4%	62.3%	81.7%	78.2%	55.7%	80.6%	82.4%	61.6%	54.5%
curtisii	68.1%	66.9%	68.3%	72.7%	67.0%	67.6%	73.2%	66.4%	69.4%	71.0%	71.7%
dawsonii	81.5%	67.8%	69.5%	60.7%	81.7%	75.8%	53.8%	81.5%	82.2%	60.0%	52.4%
decipiens	74.4%	70.0%	71.8%	65.3%	73.3%	71.6%	57.2%	72.2%	73.7%	64.3%	56.7%
erythrocorys	55.5%	54.7%	55.5%	58.5%	55.0%	54.8%	55.4%	54.0%	56.7%	57.8%	55.4%
fibrosa		72.0%	74.5%	66.6%	83.8%	81.1%	58.1%	84.0%	85.3%	65.1%	56.1%
globulus	73.6%		85.0%	66.2%	73.1%	71.2%	58.2%	71.2%	74.7%	64.7%	57.6%
grandis	71.9%	80.7%		63.4%	71.2%	70.1%	56.6%	70.1%	72.9%	63.3%	55.8%
guilfoylei	72.1%	70.4%	71.7%		71.1%	71.5%	68.3%	70.4%	72.7%	74.1%	66.0%
lansdowneana	83.7%	70.4%	72.4%	63.8%		79.5%	56.5%	84.3%	85.7%	63.4%	54.8%
leucophloia	81.8%	70.4%	72.9%	65.8%	80.2%		58.2%	79.4%	81.1%	64.8%	57.5%
marginata	62.4%	60.7%	62.8%	66.7%	61.8%	61.9%		60.8%	64.1%	63.9%	83.1%
melliodora	82.4%	69.0%	71.2%	62.2%	82.7%	76.7%	54.8%		75.5%	75.5%	75.5%
melliodora x sideroxylon	63.5%	63.5%	63.5%	63.5%	63.5%	63.5%	63.5%	63.5%		63.5%	63.5%
microcorys	73.7%	71.5%	73.8%	76.9%	73.0%	73.4%	68.6%	70.9%	74.1%		68.1%
ANBG9806169	61.2%	60.5%	62.7%	64.2%	60.4%	61.2%	83.9%	59.9%	63.1%	63.5%	
paniculata	86.2%	72.1%	73.8%	65.4%	84.0%	79.4%	58.8%	83.9%	85.5%	65.2%	56.9%
pauciflora	63.3%	62.4%	63.3%	66.6%	62.4%	61.7%	86.2%	60.4%	63.3%	65.5%	91.9%
polyanthemos	84.0%	70.2%	72.2%	63.6%	83.4%	78.1%	56.3%	83.5%	84.4%	62.6%	55.5%
pumila	74.5%	81.6%	83.6%	68.2%	74.3%	73.0%	59.9%	72.9%	75.9%	66.6%	58.1%
regnans	62.4%	60.6%	62.3%	66.9%	61.7%	61.8%	85.5%	59.9%	63.4%	64.7%	91.3%
shirleyi	84.7%	70.9%	73.1%	64.7%	83.1%	79.7%	57.8%	83.3%	84.2%	65.1%	56.7%
sideroxylon	84.1%	69.6%	71.6%	63.8%	84.0%	78.5%	56.7%	84.8%	88.2%	62.4%	55.6%
tenuipes	70.8%	69.3%	70.8%	75.1%	69.5%	70.2%	74.8%	67.6%	70.3%	72.7%	73.5%
victrix	83.5%	71.3%	73.2%	65.6%	82.7%	80.7%	57.9%	82.2%	83.7%	64.0%	57.2%
viminalis	73.3%	85.5%	83.7%	64.7%	71.4%	70.1%	57.0%	70.0%	73.3%	63.3%	56.7%
virginea	76.7%	73.1%	75.1%	68.9%	75.9%	75.1%	61.3%	74.5%	77.4%	68.3%	60.4%

	paniculata	***pauciflora***	***polyanthemos***	***pumila***	***regnans***	***shirleyi***	***sideroxylon***	***tenuipes***	***victrix***	***viminalis***	***virginea***
albens	84.0%	56.5%	83.0%	69.9%	53.4%	82.8%	83.2%	55.5%	80.4%	69.3%	72.6%
brandiana	82.1%	58.8%	79.7%	74.3%	59.4%	81.0%	79.4%	60.6%	78.6%	73.6%	77.7%
caleyi	86.0%	57.0%	82.7%	71.7%	54.8%	84.7%	82.6%	57.7%	81.5%	70.7%	74.1%
camaldulensis	73.0%	56.7%	71.2%	81.0%	55.2%	72.7%	70.8%	58.4%	70.5%	80.3%	72.1%
cladocalyx	81.8%	57.7%	80.5%	73.2%	56.5%	80.6%	79.3%	58.4%	78.6%	71.8%	75.9%
cloeziana	64.1%	84.3%	62.6%	62.4%	84.5%	63.2%	62.6%	67.7%	62.0%	62.3%	64.9%
coolabah	82.5%	55.2%	80.9%	68.2%	52.9%	82.8%	80.2%	54.0%	82.4%	67.7%	71.1%
curtisii	68.8%	72.1%	66.8%	67.3%	71.1%	68.5%	66.9%	76.1%	66.8%	66.6%	69.5%
dawsonii	81.3%	53.7%	85.5%	67.6%	51.1%	80.2%	81.3%	52.9%	78.3%	67.0%	70.7%
decipiens	73.2%	56.1%	71.5%	70.9%	55.4%	72.2%	71.7%	56.2%	71.2%	70.1%	78.2%
erythrocorys	55.0%	54.9%	54.5%	54.5%	52.7%	54.5%	54.3%	56.3%	54.3%	53.6%	56.3%
fibrosa	86.5%	57.0%	84.2%	72.5%	55.4%	84.9%	83.8%	57.7%	81.8%	72.4%	75.2%
globulus	74.1%	58.3%	72.0%	81.5%	56.0%	73.1%	71.4%	57.7%	71.3%	86.5%	73.4%
grandis	72.3%	55.1%	70.3%	79.3%	53.6%	71.9%	70.2%	55.7%	70.2%	81.1%	71.8%
guilfoylei	72.4%	66.7%	70.7%	71.3%	66.6%	71.4%	70.2%	68.3%	70.4%	69.9%	72.5%
lansdowneana	84.4%	55.5%	83.6%	71.1%	54.0%	83.7%	84.3%	55.9%	81.1%	70.5%	74.0%
leucophloia	80.8%	57.1%	79.4%	71.4%	56.2%	81.5%	79.0%	59.2%	80.4%	70.5%	74.5%
marginata	63.6%	83.3%	61.5%	61.7%	82.8%	62.5%	61.9%	66.3%	60.8%	61.0%	63.5%
melliodora	75.5%	75.5%	75.5%	75.5%	75.5%	75.5%	75.5%	75.5%	75.5%	75.5%	75.5%
melliodora x sideroxylon	63.5%	63.5%	63.5%	63.5%	63.5%	63.5%	63.5%	63.5%	63.5%	63.5%	63.5%
microcorys	74.6%	67.4%	72.3%	72.8%	67.2%	73.7%	71.8%	69.4%	71.7%	71.2%	74.6%
ANBG9806169	61.5%	88.9%	60.4%	60.0%	89.3%	61.3%	60.6%	66.3%	60.2%	60.4%	62.6%
paniculata		56.3%	83.8%	72.3%	59.0%	85.0%	84.1%	57.2%	83.4%	72.0%	75.8%
pauciflora	61.8%		60.4%	62.7%	91.7%	63.3%	62.5%	66.7%	61.8%	62.6%	65.8%
polyanthemos	83.9%	53.6%		71.0%	53.9%	82.8%	83.6%	56.4%	81.0%	70.1%	72.8%
pumila	74.9%	59.2%	74.1%		57.9%	74.6%	73.1%	60.3%	72.7%	82.0%	75.3%
regnans	64.6%	90.9%	60.7%	61.4%		63.2%	61.0%	66.3%	60.9%	60.8%	64.0%
shirleyi	85.3%	57.3%	82.8%	71.9%	56.2%		83.3%	57.9%	82.1%	71.3%	74.3%
sideroxylon	84.2%	55.0%	83.2%	70.3%	53.8%	83.5%		55.8%	81.0%	69.7%	73.3%
tenuipes	70.4%	72.5%	69.0%	70.1%	72.5%	70.6%	69.1%		68.6%	68.8%	71.5%
victrix	84.5%	56.9%	82.0%	71.7%	55.9%	83.4%	82.1%	57.5%		71.3%	74.0%
viminalis	72.8%	57.1%	70.6%	80.3%	54.7%	71.7%	70.4%	56.6%	69.8%		71.9%
virginea	77.0%	60.6%	74.7%	74.7%	59.1%	76.2%	74.7%	61.5%	74.2%	72.5%	

Supplementary Tables S12. Matrix of pairwise shared sequence (synteny + rearranged). Shows the proportion of the genome that is shared for all genome pairs. Read down the genome list (far left column) and extend across until the comparison genome is found. As genomes are different lengths the proportion that species X shares with species Y is different to the proportion that species Y shares with species X.

	albens	brandiana	caleyi	camaldulensis	cladocalyx	cloeziana	coolabah	curtisii	dawsonii	decipiens	erythrocorys
albens		25.6%	17.4%	29.7%	23.2%	45.1%	19.9%	44.4%	16.7%	28.6%	50.7%
brandiana	20.1%		19.3%	26.2%	20.9%	39.6%	21.4%	38.5%	18.7%	22.7%	44.4%
caleyi	17.5%	25.3%		28.1%	22.9%	43.6%	18.4%	42.4%	16.2%	26.6%	48.1%
camaldulensis	28.6%	31.0%	28.1%		29.7%	43.3%	30.8%	43.0%	27.9%	30.0%	47.5%
cladocalyx	19.5%	22.9%	19.5%	27.7%		41.9%	21.9%	40.7%	19.1%	25.0%	46.6%
cloeziana	37.0%	37.8%	36.3%	37.8%	38.1%		37.6%	32.0%	37.4%	36.5%	42.2%
coolabah	19.4%	27.0%	18.2%	31.8%	25.4%	45.4%		45.3%	18.9%	29.7%	50.1%
curtisii	32.8%	33.0%	31.5%	33.0%	32.7%	27.9%	33.6%		33.3%	31.8%	37.6%
dawsonii	19.4%	27.3%	19.3%	31.7%	25.8%	46.8%	22.2%	47.6%		30.4%	53.3%
decipiens	27.7%	28.2%	27.6%	30.7%	28.9%	43.3%	30.0%	42.6%	27.7%		49.6%
erythrocorys	44.6%	44.7%	44.2%	45.1%	45.5%	44.6%	45.8%	45.0%	47.0%	45.5%	
fibrosa	15.8%	23.7%	14.7%	27.2%	21.6%	43.0%	17.0%	42.3%	15.9%	25.7%	48.6%
globulus	27.9%	29.6%	27.0%	18.1%	29.1%	42.2%	30.2%	41.9%	27.6%	28.4%	47.8%
grandis	29.3%	31.5%	28.3%	19.4%	30.6%	44.4%	31.6%	44.6%	29.7%	29.7%	50.5%
guilfoylei	29.6%	28.7%	28.6%	29.8%	29.9%	32.4%	30.6%	31.3%	29.7%	28.0%	38.3%
lansdowneana	15.7%	24.5%	16.4%	29.0%	22.8%	44.6%	18.7%	44.5%	16.3%	27.0%	49.9%
leucophloia	19.7%	23.5%	19.2%	28.4%	23.8%	42.3%	20.9%	42.0%	20.6%	27.3%	47.3%
marginata	38.4%	38.8%	37.3%	38.9%	38.9%	18.8%	39.1%	33.3%	38.8%	38.1%	43.5%
melliodora	16.6%	26.2%	18.3%	30.8%	25.1%	46.6%	20.7%	45.4%	17.3%	29.1%	50.9%
melliodora x sideroxylon	36.5%	36.5%	36.5%	36.5%	36.5%	36.5%	36.5%	36.5%	36.5%	36.5%	36.5%
microcorys	27.5%	28.0%	27.5%	28.7%	28.0%	32.0%	28.7%	30.0%	27.3%	26.4%	36.5%
ANBG9806169	39.7%	39.3%	38.1%	38.9%	39.8%	16.0%	39.9%	33.7%	40.5%	38.8%	43.1%
paniculata	15.9%	23.3%	14.4%	27.2%	20.9%	41.8%	17.7%	41.8%	15.7%	26.3%	48.9%
pauciflora	37.4%	38.8%	36.6%	37.3%	37.6%	13.3%	38.4%	32.2%	38.0%	36.7%	42.5%
polyanthemos	16.8%	25.0%	16.7%	29.2%	22.8%	44.3%	19.0%	44.3%	12.2%	28.0%	49.5%
pumila	26.4%	27.9%	25.9%	16.9%	27.1%	40.7%	28.8%	40.6%	26.6%	26.4%	45.9%
regnans	38.3%	37.9%	37.8%	38.8%	39.6%	14.1%	39.8%	33.5%	39.6%	37.6%	44.0%
shirleyi	16.4%	24.5%	15.4%	28.4%	22.9%	43.4%	17.5%	42.4%	17.1%	27.3%	47.5%
sideroxylon	16.0%	25.2%	17.0%	29.0%	23.9%	44.3%	19.1%	44.3%	16.1%	27.8%	49.9%
tenuipes	30.0%	31.3%	29.3%	30.6%	31.1%	25.4%	31.7%	21.6%	31.5%	30.1%	34.3%
victrix	17.3%	24.5%	16.5%	28.3%	22.6%	42.9%	15.7%	42.5%	16.9%	26.7%	48.0%
viminalis	29.1%	30.6%	28.3%	19.6%	30.4%	43.5%	30.7%	43.2%	29.0%	29.7%	48.1%
virginea	24.7%	25.8%	23.9%	26.9%	25.6%	39.6%	26.8%	38.6%	24.0%	20.5%	44.3%

	fibrosa	*globulus*	*grandis*	*guilfoylei*	*lansdowneana*	*leucophloia*	*marginata*	*melliodora*	*melliodora x sideroxylon*	*microcorys*	*ANBG9806169*
albens	16.3%	30.4%	28.0%	37.0%	16.0%	21.4%	44.4%	16.4%	15.3%	37.7%	46.2%
brandiana	18.8%	27.0%	24.9%	30.7%	19.2%	19.1%	38.7%	20.6%	18.4%	32.6%	39.4%
caleyi	15.2%	28.6%	26.5%	35.4%	16.5%	20.0%	42.1%	17.3%	16.2%	36.7%	43.7%
camaldulensis	26.8%	19.4%	16.5%	35.4%	28.3%	28.6%	42.4%	29.4%	26.7%	36.8%	42.9%
cladocalyx	18.2%	27.9%	25.6%	33.1%	19.1%	21.1%	40.7%	20.4%	18.2%	34.3%	42.5%
cloeziana	36.6%	37.9%	36.2%	32.9%	37.4%	36.7%	17.3%	38.5%	34.9%	34.9%	15.7%
coolabah	17.0%	32.0%	29.6%	37.7%	18.3%	21.8%	44.3%	19.4%	17.6%	38.4%	45.5%
curtisii	31.9%	33.1%	31.7%	27.3%	33.0%	32.4%	26.8%	33.6%	30.6%	29.0%	28.3%
dawsonii	18.5%	32.2%	30.5%	39.3%	18.3%	24.2%	46.2%	18.5%	17.8%	40.0%	47.6%
decipiens	25.6%	30.0%	28.2%	34.7%	26.7%	28.4%	42.8%	27.8%	26.3%	35.7%	43.3%
erythrocorys	44.5%	45.3%	44.5%	41.5%	45.0%	45.2%	44.6%	46.0%	43.3%	42.2%	44.6%
fibrosa		28.0%	25.5%	33.4%	16.2%	18.9%	41.9%	16.0%	14.7%	34.9%	43.9%
globulus	26.4%		15.0%	33.8%	26.9%	28.8%	41.8%	28.8%	25.3%	35.3%	42.4%
grandis	28.1%	19.3%		36.6%	28.8%	29.9%	43.4%	29.9%	27.1%	36.7%	44.2%
guilfoylei	27.9%	29.6%	28.3%		28.9%	28.5%	31.7%	29.6%	27.3%	25.9%	34.0%
lansdowneana	16.3%	29.6%	27.6%	36.2%		20.5%	43.5%	15.7%	14.3%	36.6%	45.2%
leucophloia	18.2%	29.6%	27.1%	34.2%	19.8%		41.8%	20.6%	18.9%	35.2%	42.5%
marginata	37.6%	39.3%	37.2%	33.3%	38.2%	38.1%		39.2%	35.9%	36.1%	16.9%
melliodora	17.6%	31.0%	28.8%	37.8%	17.3%	23.3%	45.2%		24.5%	24.5%	24.5%
melliodora x sideroxylon	36.5%	36.5%	36.5%	36.5%	36.5%	36.5%	36.5%	36.5%		36.5%	36.5%
microcorys	26.3%	28.5%	26.2%	23.1%	27.0%	26.6%	31.4%	29.1%	25.9%		31.9%
ANBG9806169	38.8%	39.5%	37.3%	35.8%	39.6%	38.8%	16.1%	40.1%	36.9%	36.5%	
paniculata	13.8%	27.9%	26.2%	34.6%	16.0%	20.6%	41.2%	16.1%	14.5%	34.8%	43.1%
pauciflora	36.7%	37.6%	36.7%	33.4%	37.6%	38.3%	13.8%	39.6%	36.7%	34.5%	8.1%
polyanthemos	16.0%	29.8%	27.8%	36.4%	16.6%	21.9%	43.7%	16.5%	15.6%	37.4%	44.5%
pumila	25.5%	18.4%	16.4%	31.8%	25.7%	27.0%	40.1%	27.1%	24.1%	33.4%	41.9%
regnans	37.6%	39.4%	37.7%	33.1%	38.3%	38.2%	14.5%	40.1%	36.6%	35.3%	8.7%
shirleyi	15.3%	29.1%	26.9%	35.3%	16.9%	20.3%	42.2%	16.7%	15.8%	34.9%	43.3%
sideroxylon	15.9%	30.4%	28.4%	36.2%	16.0%	21.5%	43.3%	15.2%	11.8%	37.6%	44.4%
tenuipes	29.2%	30.7%	29.2%	24.9%	30.5%	29.8%	25.2%	32.4%	29.7%	27.3%	26.5%
victrix	16.5%	28.7%	26.8%	34.4%	17.3%	19.3%	42.1%	17.8%	16.3%	36.0%	42.8%
viminalis	26.7%	14.5%	16.3%	35.3%	28.6%	29.9%	43.0%	30.0%	26.7%	36.7%	43.3%
virginea	23.3%	26.9%	24.9%	31.1%	24.1%	24.9%	38.7%	25.5%	22.6%	31.7%	39.6%

	paniculata	pauciflora	polyanthemos	pumila	regnans	shirleyi	sideroxylon	tenuipes	victrix	viminalis	virginea
albens	16.0%	43.5%	17.0%	30.1%	46.6%	17.2%	16.8%	44.5%	19.6%	30.7%	27.4%
brandiana	17.9%	41.2%	20.3%	25.7%	40.6%	19.0%	20.6%	39.4%	21.4%	26.4%	22.3%
caleyi	14.0%	43.0%	17.3%	28.3%	45.2%	15.3%	17.4%	42.3%	18.5%	29.3%	25.9%
camaldulensis	27.0%	43.3%	28.8%	19.0%	44.8%	27.3%	29.2%	41.6%	29.5%	19.7%	27.9%
cladocalyx	18.2%	42.3%	19.5%	26.8%	43.5%	19.4%	20.7%	41.6%	21.4%	28.2%	24.1%
cloeziana	35.9%	15.7%	37.4%	37.6%	15.5%	36.8%	37.4%	32.3%	38.0%	37.7%	35.1%
coolabah	17.5%	44.8%	19.1%	31.8%	47.1%	17.2%	19.8%	46.0%	17.6%	32.3%	28.9%
curtisii	31.2%	27.9%	33.2%	32.7%	28.9%	31.5%	33.1%	23.9%	33.2%	33.4%	30.5%
dawsonii	18.7%	46.3%	14.5%	32.4%	48.9%	19.8%	18.7%	47.1%	21.7%	33.0%	29.3%
decipiens	26.8%	43.9%	28.5%	29.1%	44.6%	27.8%	28.3%	43.8%	28.8%	29.9%	21.8%
erythrocorys	45.0%	45.1%	45.5%	45.5%	47.3%	45.5%	45.7%	43.7%	45.7%	46.4%	43.7%
fibrosa	13.5%	43.0%	15.8%	27.5%	44.6%	15.1%	16.2%	42.3%	18.2%	27.6%	24.8%
globulus	25.9%	41.7%	28.0%	18.5%	44.0%	26.9%	28.6%	42.3%	28.7%	13.5%	26.6%
grandis	27.7%	44.9%	29.7%	20.7%	46.4%	28.1%	29.8%	44.3%	29.8%	18.9%	28.2%
guilfoylei	27.6%	33.3%	29.3%	28.7%	33.4%	28.6%	29.8%	31.7%	29.6%	30.1%	27.5%
lansdowneana	15.6%	44.5%	16.4%	28.9%	46.0%	16.3%	15.7%	44.1%	18.9%	29.5%	26.0%
leucophloia	19.2%	42.9%	20.6%	28.6%	43.8%	18.5%	21.0%	40.8%	19.6%	29.5%	25.5%
marginata	36.4%	16.7%	38.5%	38.3%	17.2%	37.5%	38.1%	33.7%	39.2%	39.0%	36.5%
melliodora	24.5%	24.5%	24.5%	24.5%	24.5%	24.5%	24.5%	24.5%	24.5%	24.5%	24.5%
melliodora x sideroxylon	36.5%	36.5%	36.5%	36.5%	36.5%	36.5%	36.5%	36.5%	36.5%	36.5%	36.5%
microcorys	25.4%	32.6%	27.7%	27.2%	32.8%	26.3%	28.2%	30.6%	28.3%	28.8%	25.4%
ANBG9806169	38.5%	11.1%	39.6%	40.0%	10.7%	38.7%	39.4%	33.7%	39.8%	39.6%	37.4%
paniculata		43.7%	16.2%	27.7%	41.0%	15.0%	15.9%	42.8%	16.6%	28.0%	24.2%
pauciflora	38.2%		39.6%	37.3%	8.3%	36.7%	37.5%	33.3%	38.2%	37.4%	34.2%
polyanthemos	16.1%	46.4%		29.0%	46.1%	17.2%	16.4%	43.6%	19.0%	29.9%	27.2%
pumila	25.1%	40.8%	25.9%		42.1%	25.4%	26.9%	39.7%	27.3%	18.0%	24.7%
regnans	35.4%	9.1%	39.3%	38.6%		36.8%	39.0%	33.7%	39.1%	39.2%	36.0%
shirleyi	14.7%	42.7%	17.2%	28.1%	43.8%		16.7%	42.1%	17.9%	28.7%	25.7%
sideroxylon	15.8%	45.0%	16.8%	29.7%	46.2%	16.5%		44.2%	19.0%	30.3%	26.7%
tenuipes	29.6%	27.5%	31.0%	29.9%	27.5%	29.4%	30.9%		31.4%	31.2%	28.5%
victrix	15.5%	43.1%	18.0%	28.3%	44.1%	16.6%	17.9%	42.5%		28.7%	26.0%
viminalis	27.2%	42.9%	29.4%	19.7%	45.3%	28.3%	29.6%	43.4%	30.2%		28.1%
virginea	23.0%	39.4%	25.3%	25.3%	40.9%	23.8%	25.3%	38.5%	25.8%	27.5%	

Supplementary Tables S13. Matrix of pairwise unaligned. Shows the proportion of the genome that is unaligned for all genome pairs. Read down the genome list (far left column) and extend across until the comparison genome is found.

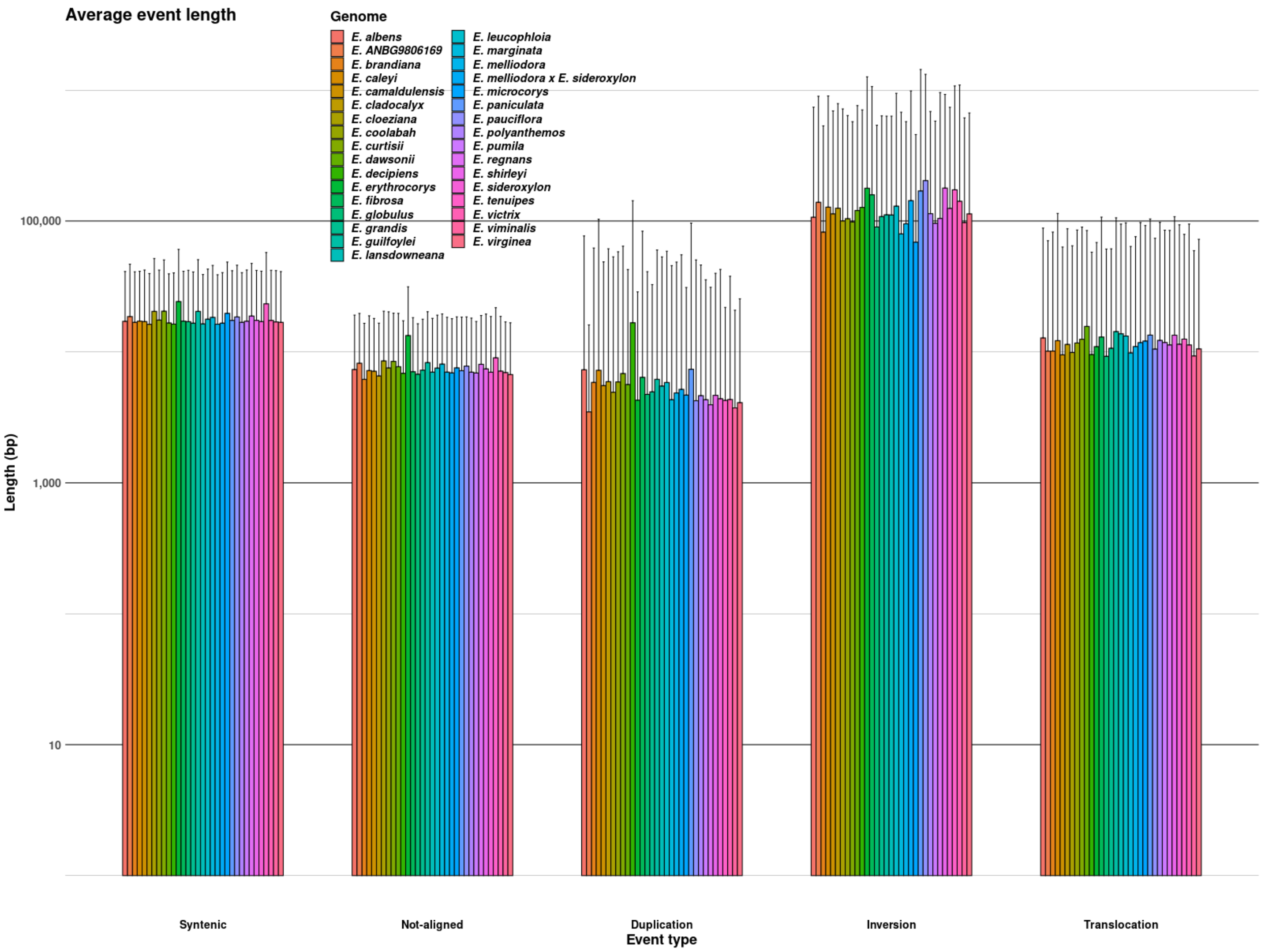

Supplementary Figure S18. Average length of syntenic, unaligned, rearrangement (combined duplications, inversions, and translocations), duplicated, inverted, and translocated regions, for all comparisons within each genome. Error bars show standard deviation of region lengths.

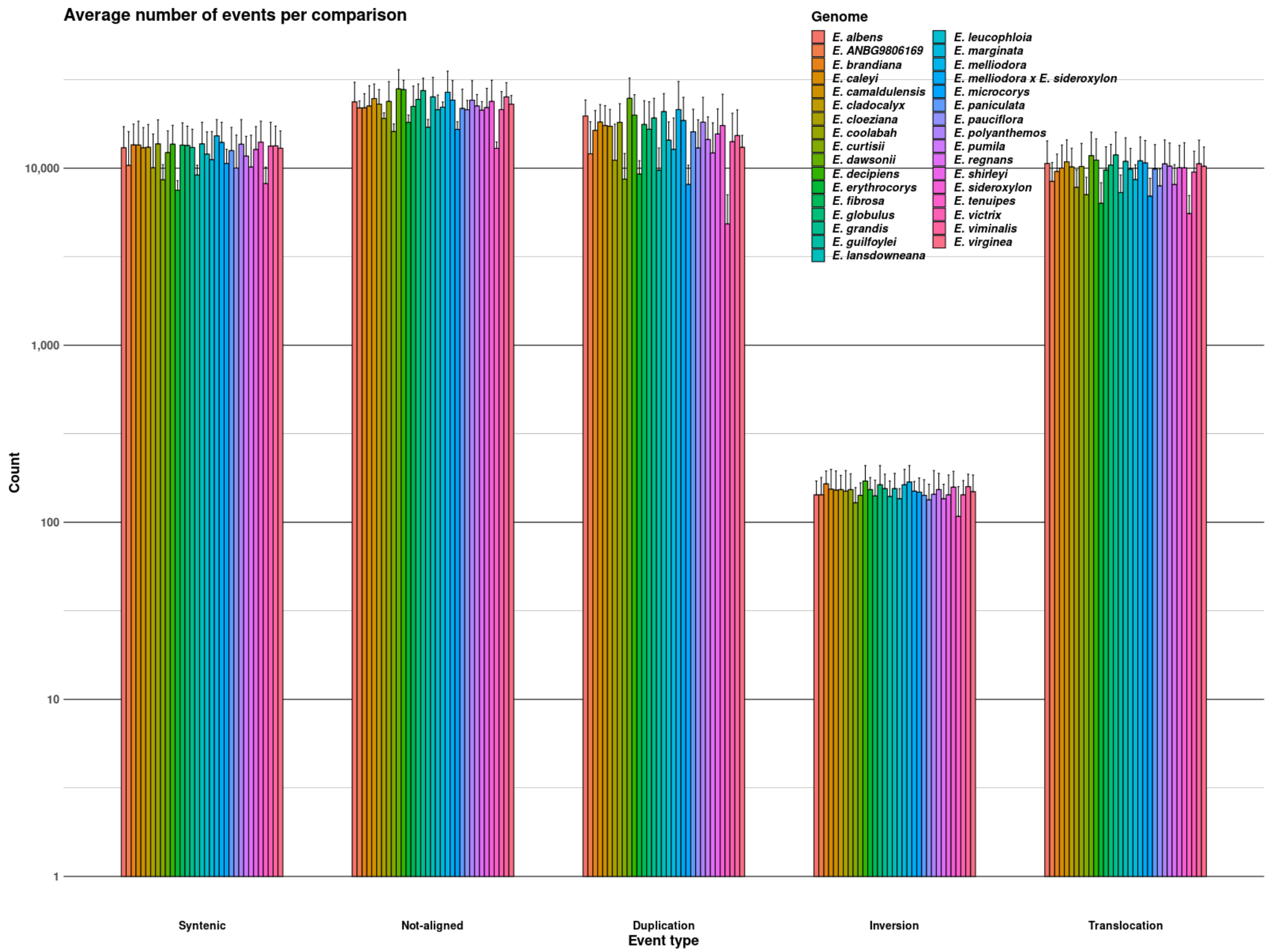

Supplementary Figure S19. Average number of syntenic, unaligned, rearrangement (combined duplications, inversions, and translocations), duplicated, inverted, and translocated regions, for all comparisons within each genome. Error bars show the minimum and maximum number of events counted within a comparison for each genome

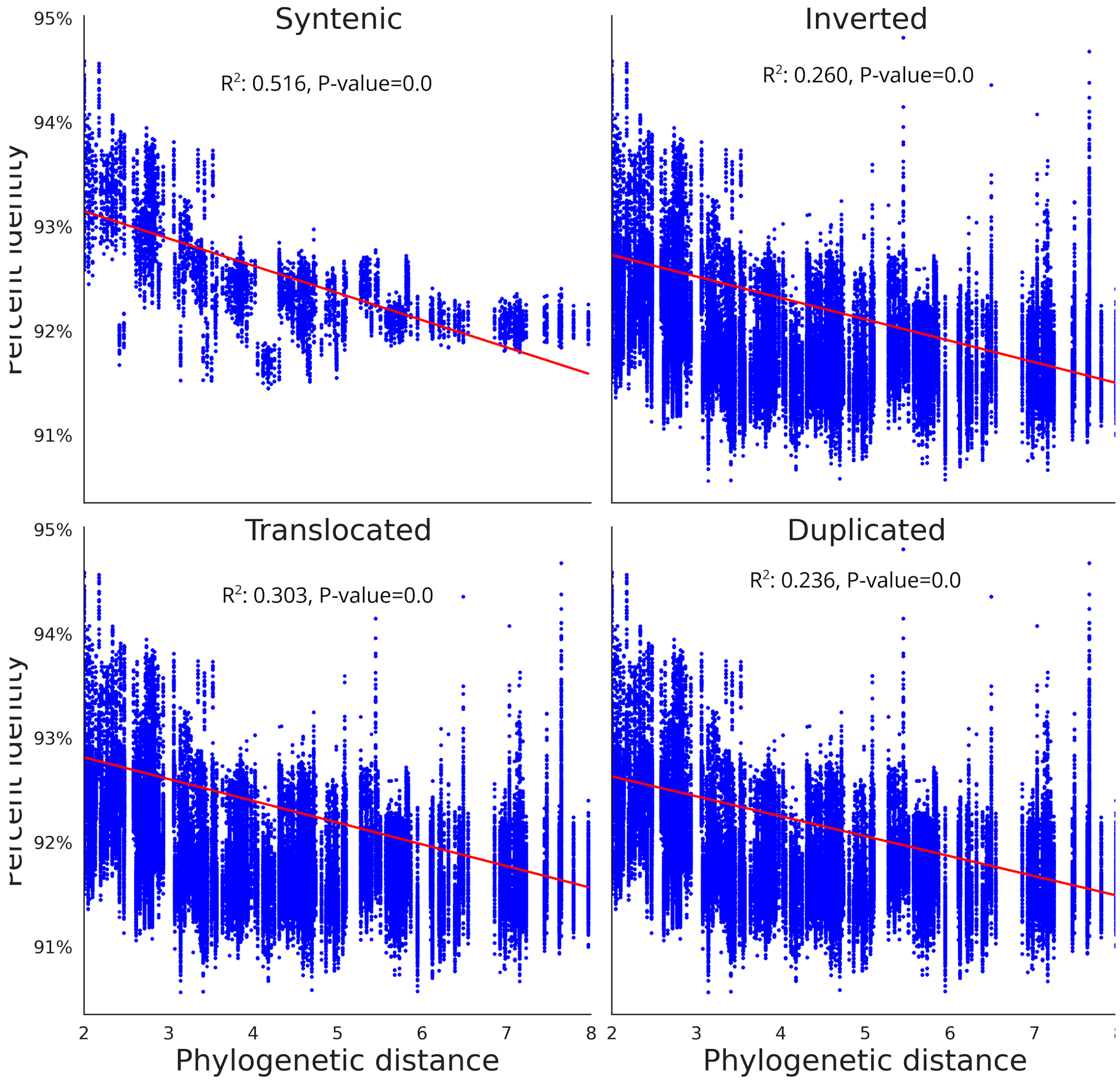

Supplementary Figure S20. MUMmer alignment percent identity regression and scatter plot.

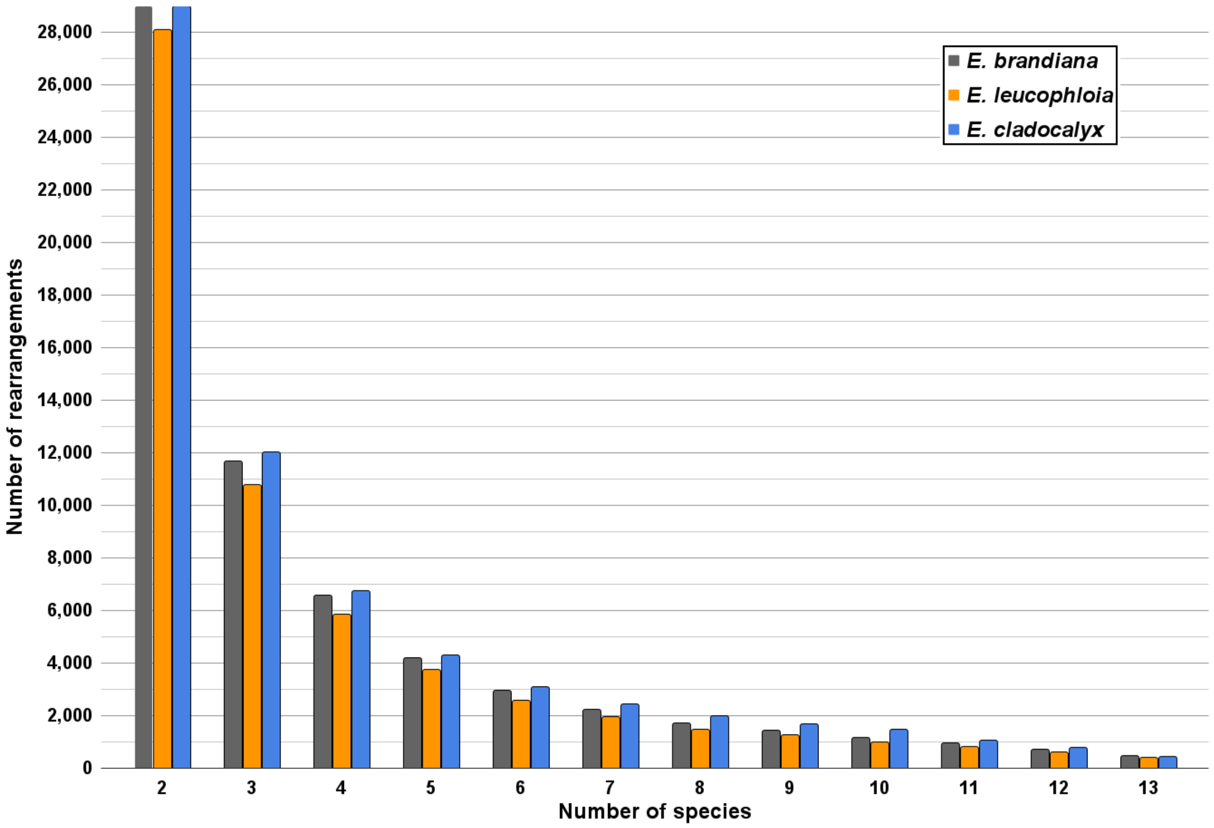

Supplementary Figure S21. Ignoring the phylogenetic relationships between genomes, shows the number of rearrangements shared by an increasing number of *Adnataria* genomes using *E. brandiana*, *E. cladocalyx*, and *E. leucophylla* as the outgroup/genetic architecture.

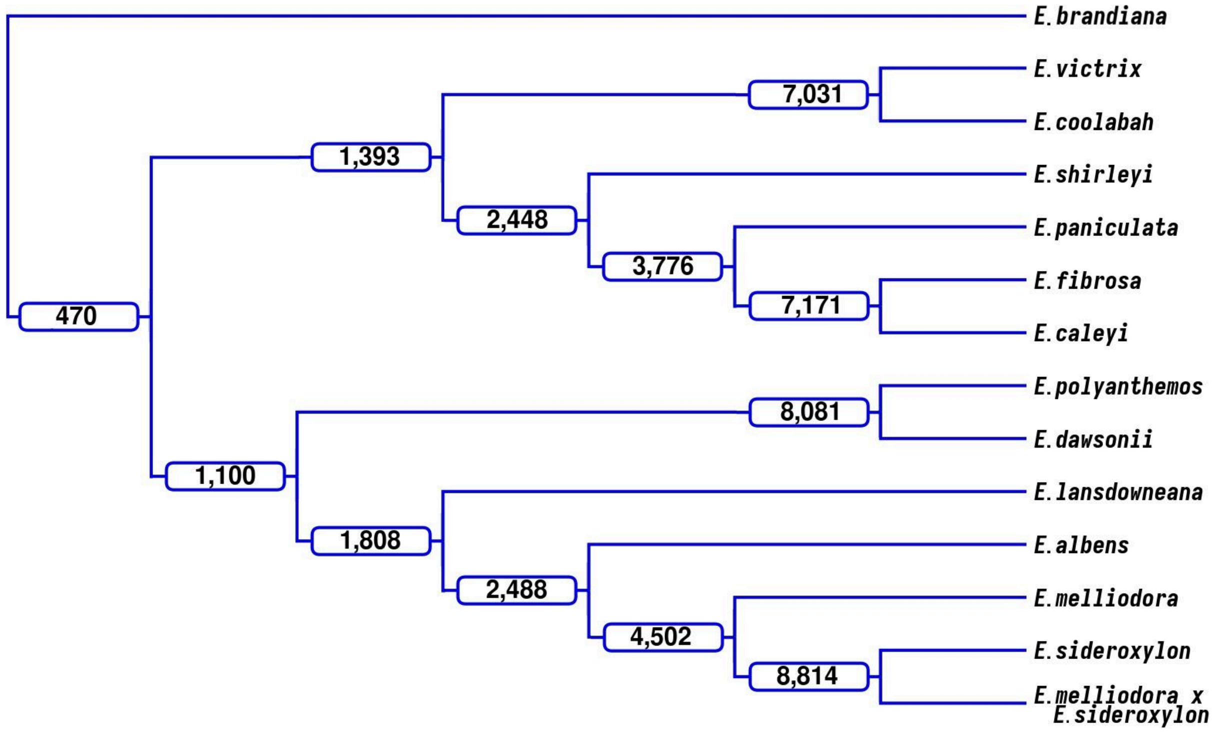

Supplementary Figures S22. Repeat of Adnataria phylogeny of common rearrangements. *E. brandiana* is the outgroup genome

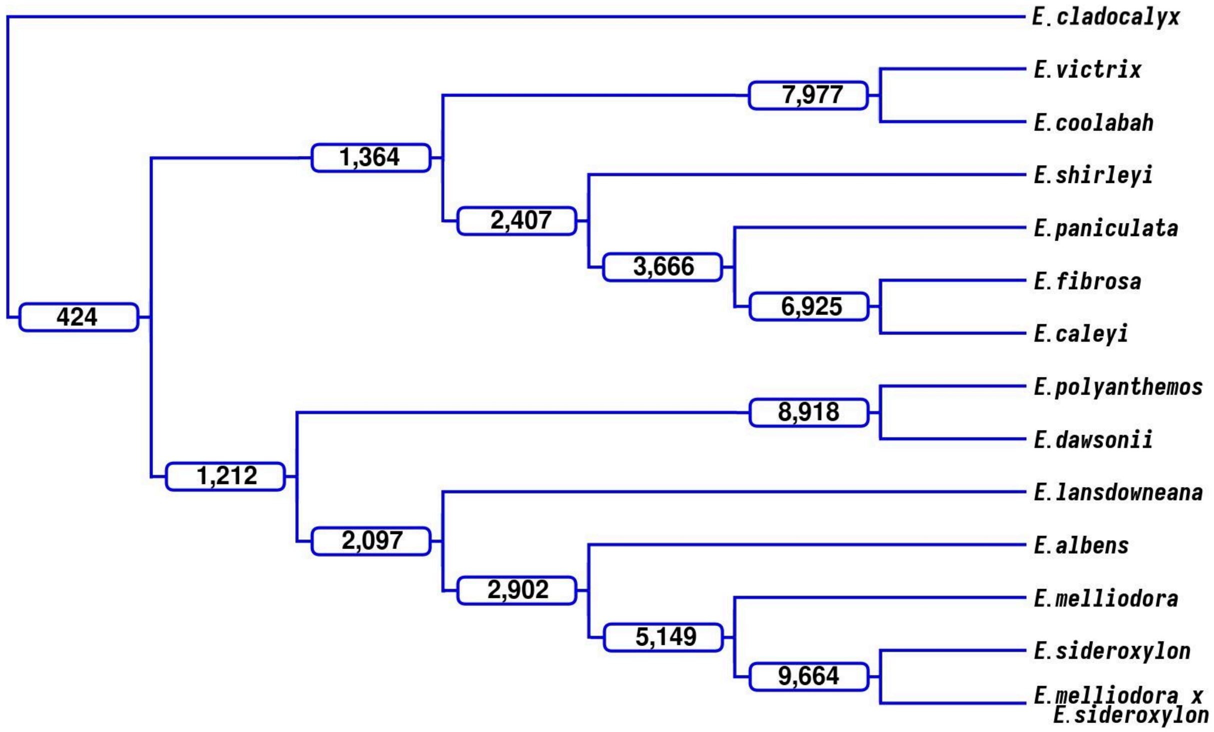

Supplementary Figures S23. Repeat of Adnataria phylogeny of common rearrangements. *E. cladocalyx* is the out genome.

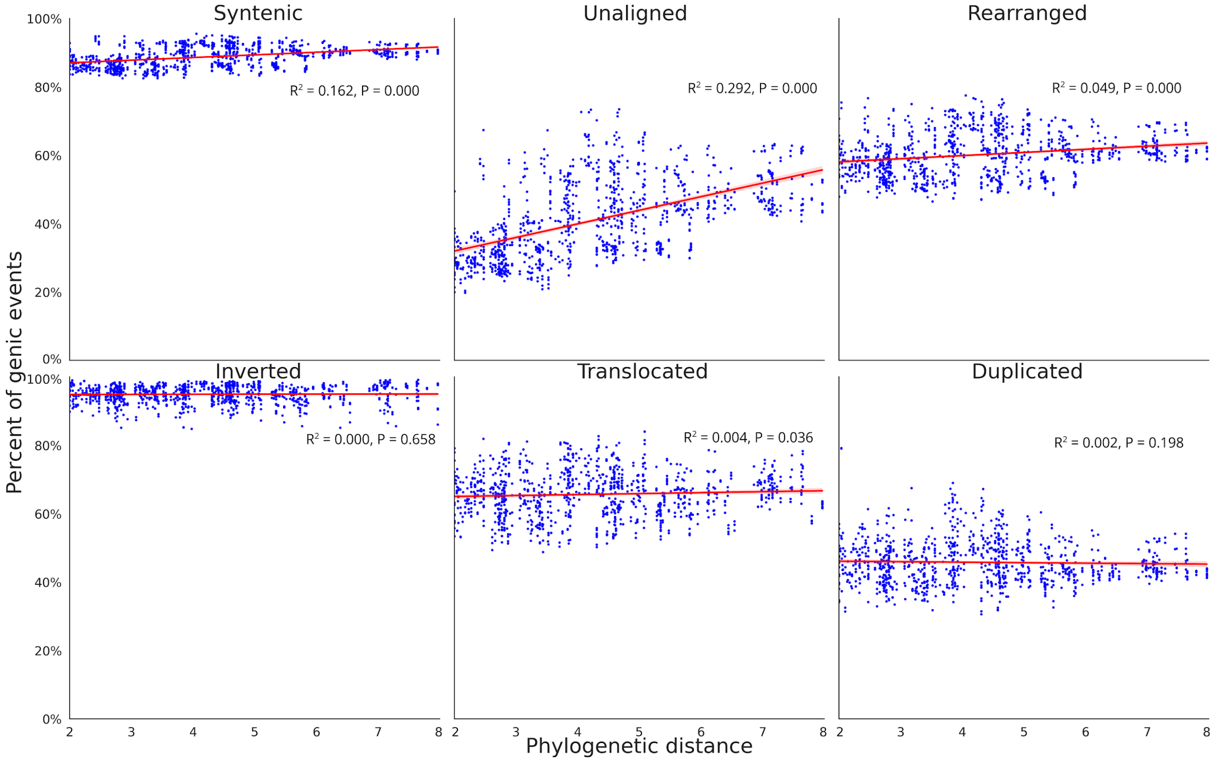

Supplementary Figures S24. Phylogenetic distance and the number of events syntenic, rearrangement, and unaligned events. Pairwise genome conservation and loss, as phylogenetic distance increases. The number of events between both Eucalyptus genomes with an alignment pair that was identified as syntenic, rearranged, or unaligned, plotted against the phylogenetic distance of the two genomes. The unaligned proportion is the species-specific fraction of the genome between genome pairs, resulting from either an insertion, deletion, differential inheritance, or sequence divergence. Rearranged events are broken down into inverted, translocated, and duplicated regions. Phylogenetic distance was calculated as the sum of branch lengths between each genome pair within phylogeny.
